# Toll-like receptor 4 deficiency in Purkinje neurons drives cerebellar ataxia by impairing the BK channel-mediated after-hyperpolarization and cytosolic calcium homeostasis

**DOI:** 10.1038/s41419-024-06988-w

**Published:** 2024-08-15

**Authors:** Jianwei Zhu, Wenqiao Qiu, Fan Wei, Jin Zhang, Ying Yuan, Ling Liu, Meixiong Cheng, Huan Xiong, Ruxiang Xu

**Affiliations:** 1 Department of Neurosurgery, Sichuan Provincial People’s Hospital, School of Medicine, University of Electronic Science and Technology of China, Chengdu, 610072 China; 2 Department of Critical Care Medicine, Mianyang Orthopaedic Hospital, Mianyang, Sichuan Province 621000 China

**Keywords:** Neuroscience, Diseases of the nervous system

## Abstract

Toll-like receptor (TLR) 4 contributes to be the induction of neuroinflammation by recognizing pathology-associated ligands and activating microglia. In addition, numerous physiological signaling factors act as agonists or antagonists of TLR4 expressed by non-immune cells. Recently, TLR4 was found to be highly expressed in cerebellar Purkinje neurons (PNs) and involved in the maintenance of motor coordination through non-immune pathways, but the precise mechanisms remain unclear. Here we report that mice with PN specific TLR4 deletion (TLR4^PKO^ mice) exhibited motor impairments consistent with cerebellar ataxia, reduced PN dendritic arborization and spine density, fewer parallel fiber (PF) – PN and climbing fiber (CF) – PN synapses, reduced BK channel expression, and impaired BK-mediated after-hyperpolarization, collectively leading to abnormal PN firing. Moreover, the impaired PN firing in TLR4^PKO^ mice could be rescued with BK channel opener. The PNs of TLR4^PKO^ mice also exhibited abnormal mitochondrial structure, disrupted mitochondrial endoplasmic reticulum tethering, and reduced cytosolic calcium, changes that may underly abnormal PN firing and ultimately drive ataxia. These results identify a previously unknown role for TLR4 in regulating PN firing and maintaining cerebellar function.

## Introduction

The toll-like receptor 4 (TLR4) contributes to the induction of neuroinflammation by binding pathogenic molecules and stimulating microglia to release pro-inflammatory cytokines. Non-immune cells also express TLR4, and several physiological signaling molecules, including neurotransmitters and neuromodulators, have been identified as TLR4 agonists or antagonists. Thus, there is growing interested in the non-immune functions of TLR4.

Recent studies suggest that TLR4 may contribute to neural development, plasticity, pain transmission, and various behaviorally relevant signaling processes in the central nervous system. For instance, TLR4 expression has been detected in adult neural progenitor cells (NPCs), and *TLR4* deletion was reported to enhance proliferation and neuronal differentiation in the hippocampus [1]. Similarity, deletion in neonatal mouse spinal cord NPCs promoted differentiation into long projecting neurons, although without influencing NPC proliferation [2]. Mature neurons also express TLR4, and ablation in stroke model animals was found to reduce cerebral volume and functional deficits [3]. Further, TLR4 upregulation in thalamic neurons was associated with hemorrhage-induced pain [4]. Specific deletion of TLR4 in dopamine neurons of the ventral tegmental area was reported to reduce the acquisition food reward [5]. Our previous study also revealed that TLR4 is highly expressed in mouse cerebellar Purkinje neurons (PNs) and that receptor deficiency leads to motor dysfunction [6] and reduced PN number. However, the underlying mechanisms are still unknown.

Purkinje neurons are the sole output cells of the cerebellar cortex and provide encoded information necessary for the coordinated execution of motor plans [7]. In addition, PNs are a major site for synaptic plasticity implicated in motor learning. Thus, PN dysfunction or degeneration usually results in a spectrum of motor function deficits called ataxias. The specific clinical manifestations of ataxia are dependent on the location of the abnormality but frequently include postural instability, gait disturbance, and motor discoordination [8, 9].

Each PN receives a huge number of synaptic inputs from cerebellar granule cell parallel fibers (PFs), stellate cells, and basket cells that convey inputs from other brain regions via deep cerebellar nucleic (DCN). These inputs are integrated by the extensively branched PN dendritic tree, and then sent output to other parts of the brain through these same DCN. The firing patterns of PNs instruct the execution of motor plans by modifying firing responses in the DCN. Thus, motor coordination is highly dependent on the temporal pattern of PN firing [10, 11].

Mature PNs generate both high-frequency sodium spikes and low-frequency calcium spikes, and the overall firing pattern is “trimodal” continuously cycling among tonic firing, bursting, and silent modes [12, 13]. The emergence of this “trimodal” firing pattern is concomitant with dendritic maturation, and arborization, as immature PNs generate predominantly a steady tonic firing pattern [14]. Therefore, abnormal dendritic arborization can also alter the firing pattern of PNs. When the firing pattern changes, PNs lose the ability to stably encode output information, which ultimately impairs motor coordination and results in ataxia [15]. The role of abnormal PN firing in ataxia has been well established through analysis of various mouse models. In the *tottering* mouse, a robust model of episodic ataxia type 2 (EA2), PNs exhibited stress-induced burst firing [16], while in the SCA2-58Q mouse, a model of spinocerebellar ataxia type 2 (SCA2), PNs exhibited irregular intrinsic pace-making and a large fraction predominantly exhibited burst firing [17, 18]. In the SCA3-84Q mouse model of SCA2, PNs showed loss of repetitive firing [19], while in the *Cacna1a*^S218L^ mouse, which demonstrates severe ataxia, PNs exhibited irregular firing and decrease firing rate [20].

The precision of intrinsic PN pace-making is maintained primary by the large conductance and small conductance Ca^2+^-activated K^+^ channels BK and SK, both of which are activated by voltage-dependent calcium influx during the action potential (AP) [21, 22]. The BK channel gene *KCNMA1* is highly expressed in PNs [23], and *KCNMA1* mutation or deficiency disrupts the normal firing patterns of PNs and rhythm in DCN, ultimately causing cerebellar ataxia [24–26]. The changes in firing pattern may be related to loss of the AP after-hyperpolarization (AHP) mediated largely by BK as inhibiting BK converted the AHP to an after-depolarization (ADP) and shifted the firing pattern from tonic to bursting [27].

It has been reported that BK channel currents are also activated by the bacterial toxin lipopolysaccharide (LPS) via TLR4 in microglia and macrophages, leading to inflammatory cytokine production [28, 29]. These findings suggest that TLR4 may also contribute to BK channel activation in PNs through non-immune pathways. To address this issue, we investigated the changes in PNs firing properties and motor function caused by PN-specific knockout of TLR4 (TLR4^PKO^) in mice. We report that the PNs of TLR4^PKO^ mice exhibit impaired dendritic arborization and spine development, reduced number of PF-PN and climbing fiber (CF)-PN synapses, downregulation of BK channel expression, decreased AHP amplitude and irregular firing patterns (which could be rescued with BK channel opener), lower cytoplasmic Ca^2+^ (cytoCa^2+^) concentration, and morphologically abnormal mitochondria. Further, these mice exhibited behavioral features typical of cerebellar ataxia. This study thus provides evidence that TLR4 regulates PN function through a non-immune pathway, and that loss of this regulation results in irregular firing, ultimately leading to cerebellar ataxia.

## Methods and materials

### Mice

Purkinje neuron specific TLR4 knockout (TLR4^PKO^) mice were generated by first breeding floxed mutant mice harboring *loxP* sites flanking exon 3 of the *Tlr4* gene (B6(Cg)-*Tlr4*^tm1.1Karp^/J, JAX stock number: 024872) (TLR4^flox/flox^ mice) with transgenic mice expressing *cre* inserted into exon 4 of *Pcp2* (B6.129-Tg(Pcp2-cre)2Mpin/J, JAX stock number: 004146) (Pcp2-Cre mice) to produce heterozygous Pcp2^Cre/+^; TLR4^flox/+^ mice. These heterozygous mice were then interbred to generate Pcp2^Cre/+^; TLR4^flox/flox(mutant)^ mice (TLR4^PKO^ mice) and Pcp2^Cre/+^; TLR4^+/+(wildtype)^ mice as controls (define as wildtype, TLR4^WT^). All mice were housed in temperature-controlled laboratory conditions under a 12 h:12 h light-dark cycle with free access to water and food. All housing and experimental protocols involving mice were approved by the Institutional Animal Care and Use Committee of Sichuan Academy of Medical Sciences and Sichuan Provincial People’s Hospital.

### Genotyping

Mice were genotyped by PCR using genomic DNA from tail clippings. The presence of *loxP* sequences was determined using PCR primers 5ʹ- TGACCACCCATATTGCCTATAC-3ʹ (forward) and 5ʹ- TGATGGTGTGAGCAGGAGAG-3ʹ (reverse), while *cre* sequence was detecting using primers 5ʹ- GCGGTCTGGCAGTAAAAACTATC-3ʹ (forward) and 5ʹ- GTGAAACAGCATTGCTGTCACTT-3ʹ (reverse). The internal positive control (IPC) PCR primers used were: 5ʹ- CTAGGCCACAGAATTGAAAGATCT-3ʹ (forward) and 5ʹ- GTAGGTGGAAATTCTAGCATCATCC-3ʹ (reverse). Products were resolved on 1.2% agarose gels. Then the animals were divided into TLR4^PKO^ group or TLR4^WT^ group according to the results of genotyped by PCR, instead of randomization.

### Behavioral analyses

All behavioral tests were performed on four-month-old TLR4^PKO^ mice and TLR4^WT^ littermates according to the schedule shown in Supplementary Figure 1A by investigators blind to mouse genotype.

### Hindlimb clasping test

Mice were suspended by the tail and the extent of hindlimb clasping was observed for 1 min as previously described [30]. Clasping score was defined as follows: 0, both hindlimb splayed outward away from the abdomen with splayed toes; 1, one hindlimb retracted or both hindlimbs partially retracted toward the abdomen and toes splayed; 2, both hindlimbs retracted toward the abdomen without touching each other; 3, both hindlimbs fully clasped and touching the abdomen.

### Accelerating-rotarod test

Mice were placed on the rotarod facing opposite to the direction of rotation. The rotarod was started at 4 revolutions per minute (rpm) for 10 s, then accelerated to 40 rpm in 4 min and maintained at 40 rpm for 1 min. The latency to fall off and maximum rotation speed at the time of falling were recorded during each 5-min trial. Three trials were performed for each mouse and averaged 3 times.

### Footprint test

The forepaws and hindpaws of all tested mice were painted with nontoxic red and black ink, respectively. Each mouse was then allowed to cross a tunnel (70 cm length, 10 cm width, 10 cm height) with white paper covering the floor. For each of seven successive paws prints, a point was identified at the base of the middle toe. Forepaw and hindpaw stride lengths were defined as the straight-line distances through successive points of contact by the ipsilateral forepaw and hindpaw, respectively. Forepaw and hindpaw stride widths were defined as the perpendicular line distances between points of contact and a line connecting successive contralateral forepaw and hindpaw prints, respectively. Inter-limb coordination was defined as the straight-line distance between the hindpaw point of contact and the adjacent ipsilateral forepaw point of contact. Mice that did not move in the tunnel were excluded, while trails were repeated for mice that stopped in the middle of the tunnel. The first and last 10 cm segments of the print were excluded from all analyses. Each mouse performed three pre-trials before the measured trials.

### Beam walk test

Mice were trained to traverse an open rod-shaped beam (60 cm in length, 1.5 cm in diameter) set 30 cm above the ground over a 2-day training period. During actual trials, the time taken to traverse the entire beam was recorded to a maximum of 2 min. In addition, the number of paws slips was recorded. If the paws clasped the beam when walking, the slip number was recorded as 2. If the mouse fell off within the first quarter of the beam length, the time was recorded as 60 s.

### Ledge test

Mice were placed on a narrow ledge (60 cm long, 0.6 cm wide, 5 cm high) 30 cm above the ground and allowed to travel freely. The time spent crossing the ledge and the numbers of paw slips were recorded. If the paws clasped the ledge while walking, the slip number was recorded as 2. Mice that stopped moving on the ledge were excluded.

### Hangwire test

Balance and grip strength were analyzed by the hangwire test as previously described [6]. Briefly, mice were placed on a wire screen with 12 mm × 12 mm grids, which was then inverted and raised 30 cm above a mouse cage. The latency to fell off was recorded within the 120 s trial period. If the mouse did not fall off within 120 s, a maximum time of 120 s was assigned.

### Traction test

Mice were first weighted and then allowed to grasp a transducer bar (1 mm diameter) with both forepaws and slowly pulled backward by the tail. The maximum tension (in grams) before forepaw release was recorded and normalized to body weight. Each mouse performed three trials with a minimum 10-min recovery period between trials.

### Preparation of single-cell suspensions from mice brain tissue

Brain tissues were collected, rinsed three times with sterile phosphate buffered saline (PBS), and transferred (maximum of 200 mg per sample) to gentleMACS C Tubes containing 4.9 mL HEPES buffer, 100 μL Collagenase D solution (2 mg/mL), and 10 μL DNase I solution (40 U/mL). Tissues were incubated for 30 min at 37 °C with automated rotation in the gentleMACS Dissociator. Treatments were controlled by gentleMACS programs “R_brain_01”, “R_brain_02”, and “R_brain_03”. Cells were then filtered through mesh cell strainers (70 μm) and collected in 50 mL centrifuge tubes. Cells remaining in the strainers were recovered by washing with 5 mL HEPES buffer at room temperature (RT). The resulting cell suspensions were centrifuged at 300 × *g* for 10 min. After removal of the supernatant, the cells were resuspended in the desired volume of protein extraction buffer (PEB) for single cell sequencing.

### Gene set enrichment analysis (GSEA) and pathway enrichment analysis

Enrichment analysis of genes differentially expressed between knockout and WT mice was conducted using the Molecular Signatures Database GO gene-sets (C5) and the R package ClusterProfiler [31] with a cut-off *P* of 0.05. Differentially expressed genes were annotated for the Canonical Pathway (MSigDB), Hallmark Gene Sets (MSigDB), KEGG Pathway, and GO Biological Processes using Metascape (http://metascape.org) [32]. The detailed analysis results are shown in Data S1.

### Protein protein interaction network analysis

The differential expression results were integrated with the human STRING v10 protein protein interaction network [33] using the ‘STRINGdb’ R package from Bioconductor [34]. The raw networks were pruned to include only high-confidence interactions (i.e., interactions with combined scores of 700 or greater). The probabilities of differential expression were assigned to the corresponding nodes, and edge weights were calculated as the product of the posterior probabilities of incident nodes. Thus, edge weights represent the joint posterior probability of differential expression between the interacting proteins. The networks were further pruned to include only edges with weight ≥0.98. The giant component of the resulting network is the differentially regulated portion of the protein protein interaction network. Communities in these networks were identified using the Louvain algorithm implemented in the ‘igraph’ R package.

### Gene set variation analysis

To compare signaling pathway enrichment among cell clusters, gene set enrichment scores were calculated using GSVA [35]. Briefly, we ran the gsva function for each single cell, used the “ssGSEA” method to calculate the enrichment score of indicated gene sets, and then used limma packages (version 3.52.3) to analyze differences in enrichment score for every gene set between each cell cluster.

### Cerebellar slice electrophysiology

One-month-old TLR4^PKO^ mice and TLR4^WT^ littermates were anesthetized with isoflurane and perfused through the heart with ice-cold cutting solution containing (in mM) 5 KCl, 1.25 NaH_2_PO_4_, 26 NaHCO_3_, 212.7 sucrose, 10 glucose, CaCl_2_, and MgCl_2_ (pH = 7.3–7.5, 292–320 mOsm). The brain was quickly removed and regions of cerebellum dissected. Sagittal sections of the cerebellum (300-μm thick) were prepared in ice-cold cutting solution aerated with 5% CO_2_ + 95% O_2_ and transferred into aCSF containing (in mM) 5 KCl, 124 NaCl, 1.25 NaH_2_PO_4_, 10 Glucose, 26 NaHCO_3_, 2.4 CaCl_2_, 1.2 MgCl_2_ (pH = 7.3–7.5, 292–320 mOsm) aerated with 5% CO_2_ + 95% O_2_ for 30 min at 32 ^o^C. Slices were stored at room temperature for at least 30 min and then placed into a recording chamber. Slices were continuously perfused with aCSF maintained at 32 ^o^C and aerated with the same gas mixture during recordings.

For the whole-cell patch-clamp recording, PNs both in anterior and posterior lobules were identified by patch-clamp recording. Borosilicate glass pipettes (1.5 mm outside diameter, 0.9 mm inner diameter, 6 MΩ) were filled with internal recording solution containing (in mM) 120 K-gluconate, 0.3 Na_2_GTP, 2 Na_2_ATP, 2 MgCl_2_, 1 EGTA, 10 HEPES (pH = 7.25–7.35, 292–308 mOsm). Whole-cell current clamp recording were acquired at 32 ^o^C in aCSF 1–5 h after slice preparation using an Axoclamp 700B amplifier and Digidata 1550B interface under the control of pClamp 10.8 software. When performing BK channel opener experiments, the TLR4^PKO^ cerebellum slices were incubated with aCSF containing 10 μM BMS-191011 (HY-108593, MedChemExpress).

All signals were filtered at 2 kHz. After GΩ-seal formation and membrane break-through, the membrane resting potential was monitored for 10 min to allow stabilization before recording action potentials in current-clamp mode. Action potentials amplitude was defined as the sum of the absolute value of the resting potential and the maximum spike value. AHP amplitude as the absolute value of maximum hyperpolarization minus the absolute resting potential value, AHP duration as the time taken for the membrane potential to return to the resting potential after each AP, and AHP slope (V/s) from the maximum hyperpolarization to the start of the next action potential as previously described [36].

### Histological and immunohistochemical analysis

Four-month-old TLR4^PKO^ mice and TLR4^WT^ littermates were deeply anesthetized and perfused through the heart with PBS followed by 4% paraformaldehyde (PFA) in PBS. The brain was removed and stored in 4% PFA overnight at 4 °C and then dehydrated in 30% sucrose for 72 h at 4 °C. Cerebellar sagittal sections (20 μm thick) were cut using cryostat and mounted onto glass slides. For histopathology, sections were stained with hematoxylin and eosin (H&E) using a conventional staining protocol. For immunohistochemical staining, sections were air dried, rinsed with PBS, permeabilized by incubation in 0.3% Triton X-100 in PBS for 30 mins (3 times × 10 min), blocked by incubation in 10% bovine serum albumin (BSA) for 1 h at RT, incubated with the primary antibodies (Table S1) overnight at 4 °C, washed three times with 0.3% Triton X-100 in PBS (10 min per wash), and incubated in Alexa Fluro^TM^-conjugated secondary antibodies for 2 h at RT. Sections were mounted with medium containing DAPI (Beyotime Biotechnology, Cat #P0131, Shanghai, China) for counter staining of nuclei and imaged using a confocal fluorescence microscope.

### Golgi–Cox staining

Four-month-old TLR4^PKO^ mice and TLR4^WT^ littermates were deeply anesthetized and perfused through the heart with PBS. The cerebellum was quickly removed and prepared for Golgi**–**Cox staining as instructed by the FD Rapid Golgi Stain^TM^ Kit manufacturer (FD Neurotechnologies, Columbia, USA). Cerebellar sagittal sections were then prepared at 100 μm and mounted onto glass slides, followed by Golgi staining according to the kit manufacturer’s instructions. Images were captured by confocal microscopy at 100 × using an oil-emersion objective lens.

### Stereotaxic injection of viral vectors into the cerebellum

Three-month-old TLR4^PKO^ and TLR4^WT^ mice were anesthetized with a mixture of O_2_ and isoflurane (2% dosage for induction, 1% for maintenance) and the head fixed on a stereotaxic frame. Three injection sites were chosen in vermis cerebelli, and 1 μL of pAAV-L7-NES-jRGECO1a virus suspension (1 × 10^12^ vg/mL) was injected into each site at an infusion rate of 0.25 μL/min using a 5-μL Hamilton syringe. The micropipette was retracted by 0.5 mm at 1-min intervals during injection and kept within the tissue for 4 min in total before full removal to facilitate diffusion of the vector. After three weeks, mice were sacrificed and the cerebellum removed to prepare frozen sections of vermis. The fluorescent signal from pAAV-L7-NES-jRGECO1a in PNs was captured by confocal microscopy and intensity quantified using Image J software.

### Electron microscopy

Four-month-old and one-month-old TLR4^PKO^ and TLR4^WT^ mice were deeply anesthetized with isoflurane and perfused intracardially with 0.9% saline and then with 2%/2% PFA/glutaraldehyde solution. The Cerebellum was removed and post-fixed in 2%/2% PFA/glutaraldehyde solution for 72 h at 4 °C, followed by post-fixation with 1% osmium tetroxide for 2 h. Cerebellar tissue were then dehydrated in gradient ethanol, embedded in Epoxy resin, and cut into ultrathin sections (100 nm thickness). Sections were stained with uranyl acetate citrate solution and imaged by transmission electron microscopy (Hitachi, H-7650, Japan) to examine the morphological features of PN mitochondria.

### Quantitative analysis of PN number, synaptic connectivity, and dendritic arborization

For quantification of PNs number/density, sagittal sections prepared as described were immunostained with calbindin antibody. Sections at the same level of each mouse were then selected and photographed under confocal fluorescence microscopy (Zeiss, LSM800) or slide scanner (Olympus, VS200). On each image obtained with confocal fluorescence microscopy, a 200 μm line was drawn along the Purkinje cell layer (PCL) and calbindin-positive cells were counted along this length using Image J [30]. On images obtained with slide scanner, the calbindin-positive cells were counted and the length of the PCL in each lobule was calculated with Olympus OlyVIA software.

For quantification of vGlut1-positive PF–PN synapses, the mean fluorescence intensity (MFI) of vGlut1 immunostaining signal in a 50 μm^2^ area of the molecular layer (ML) of each section was measured using Image J. For quantification of vGlut2-positive CF–PN synapses, vGlut2-positive puncta were counted along a 50 μm length of climbing fiber (CF) using Image J.

For quantification of PN dendrite tree area, 10 to 15 Golgi-stained PNs in sections were examined using Image J as previously described [30]. For analysis of PN dendrite arborization, Sholl analysis was performed using ImageJ [30]. For quantification of PN spine density, spine number along 10-μm lengths of dendrite were counted manually with Image J assistance.

### Statistical analysis

All data are presented as mean ± standard error of the mean (SEM). Data with values outside the mean plus or minus 2 times the standard deviation will be excluded. Group means were compared by independent samples Student’s test using PASW Statistics 20.0 software (SPSS Inc., Chicago, USA). Multiple groups were compared by two-way ANOVA followed by Tukey’s post hoc multiple comparison test using GraphPad Prism 7.0 statistical software. A *P* < 0.05 (two-tailed) was considered statistically significant for all tests.

## Results

### TLR4 expression was ablated in cerebellar Purkinje neurons of TLR4^PKO^ mouse

**Fig. 1:**
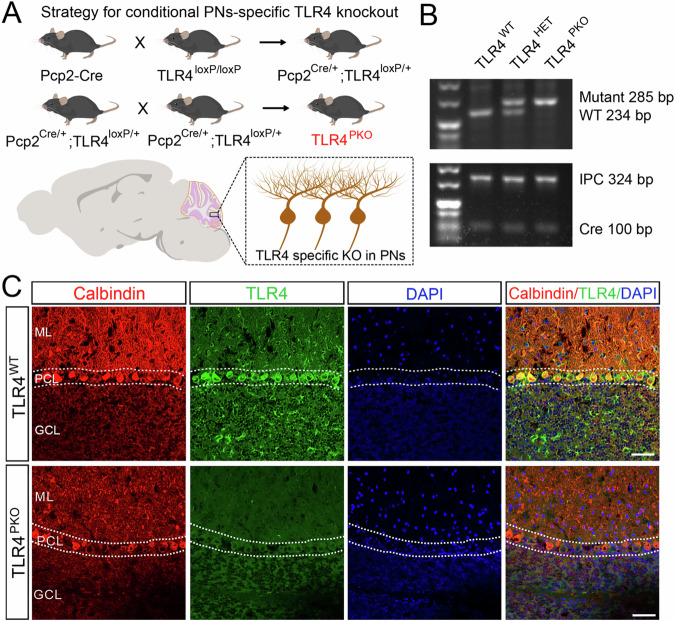
Generation of Purkinje neuron (PN)-specific Toll-like receptor 4 (TLR4) knockout mice (TLR4^PKO^). **A** Breeding strategy for generation of TLR4^PKO^ mice from TLR4^loxp/loxp^ mutant and Pcp2-Cre transgenic mice. **B** Genotyping to distinguish TLR4^PKO^ from TLR4^WT^ mice using PCR and agarose gel electrophoresis. Shown are gel bands for TLR4 mutant, TLR4 wildtype, IPC, and Cre. **C** Representative confocal images of a cerebellar sagittal section immunostained with specific TLR4 and calbindin antibodies. TLR4 was robustly expressed in (calbindin-positive) PNs and sporadically in the granule cell layer (GCL) of TLR4^WT^ cerebellum, while no significant TLR4 expression was found in PNs of TLR4^PKO^ cerebellum. In contrast, fluorescent signals were still detected in the GCL of TLR4^PKO^ cerebellum. Scale bar = 50 μm (**C**). HET heterozygote, IPC internal positive control, ML molecular layer, PCL Purkinje cells layer, GCL granule cell layer.

To examine if TLR4 signaling contributes to PN development, circuit formation, electrophysiological properties, and (or) motor control, we produced PN-specific TLR4 knockout mice (TLR4^PKO^) by crossing TLR4 mutant mice with *loxP* sites flanking *Tlr4* exon 3 and Pcp2-Cre transgenic mice expressing Cre recombinase in PNs (Fig. 1A) and some retinal bipolar neurons, and small amounts of unidentified population of cells in the cerebrum [37]. Throughout these studies, mouse genotype was distinguished by PCR and electrophoresis-based detection of TLR4^mutant^, TLR4^wildtype^, and Cre (Fig. 1B).

Immunofluorescence staining of cerebellar sections revealed that TLR4 was highly expressed in calbindin-positive PNs of TLR4^WT^ cerebellum but not TLR4^PKO^ cerebellum (Fig. 1C). According to our breeding program, we speculated that the TLR4 is knockout exclusive to PNs in cerebellum and some other neurons out of cerebellum, while still expressed in cerebellar microglial cells of both TLR4^WT^ and TLR4^PKO^ cerebellum, consistent with previous reports [38].

### TLR4^PKO^ mice exhibited cerebellar ataxia

**Fig. 2:**
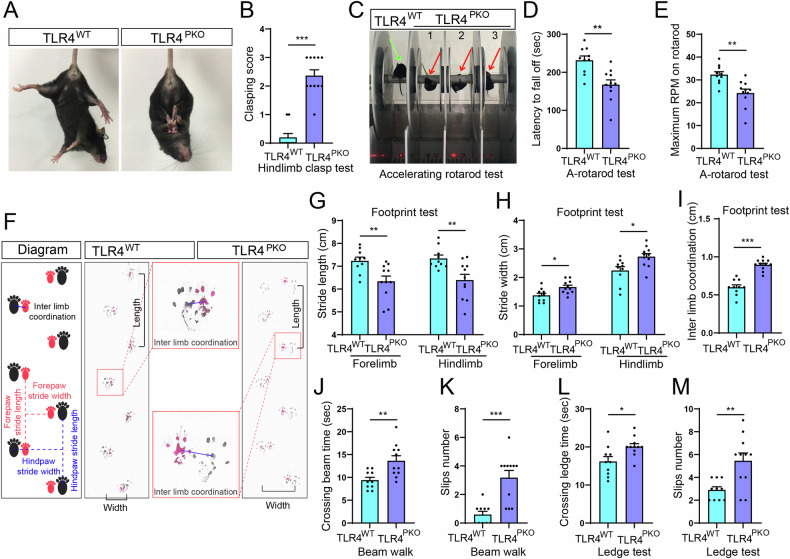
Ataxic behaviors of TLR4^PKO^ mice. **A**, **B** TLR4^PKO^ mice exhibited ataxic hindlimb clasping. **A** Representative images of the hindlimb clasping phenotype frequently observed in ataxia model mice. When suspended by the tail, TLR4^WT^ mice splayed all toes and both hindlimbs (away from the abdomen), while TLR4^PKO^ mice showed hindlimb clasping. **B** Clasping score was higher in TLR4^PKO^ mice. **C** Representative image of motor performance in the accelerating-rotarod test. TLR4^PKO^ mice could not stand steadily on the rotarod, unlike TLR4^WT^ mice (green arrow), but clung to the rotarod to avoid falling (red arrows). Latency to fall off the rotarod was shorter (**D**) and maximum rotation (rpm) before falling (**E**) slower among TLR4^PKO^ mice compared to TLR4^WT^ mice. **F**–**I** TLR4^PKO^ mice also exhibited gait disturbances. **F** Diagram of footprint analysis for gait and representative paw prints acquired from footprint tests. TLR4^PKO^ mice exhibited shorter stride length (**G**), greater stride width (**H**), and longer inter limb coordination (**I**). **J**–**M** TLR4^PKO^ mice also demonstrated impaired balance. Beam-crossing time was prolonged (**J**) and paw slips more numerous (**K**) among TLR4^PKO^ mice during the beam test. Ledge-cross time was also longer (**L**) and paw slips more numerous (**M**) among TLR4^PKO^ mice during the ledge test. Each point in the histogram represents a mouse. Data are represented as mean ± SEM, * *p* < 0.05; ** *p* < 0.01; *** *p* < 0.001.

Ataxia is a characteristic symptom of PN dysfunction. Our previous study showed that global TLR4 knockout impaired balance and motor coordination [6], suggesting that TLR4 may be essential for PN function in motor control. Indeed, TLR4^PKO^ mice showed hindlimb clasping when suspended by the tail, a behavioral response typical of ataxia model mice [39], while TLR4^WT^ mice exhibited hindlimb extension and sprayed toes typical of mice without ataxia (Fig. 2A). Further, clasping score was also significantly higher in TLR4^PKO^ mice than TLR4^WT^ mice (Fig. 2B). In the accelerating-rotarod test, TLR4^WT^ mice ran steadily on the rotarod, while TLR4^PKO^ mice clung to the rotarod to avoid falling at the same rotational speed (Fig. 2C, video S1). The latency to fall off the rod was also significantly shorter (Fig. 2D) and the maximum rotation rate significantly reduced (Fig. 2E) in the TLR4^PKO^ group compared to the TLR4^WT^ group. In addition, TLR4^PKO^ mice exhibited prominent gait abnormalities. Footprint analyses of stride length, stride width, and inter-limb coordination, three core parameters for assessment of normal versus abnormal gait [40, 41], are illustrated in the left panel of Fig. 2F while typical footprints of TLR4^PKO^ and WT mice are shown in the right panel. Both hindlimb and forelimb stride length were significantly reduced by TLR4 knockout (Fig. 2G), while hindlimb and forelimb stride width (Fig. 2H) and inter-limb coordination (Fig. 2I) were significantly increased compared to TLR4^WT^ mice. Furthermore, TLR4^PKO^ mice exhibited poor performance on both the beam walk test (video S2) and ledge test (video S3). Quantitative analysis revealed that TLR4^PKO^ mice took a significantly longer time to cross both the beam and ledge (Fig. 2J, L) with more limbs slips number than TLR4^WT^ mice (Fig. 2K, M). In contrast, TLR4^PKO^ mice demonstrated no significant impairments in skeletal muscle strength, an important factor affecting motor performance [42], as measured by the traction test (Supplementary Figure 1B) and hangwire test (Supplementary Figure 1C). These results suggest that deletion of TLR4 from cerebellar PNs leads to cerebellar ataxia without impairing skeletal muscle development.

### Specific deletion of TLR4 in PNs leads to reduced PN number, dendritic arborization and spine density

**Fig. 3:**
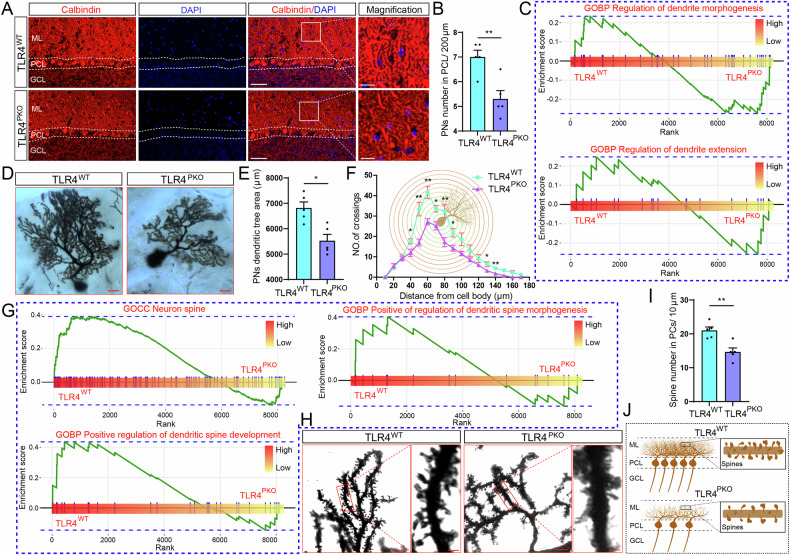
Purkinje cell-specific TLR4 deletion reduced PN number, dendritic arborization, and spine density. **A** Representative confocal images of a cerebellar sagittal sections immunostained for calbindin to reveal PNs. **B** Quantitative analysis showing reduced PN number in the TLR4^PKO^ cerebellum compared to TLR4^WT^ cerebellum. **C** Top dendrite-related Gene Ontology (GO) Biological Process pathways downregulated in TLR4^PKO^ cerebellum. The y-axes represent the GSVA score, which is a pathway-level quantification of gene abundance, and the x-axes represent the rank value. Compared to TLR4^WT^ mice, expression levels of genes related to ‘dendrite morphogenesis’ and ‘regulation of dendrite extension’ were downregulated in TLR4^PKO^ mouse cerebellum. **D**–**I** PN dendrite arborization and spine development were reduced in TLR4^PKO^ mice. **D** Representative images of PNs revealed by Golgi–Cox staining. **E** Reduced PN dendritic tree area in TLR4^PKO^ cerebellum. **F** Sholl analysis also revealed reduced PN dendritic arborization in TLR4^PKO^ cerebellum. **G** The top neuronal dendritic spine-related GO Cellular component and Biological process pathways downregulated in TLR4^PKO^ cerebellum compared to TLR4^WT^ cerebellum. The y-axes represent the GSVA score and the x-axes represent the rank value. Compared to TLR4^WT^, pathways related to ‘regulation of neuron spine’, ‘positive regulation of dendritic spine morphogenesis’ and ‘positive dendrite spine development’ were downregulated in TLR4^PKO^ cerebellum. **H** Representative images of PN dendritic spines revealed by Golgi–Cox staining. **I** Reduced PN dendritic spine density in TLR4^PKO^ cerebellum. **J** Schematic illustration summarizing these results. Scale bar = 50 μm in low magnification images and 10 μm in high magnification images (**A**); 10 μm (**D**); 5 μm in low magnification images and 1 μm in high magnification images (**H**). Each point in the histogram represents a mouse. Data are represented as mean ± SEM, * *p* < 0.05; ** *p* < 0.01; *** *p* < 0.001. The significance threshold was also set to an adjusted *p* < 0.05 (**C**, **G**). ML molecular layer, PCL Purkinje cell layer, GCL granule cell layer, CC cellular component, BP biological process.

Cerebellar ataxia is usually caused from PN dysfunction, and normal PN function is dependent on numerical sufficiency and appropriate integration into cerebellar circuitry. In addition, morphology is the structural basis for many neuronal functions, such as synaptic integration. Therefore, we first examined changes in PN number, connectivity, and morphology induced by TLR4 knockout. PN-specific deletion of TLR4 had no significant effect on cerebellar size (Supplementary Fig. 2A, B) or foliation and gross cytoarchitecture (Supplementary Fig. 2C). However, immunostaining for calbindin to label PNs (Fig. 3A) revealed reduced PN number in the PCL of TLR4^PKO^ mice compared to TLR4^WT^ littermates (Fig. 3B). We also calculated the PN numbers in different lobules, found the same decrease in lobule VII and X (Supplementary Fig. 2D).

Single-cell mRNA sequencing for identification of differentially expressed genes (DEGs) between genotypes and subsequent enrichment analysis revealed that the top upregulated pathways related to dendrite (ranked by normalized enrichment score, NES) included ‘regulation of dendrite morphogenesis’ and ‘regulation of dendrite extension’ (Fig. 3C), suggesting possible dendrite abnormalities induced by TLR4 deficiency. To evaluate possible morphological abnormalities, we visualized the dendritic tree of PNs using Golgi–Cox staining and quantified both dendrite size and complexity. The dendritic trees of Golgi–Cox-stained PNs appeared smaller in TLR4^PKO^ mice compared to TLR4^WT^ mice (Fig. 3D), and this was confirmed by quantitative analysis of dendritic tree area (Fig. 3E). In addition, counting of dendritic branch points at various distances from the soma (Sholl analysis) revealed reduced arborization of middle and distal dendritic branches in TLR4^PKO^ PNs compared to TLR4^WT^ PNs (Fig. 3F). However, we calculated the ML thickness using ImageJ software as previously described [6], and found the thickness of the ML was the same in the TLR4^PKO^ and TLR4^WT^ mice (Supplementary Figure 2E).

Further bioinformatics analysis revealed that the top upregulated pathways related to neuron as ranked by the NES included ‘neuron spine’, ‘positive regulation of dendritic spine morphogenesis’, and ‘positive regulation of dendritic spine development’ (Fig. 3G), suggesting additional deficits in dendritic spine density or distribution. Consistent with these gene enrichment analyses, Golgi–Cox staining revealed fewer spines on TLR4^PKO^ PN dendrites compared to TLR4^WT^ PN dendrites (Fig. 3H), and this finding was confirmed by analyzing spine density (Fig. 3I). These reductions in PN number, dendritic branch complexity, and spine density (Fig. 3J) suggest that TLR4 is involved in maintaining PN survival, dendritic growth and plasticity, and spine dynamics, and further that a TLR4 signaling deficits may contribute to ataxia by impairing synaptic integration and PN output.

### Specific TLR4 deletion in PNs reduces PF-PN synapses and CF-PN synapse numbers

**Fig. 4:**
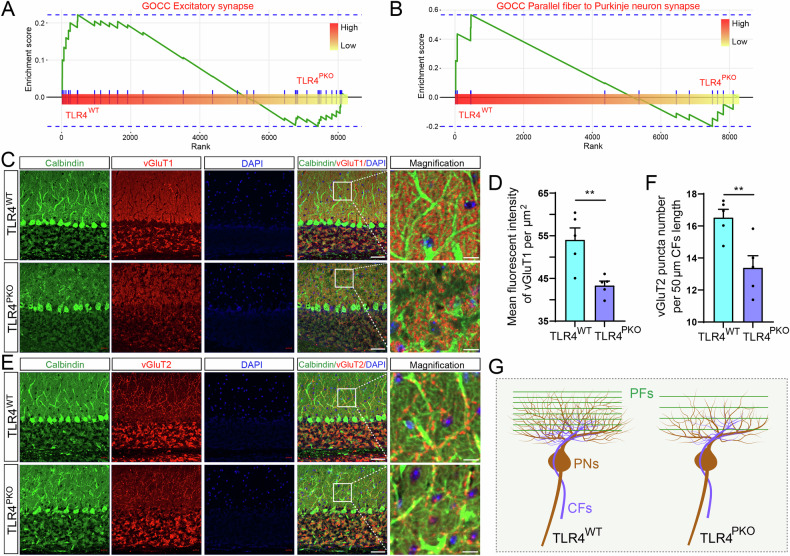
Purkinje neuron-specific deletion of TLR4 reduced parallel fiber (PF)–PN synapse and climbing fiber (CF)–PN synapse numbers. **A**, **B** The top synapse-related GO Cellular component pathways downregulated in TLR4^PKO^ cerebellum. The y-axes represent the GSVA score and the x-axes represent the rank value. **C**, **D** Compared to WTs, PF–PN synapse number was reduced in TLR4^PKO^ cerebellum. **C** Representative confocal images of a cerebellar sagittal section immunostained for calbindin antibody as a PN marker and vGluT1 antibody as a marker of PF–PN synapses. **D** Reduced mean fluorescence intensity of vGluT1 in TLR4^PKO^ cerebellum, confirming reduced PF–PN synapse number. **E**, **F** Compared to TLR4^WT^ cerebellum, CF–PN synapse number was reduced in TLR4^PKO^ cerebellum. **E** Representative confocal images of a cerebellar sagittal section immunostained for calbindin and vGluT2 as a CF–PN synapse marker. **F** Reduced number of vGluT2-positive puncta in TLR4^PKO^ cerebellum. **G** Schematic illustration summarizing these results. Scale bar = 50 μm in low magnification images and 10 μm in high magnification images (C, D). Each point in the histogram represents a mouse. Data are represented as mean ± SEM, * *p* < 0.05; ** *p* < 0.01. The significance threshold was set to an adjusted *p* < 0.05 (**A**, **B**).

Excitatory PF-PN and CF-PN synapses are formed by PFs and CFs at the distal and proximal dendritic spines of PNs, respectively, and both form neural circuit loops essential for processing PN inputs and outputs to the DCN. The top upregulated pathways related to ‘synapse’ ranked according to NES were related mainly to excitatory synapses (Fig. 4A), especially PF**–**PN synapse pathways (Fig. 4B), suggesting abnormalities in PF**–**PN and CF**–**PN synaptic transmission in TLR4 KO mice. We directly evaluated this possibility by immunostaining for the PF–PN synapse marker vesicular glutamate transporter 1 (vGluT1) and the CF**–**PN synapse marker vesicular glutamate transporter 2 (vGluT2). The number of vGluT1-positive puncta was greater in the distal dendritic fields (ML) of TLR4^WT^ mouse cerebellum (Fig. 4C), consistent with this result, the MFI of vGluT1 was also greater in TLR4^WT^ than TLR4^PKO^ ML (Fig. 4D). Similarly, vGluT2-positive puncta were more numerous in the proximal dendritic fields of TLR4^WT^ PNs than TLR4^PKO^ PNs (Fig. 4E). Quantitative analysis also showed greater vGluT2-positive puncta density in the vicinity of TLR4^WT^ PNs than TLR4^PKO^ PNs (Fig. 4F). These results suggest that dendritic spine density is reduced by PN-specific TLR4 knockout (Fig. 4G). However, the underlying mechanisms require further elucidation.

### Single-cell RNA sequencing (scRNA-seq) revealed extensive transcriptomic changes in TLR4^PKO^ cerebellum

**Fig. 5:**
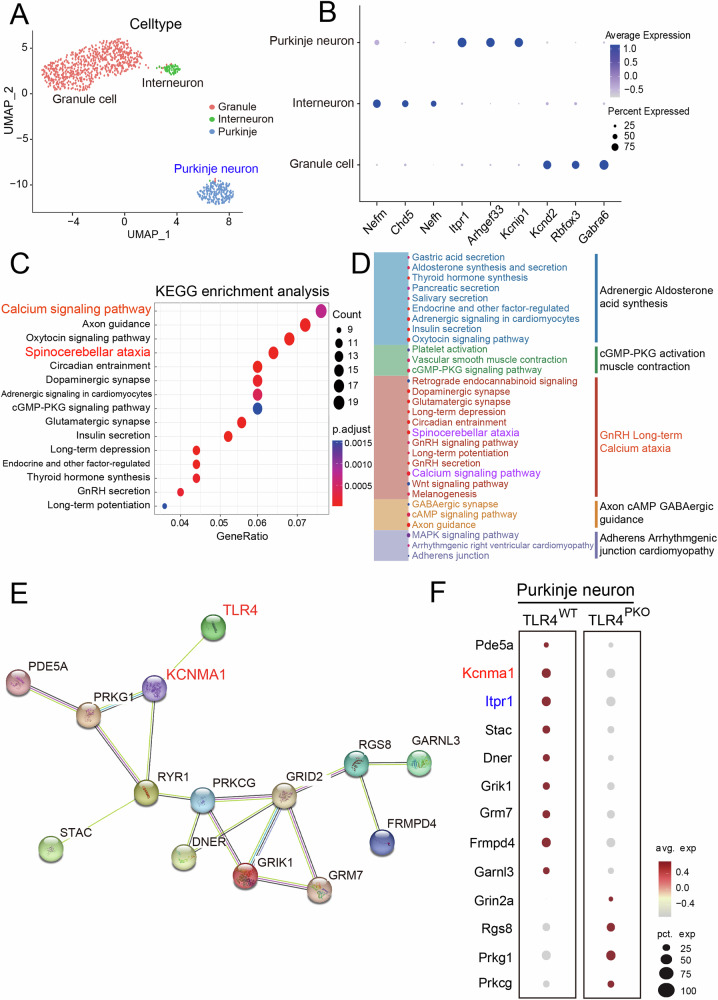
Single-cell mRNA sequencing showing calcium signaling pathway and KCNMA1 downregulation in TLR4^PKO^ cerebellum. **A** Dimension-reduced single-cell transcriptomic data visualized through Uniform Manifold Approximation and Projection (UMAP). The color code illustrates the cell type identity. **B** Bubble plots showing expression levels of interneuron markers (Nefm, Chd5, Nefh), Purkinje neuron markers (Itrpr1, Aghgef33, Kcnip1), and granule cell markers (Kcnd2, Rbfox3, Gabra6). **C** Compared to WTs, numerous KEGG calcium-associated signaling pathways were significantly downregulated in TLR4^PKO^ cerebellum. **D** Left: The top 30 most enriched KEGG pathways in TLR4^WT^ compared to TLR4^PKO^ (TLR4^WT^/TLR4^PKO^). Right: Cluster dendrogram of the pathways on the left. **E** Protein–protein interaction network associated with calcium-related differentially expressed genes. Each node represents a connected protein. Line thickness is proportional to the number of connections between communities. The full PPI network image is shown in Fig. S3. **F** Bubble plots showing expression levels of differentially expressed genes in PNs. The significance threshold was set to an adjusted *p* < 0.05 (**C**).

To identify potential molecular mechanisms underlying PN dysfunction in TLR4-deficient mice, we performed standardized scRNA-seq. Results revealed 16 distinct gene clusters corresponding to three discrete cell types, PNs (identified by Itpr1), interneurons (identified by Nefm), and granule cells (identified by Gabra6) (Fig. 5A, B). Further GSEA analysis (Fig. 5C) and the dendrogram of KEGG pathway annotations (Fig. 5D) revealed that TLR4^WT^ PNs were enriched in genes related to ‘calcium signaling pathway’ and ‘spinocerebellar ataxia pathway’. These gene expression data were then integrated with the STRING protein–protein interaction (PPI) network to identify the portion of the network spanning TLR4^PKO^ (Supplementary Fig. 3). The PPI network related to ‘calcium signaling pathway’ suggests that *KCNMA1*, encoding the calcium-activated potassium channel BK, may be a key gene regulating calcium signaling and thus strongly influencing PN function (Fig. 5E). High levels of calcium signaling pathway genes such as *Kcnma1*, *Itpr1*, *Frmpd4*, *Pde5a*, and *Grm7* were also found in the TLR4^WT^ subset compared to TLR4^PKO^ (Fig. 5F). Collectively, these results suggest that downregulation of *Kcnma1* and ensuing abnormalities in PN spiking patterns may underly PN dysfunction and ataxia [15].

### TLR4 deletion in PNs prolongs the AHP and induces irregular firing through decreased BK channel activity

**Fig. 6:**
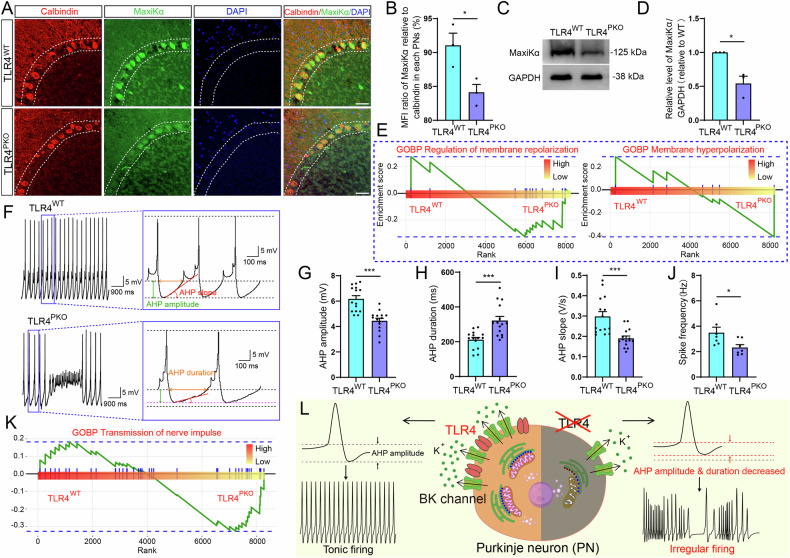
Purkinje neuron-specific TLR4 deletion reduced BK channel expression and impaired BK-mediated after-hyperpolarizations (AHPs). **A** Representative confocal images of a cerebellar sagittal section showing immunostained for the BKα subunit (Maxikα) and calbindin antibodies. **B** Immunoexpression of Maxikα was reduced relative to calbindin immunoexpression in TLR4 cerebellum as measured by mean fluorescence intensity (MFI). **C** Reduced BK expression in whole cerebellum of TLR4^PKO^ mice confirmed by westing blotting. Shown are the representative bands for Maxikα and GAPDH (gel loading control) from whole cerebellar lysate. **D** Decreased Maxikα expression was confirmed by densitometric analysis. **E** Membrane polarization-related GO Biological process pathways downregulated in TLR4^PKO^ cerebellum compared to TLR4^WT^ cerebellum. The y-axes represent the GSVA score and the x-axes represent the rank value. Compared to TLR4^WT^ cerebellum, genes related to ‘regulation of membrane repolarization’ and ‘membrane hyperpolarization’ were downregulated in TLR4^PKO^ cerebellum. **F** Representative whole-cell current-clamp recordings showing spontaneous firing of PNs in cerebellar slices from TLR4^PKO^ and TLR4^WT^ mice. The blue frame insets show expanded views of the spikes. The orange and green dotted lines mark the AHP duration and AHP amplitude, respectively. The angle between the red line and the black/purple dotted line (bottom) marks the AHP slope. Average AHP amplitude (**G**), slope (**I**), and spike frequency (**J**) were all reduced by TLR4 deletion. **H** Average AHP duration was increased by TLR4 deletion. **K** GO Biological process pathways related to ‘transmission of nerve impulse’ were downregulated in TLR4^PKO^ cerebellum. The y-axes represent the GSVA score and the x-axes represent the rank value. **L** Schematic illustration summarizing these findings. Scale bar = 50 μm (**A**). Each point in the histogram represents a mouse (**B**, **D**). Each point in the histogram represents a cell from 7 mice in the TLR4^PKO^ group, 7 mice in TLR4^WT^ group (**G**–**J**). Data are represented as mean ± SEM, *p < 0.05; ***p < 0.001. The significance threshold was also set to an adjusted *p* < 0.05 (**E**, **K**). BP biological process.

To investigate possible changes of BK channel (also termed MaxiK) channel expression and function due to TLR4 deficiency, we first performed immunohistochemical staining using a monoclonal antibody to the α subunit of the BK channel (MaxiKα). Confocal images showed that MaxiKα expression was higher in (calbindin-positive) PNs of cerebellar sagittal sections from TLR4^WT^ mice than TLR4^PKO^ mice (Fig. 6A). Moreover, the MFI of MaxiKα relative to calbindin was higher in TLR4^WT^ PNs than TLR4^PKO^ PNs (Fig. 6B). Reduced MaxiKα expression in TLR4^PKO^ cerebellum was further confirmed by western blotting (Fig. 6C) of whole cerebellar lysates (Fig. 6D).

The BK channel contributes to AP repolarization and AHPs by allowing rapid K^+^ efflux under physiological conditions [43], so we examined the top upregulated pathways related to membrane potential ranked by NES, included pathways regulating membrane repolarization and hyperpolarization (Fig. 6E). These analyses suggested possible abnormalities in membrane potential repolarization and altered spiking patterns due to TLR4 deficiency, so we recorded APs from PNs in acutely isolated cerebellar slices from TLR4^PKO^ and TLR4^WT^ mice by whole-cell current clamp. The PNs of TLR4^WT^ slices showed a typical spontaneous tonic firing pattern, while PNs of TLR4^PKO^ slices exhibited an irregular firing pattern characterized by predominant burst firing (Fig. 6F), consistent with previous studies of BK channel inhibition [27]. Next, we analyzed whether the afterpotentials of APs (AHPs) are altered by TLR4-specific knockout in PNs and found a significant reduction in AHP amplitude (Fig. 6F, G), prolongation of AHP duration (Fig. 6F, H), shallower slope for decay (Fig. 6F, I), and decreased spike frequency (Fig. 6F, J). Consistent with impaired AHP, bioinformatics analysis indicated reduced enrichment of the ‘transmission of nerve impulse’ pathway according to NES in TLR4^PKO^ PNs (Fig. 6K). To clarify whether the irregular firing pattern in PNs of TLR4^PKO^ slices was due to BK channel inhibition, we used BMS-191011, a kind of BK channel opener [44, 45], to treat TLR4^PKO^ slices, and found that BMS-191011 could rescue the kinetics of BK-mediated AHPs (Supplementary Figure 4). Together, these results suggest that TLR4 deficiency alters the kinetics of BK-mediated AHPs and the firing patterns of PNs (Fig. 6L).

### TLR4 deletion in PNs decreased cytoCa^2+^ and impaired mitochondria

**Fig. 7:**
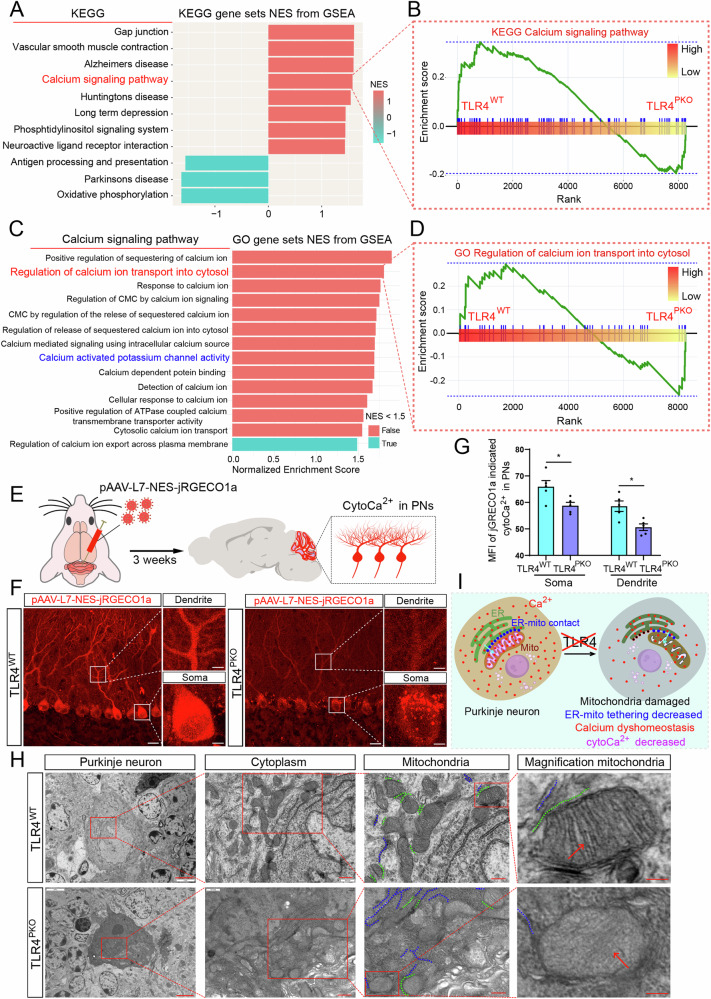
PN-specific deletion of TLR4 reduced cytosolic calcium concentration (cytoCa^2+^), damaged mitochondria, and disrupted associations between mitochondria and endoplasmic reticulum (ER). **A** Top KEGG pathways downregulated in TLR4^PKO^ cerebellum. **B** GSEA visualization of calcium signaling pathways. **C** The top calcium signaling-related GO pathways downregulated in TLR4^PKO^ cerebellum. **D** GSEA visualization of ‘regulation of calcium ion transport into cytosol’. **E** Schematic illustration showing injection of a pAAV encoding the cytoCa^2+^ indicator jRGECO1a into the cerebellar vermis. **F** Representative confocal images of pAAV-L7-NES-jRGECO1a labeling, indicative of cytoCa^2+^, in PNs of a cerebellar sagittal section. The magnification images show the cytoCa^2+^ signal in soma and dendrite. **G** Decreased MFI of jRGECO1a indicating reduced cytoCa^2+^ in soma and dendrites of TLR4^PKO^ PNs. Each point in the histogram represents a mouse. **H** Representative transmission electron images of PNs in four-month-old TLR4^PKO^ cerebellum showing somal shrinkage, increased cytoplasmic density, increased electron density, nucleolar fragmentation, nucleoplasmic concentration, mitochondrial swelling, and degeneration of mitochondrial double membranes and cristae (red arrow). Mitochondria in TLR4^PKO^ PNs also showed weakened ER–mitochondria tethering (dotted green lines indicate ER in contact with mitochondria, dotted blue lines indicate ER not in contact with mitochondria). **I** Schematic illustration of these results. Scale bar = 20 μm in lower magnification and 5 μm in higher magnification images (**F**), 5 μm, 500 nm, 250 nm, 100 nm in order of increasing magnification images (**H**). The significance threshold was set to an adjusted *p* < 0.05 (**A**–**D**). CMC cardiac muscle contraction (in **C**); ER endoplasmic reticulum, Mito mitochondria.

The AP firing pattern of PNs is strongly dependent on BK activity and thus on the cytoplasmic Ca^2+^ concentration (cytoCa^2+^). CytoCa^2+^ in turn reflects the balance between Ca^2+^ influx from the extracellular space, mainly induced by APs, and various Ca^2+^ efflux and sequestration pathways. GSEA analysis showed that many neurological disease-related pathways and neurophysiological processes were downregulated in TLR4^PKO^ PNs (Fig. 7A). Of these, the extent of downregulation was greater for ‘calcium signaling pathway’ than for ‘Alzheimer’s disease’, ‘Huntington’s disease’, ‘long-term depression’, and ‘neuroactive ligand receptor interaction’ among others (Fig. 7B, Supplementary Fig. 5A–D). Further, many calcium regulation-related processes were downregulated in TLR4^PKO^ PNs (Fig. 7C), especially ‘regulation of calcium ion transport into cytosol’ (Fig. 7D). To investigate whether impaired firing by TLR4^PKO^ PNs is related to abnormal cytoCa^2+^, we injected the L7 promotor-guided adeno-associated virus carrying a genetically encoded cytoCa^2+^ indicator (pAAV-L7-NES-jRGECO1a) (Fig. 7E) into the cerebellar vermis and examined fluorescence signals in slices 3 weeks later (Fig. 7F). The MFI of cytoCa^2+^ was significantly weaker in both the soma and dendrites of TLR4^PKO^ PNs than TLR4^WT^ PNs (Fig. 7F, G). This reduced cytoCa^2+^ may explain the abnormal AHP, although not the abnormal reduction in the PN population or dendritic morphology.

A loss-of-function BK channel mutation (BK_G354S_) was found to cause mitochondrial impairment and progressive cerebellar ataxia in patients [26], so we examined if mitochondria are impaired by PN-specific TLR4 knockout by transmission electron microscopy. As shown in Fig. 7H and Supplementary Fig. 6, mitochondria in TLR4^PKO^ PNs appeared swollen, with disrupted double membranes and cristae. Further, some TLR4^PKO^ PNs showed somal shrinkage, increased cytoplasmic density, increased electron density, nucleolar fragmentation, and nucleoplasmic concentration compared to TLR4^WT^ PNs. In addition, we found weakened endoplasmic reticulum (ER)–mitochondria contact in TLR4^PKO^ PNs (dotted green lines in Fig. 7H and Supplementary Fig. 6), suggesting that PN-specific TLR4 deletion may lead to weaker ER–mitochondria tethering, which is critical for ER–mitochondria Ca^2+^ transfer and downstream processes such as Ca^2+^ buffering and metabolic regulation [46]. Reduced ER–mitochondria tethering was also suggested by scRNA-seq analysis (Supplementary Fig. 5E, F).

Reduced ER–mitochondria tethering is thought to impair neuronal mitochondrial homeostasis [47] and energy production, potentially resulting in neuronal degeneration [48, 49]. Thus, weakening of ER–mitochondria tethering associated with TLR4 knockout may explain the mitochondria damage in PNs as well as the reduced number of PNs, the diminished arborization of PN dendrites, and the lower spine density on PN dendrites in TLR4^PKO^ mouse cerebellum, ultimately resulting in abnormal PN firing and cerebellar ataxia-like behaviors.

## Discussion

**Fig. 8:**
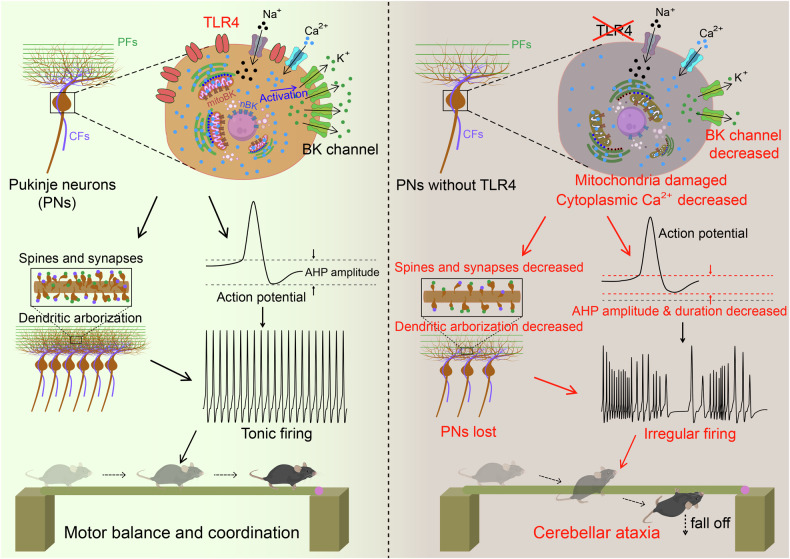
Schematic illustration showing that TLR4 deficiency in PNs drives cerebellar ataxia by impairing cytoCa^2+^ homeostasis, mitochondrial function, BK channel-mediated AHPs, dendrite development, circuit formation, and normal spiking activity patterns.

We present evidence that TLR4 signaling is essential for proper Purkinje neuron function and cerebellar motor control. While TLR4 has been implicated in movement disorders such as Parkinson’s disease [50] and amyotrophic lateral sclerosis [51], pathogenic effects were associated with neuroinflammation through activation of microglia. In this study, we demonstrate the involvement of TLR4 in non-immune pathway(s) that protect against cerebellar ataxia by maintaining the normal functions of PNs. We found that PN-specific TLR4 deletion results in the downregulation of BK channel activity, leading to aberrant PN firing and ultimately in motor control deficits. However, BK channel downregulation may be an upstream pathogenic event as TLR4 knockout was also associated with lower cytoCa^2+^, mitochondrial damage, loss of synaptic inputs from CFs and PFs, diminished dendritic arborization, and impaired spine development, changes that may lead to loss of PN function, degeneration, and ultimately to cerebellar ataxia (Fig. 8). Additional research is required to elucidate the precise pathogenic pathway from TLR4 insufficiency to ataxia.

Dysfunction of Purkinje neuron BK channels is believed to be an important factor underlying many forms of cerebellar ataxia as these channels contribute to K^+^ outflow during AP repolarization and help shape the output firing patterns critical for proper motor control. Indeed, PNs from ataxic BK knockout mice showed reduced AHP amplitude and PN firing frequency [25], while BK channel inhibition substantially altered the normal firing pattern [27]. Further, BK channel activators have been reported to restore the AHP, normalize firing patterns, and improving motor dysfunction [52]. Consistent with these findings, PN-specific TLR4 deletion reduced AHP amplitude, prolonged AHP duration, altered the normal spontaneous AP firing pattern, and induced multiple ataxic behaviors. What’s more, the BK channel opener could restore the impaired AHP in PNs of TLR4^PKO^ mice. TLR4 binds the highly pro-inflammatory bacterial toxin LPS, and LPS has been shown to induce BK current activation in microglia [28] and bladder umbrella cells [53], leading to the release of inflammatory cytokines. Thus, TLR4 may serve as a BK activator in multiple signaling pathways and cell types, both immune-related and non-immune related. Consistent with this notion, the present study suggests that TLR4 regulates PN firing by influencing the BK channel-mediated AHP in healthy cerebellar tissue.

Activation of the BK channel is facilitated by submembrane elevation of intracellular calcium. Intracellular calcium dysregulation in PNs has been strongly implicated in abnormal PN pace-making activity [54] and cerebellar ataxia [55–58]. We found that TLR4 knockout reduced intracellular calcium concentration in PNs. While the underlying mechanism is still unclear, we also show that TLR4 deletion impairs mitochondrial structural integrity, which may in turn alters intracellular Ca^2+^ homeostasis. However, the main Ca^2+^ source for BK channel activation is influx through voltage-gated Ca^2+^ channels (VGCCs), so further study is required to identify possible signaling pathways between TLR4 and VGCCs. The other major source of cytoCa^2+^ is release from the ER, which is regulated mainly by the type 1 inositol 1,4,5-trisphosphate receptor (IP3R1, also termed Itpr1) [59]. Several studies have indicated that IP3R1 mutation or deficiency in PNs can dysregulate intracellular Ca^2+^ homeostasis and lead to motor deficits [60, 61]. Consistent with these findings, Itpr1 mRNA expression was reduced in TLR4^PKO^ PNs (Fig. 5F), suggesting that diminished ER Ca^2+^ release contributed to the lower cytoCa^2+^ level.

BK channels are also present in the inner mitochondrial membrane [62] and in the inner nuclear membrane [63], where they are known as mitoBK and nBK channels, respectively. The mitoBK channel is thought to protect mitochondria from calcium overload by reducing the inner membrane potential, the main driving force for Ca^2+^ entry [64], and by facilitating mitochondrial permeability transition pore (mPTP)-mediated Ca^2+^ release [62].

Therefore, the decreased cytoCa^2+^ and impaired mitochondria found in TLR4^PKO^ PNs may result from reduction of mitoBK channel expression. At the same time, weakening of ER–mitochondria tethering in PNs may also promote calcium dyshomeostasis and mitochondrial dysfunction. In addition, nBK channels have been shown to regulate neuronal development, plasticity, long-term memory formation, and cell survival by activating genes required for synapse formation, dendritic elaboration, and synaptic plasticity [65, 66]. The BK blocker paxilline was found to induce a transient Ca^2+^ increase in the nucleoplasm of neurons, which was abolished by BKα subunit knockout [63], suggesting that impaired nBK channel activity can cause nucleoplasmic Ca^2+^ dyshomeostasis and ultimately disrupt gene expression programs necessary for maintaining normal neuronal morphology and function. Therefore, the abnormal PN firing patterns and loss of PF–PN and CF–PN synapses in TLR4^PKO^ mice may be related to nucleoplasmic Ca^2+^ dyshomeostasis caused by reduced nBK channel expression. These changes could in turn be self-sustaining as dendritic complexity is highly dependent on neuronal input and vice versa [67]. However, blocking nBK channel expression using BKα-specific shRNAs was previously found to increase dendritic arborization through a nuclear Ca^2+^ signaling-dependent pathway [63], so the decreased dendritic arborization observed in TLR4^PKO^ PNs may be due to other factors.

A reduction in dendritic arborization and morphological complexity is a common feature of many neurological conditions, such as cerebellar ataxia and Alzheimer’s disease as well as aging [68–71]. In recent years, there has been growing interest in the contributions of PN calcium dyshomeostasis and mitochondrial dysfunction to cerebellar ataxia. Activation of the NAD^+^-dependent deacetylase sirtuin 1 was reported to rescue PNs from degeneration in spinocerebellar ataxia type 7 (SCA 7) model mice by restoring normal BK channel activity, calcium homeostasis, and regular PN firing pattern [72]. Alternatively, downregulation of INPP5A, a terminator of inositol 1,4,5-triphosphate (IP_3_) signaling, was found to reduce intracellular calcium and promote PN degeneration in SCA 17 [73]. Activation of LINE-1 was also found to induce PN degeneration and cerebellar ataxia by increasing ER stress and potentially disturbing calcium homeostasis [74]. Mice with PN-specific Mfn2 knockout exhibited dysfunctional mitochondrial oxidative phosphorylation, progressive PN degeneration, and cerebellar ataxia [75]. Collectively, these findings suggest that the abnormal cytoCa^2+^ caused by TLR4 deletion may underly the observed PN damage revealed by transmission electron microscopy (Fig. 7H). These findings of reduced dendritic arborization, synaptic input, spine density, and AHP amplitude as well as loss of mitochondrial structural integrity and weakened ER–mitochondria tethering are signs of “functional neurodegeneration” rather than PN loss [76]. Thus, ataxia may result from both PN loss and stable dysfunction.

A growing body of evidence suggests that TLR4 may regulate various neuronal functions through non-immune pathways. Based on the results of our previous study [6], the current study investigated the contributions of non-immune pathways involving TLR4 and revealed previously unknown functions in maintaining normal PN activity and processing for motor control. However, the present study has several limitations. First, we have no direct evidence for reduced mitoBK and nBK channel activities, abnormal ER Ca^2+^ release, disrupted ER–mitochondria Ca^2+^ exchange, or metabolic dysfunction caused by weakened ER–mitochondria tethering. Second, the specific causal associations among these many pathological signs are still unverified. Nonetheless, these findings add to a growing body of evidence that TLR4 influences a multitude of physiological and pathological processes through non-immune pathways, including the PN activity required for normal motor control.

### Supplementary information

supplementary information

supplemental video 1

supplemental video 2

supplemental video 3

supplemental data S1

original western blot

## Data Availability

All data needed to evaluate the conclusions in the paper are present in the paper and/or the Supplementary Materials.
