# Supplementary material for: Toll-like receptor 4 deficiency in Purkinje neurons drives cerebellar ataxia by impairing the BK channel-mediated after-hyperpolarization and cytosolic calcium homeostasis
Source: Cell Death Dis. 2024 Aug 15;15(8):594. doi: 10.1038/s41419-024-06988-w (PMC11327311; doi:10.1038/s41419-024-06988-w)
Supplement: Supplementary file 1 — supplementary information [file 41419_2024_6988_MOESM1_ESM.docx]

**Toll-like receptor 4 deficiency in Purkinje neurons drives cerebellar ataxia by impairing the BK channel-mediated after-hyperpolarization and cytosolic calcium homeostasis.**

Jianwei Zhu^1#^, Wenqiao Qiu^1#^, Fan Wei^1,2^, Jin Zhang^1^, Ying Yuan^1^, Ling Liu^1^, Meixiong Cheng^1^, Huan Xiong^1*^, Ruxiang Xu^1*^

^1^ Department of Neurosurgery, Sichuan Provincial People’s Hospital, School of Medicine, University of Electronic Science and Technology of China, Chengdu, 610072, China.

^2^ Department of Critical Care Medicine, Mianyang Orthopaedic Hospital, Mianyang, Sichuan Province, 621000, China.

^#^ These authors contributed equally to this work.

* Corresponding authors:

Ruxiang Xu, Email: [xuruxiang1123@uestc.edu.cn](mailto:xuruxiang1123@uestc.edu.cn);

Huan Xiong, Email: [huan_xiong@163.com](mailto:huan_xiong@163.com)

**Supplementary figures**

**
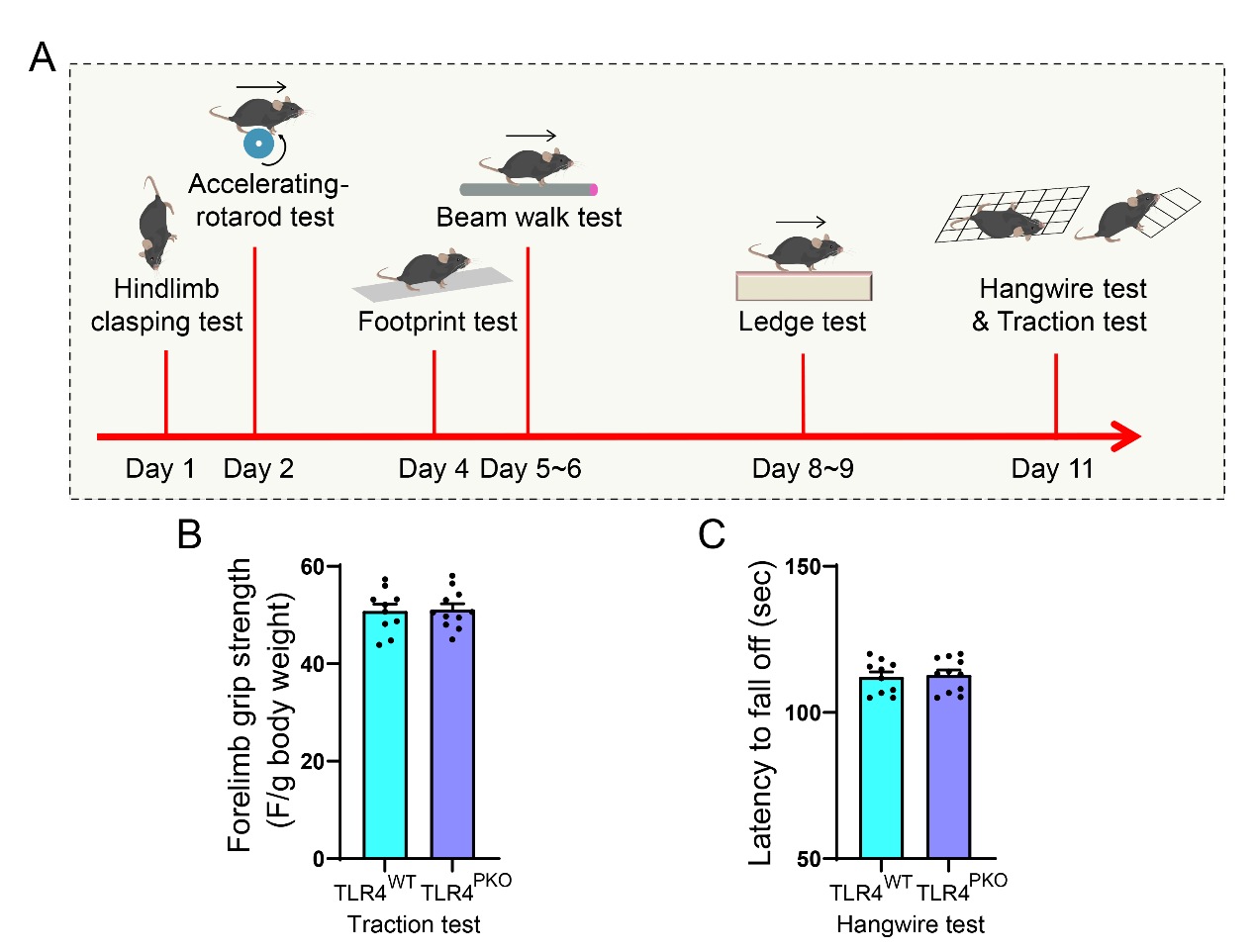
**

**Figure S1.** (A) Experimental schedule for cerebellar ataxia-related behavioral testing. (B, C) Skeletal muscle strength assess by traction test (B) and hangwire test (C).


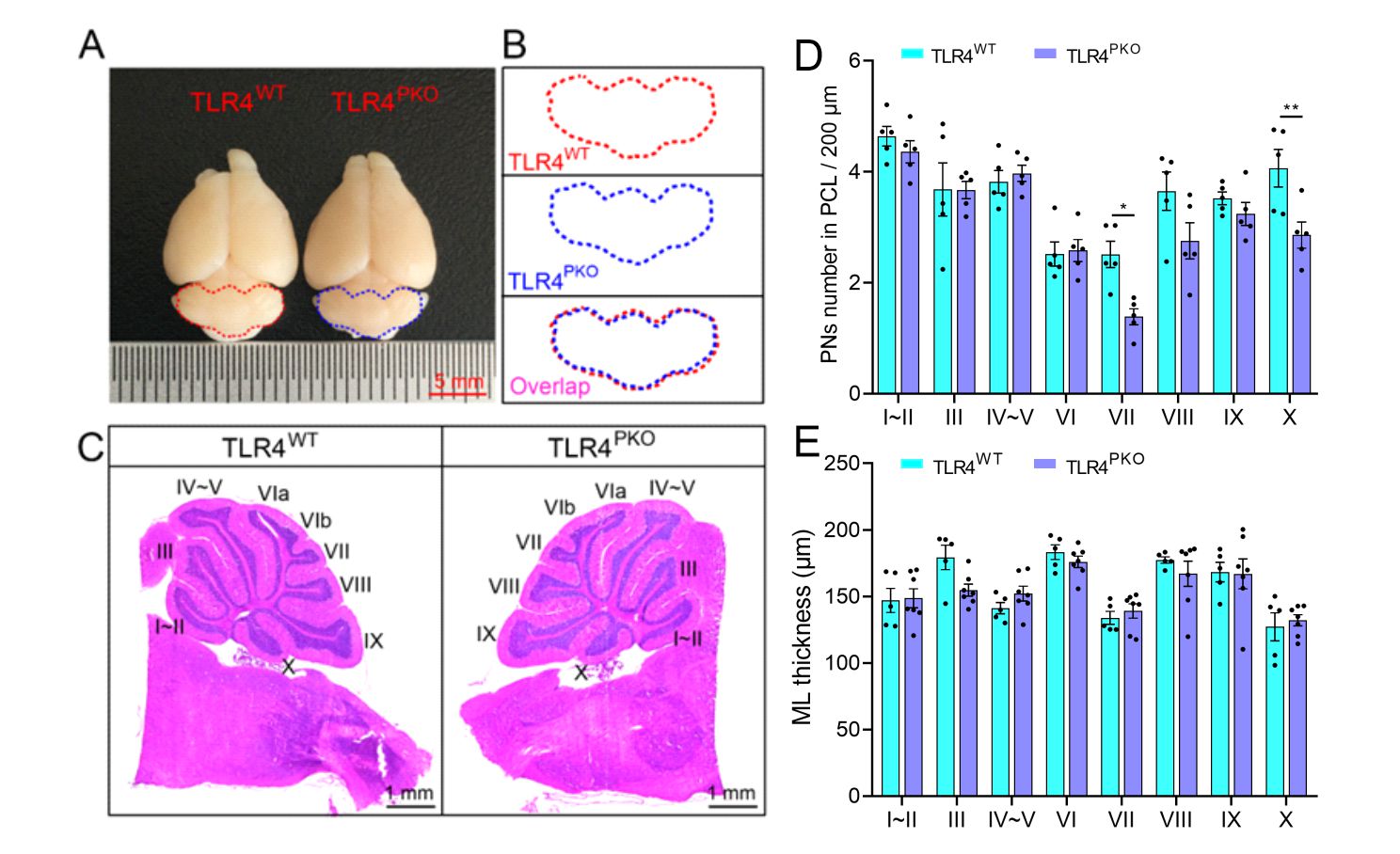


**Figure S2.** Purkinje neuron (PN)-specific TLR4 deletion did not alter gross cerebellar morphology. (A) Representative macroscopic images of whole brain from TLR4^WT^ and TLR4^PKO^ mice. The cerebellum is outlined by dotted lines. (B) Cerebellar outlines are roughly comparable between genotypes. (C) Representative images of H&E-stained sagittal sections from TLR4^WT^ and TLR4^PKO^ cerebellum showing similar lamination and foliation patterns. (D) Quantitative analysis showing PNs number in PCL per 200μm in different lobules of TLR4^WT^ and TLR4^PKO^ mice. (E) Quantitative analysis showing ML thickness in different lobules of TLR4^WT^ and TLR4^PKO^ mice. Scale bar = 5 mm (A); 1 mm (C). Data are represented as mean ± SEM, * p < 0.05; ** p < 0.01. ML, molecular layer; PCL, Purkinje cell layer (D, E).

**
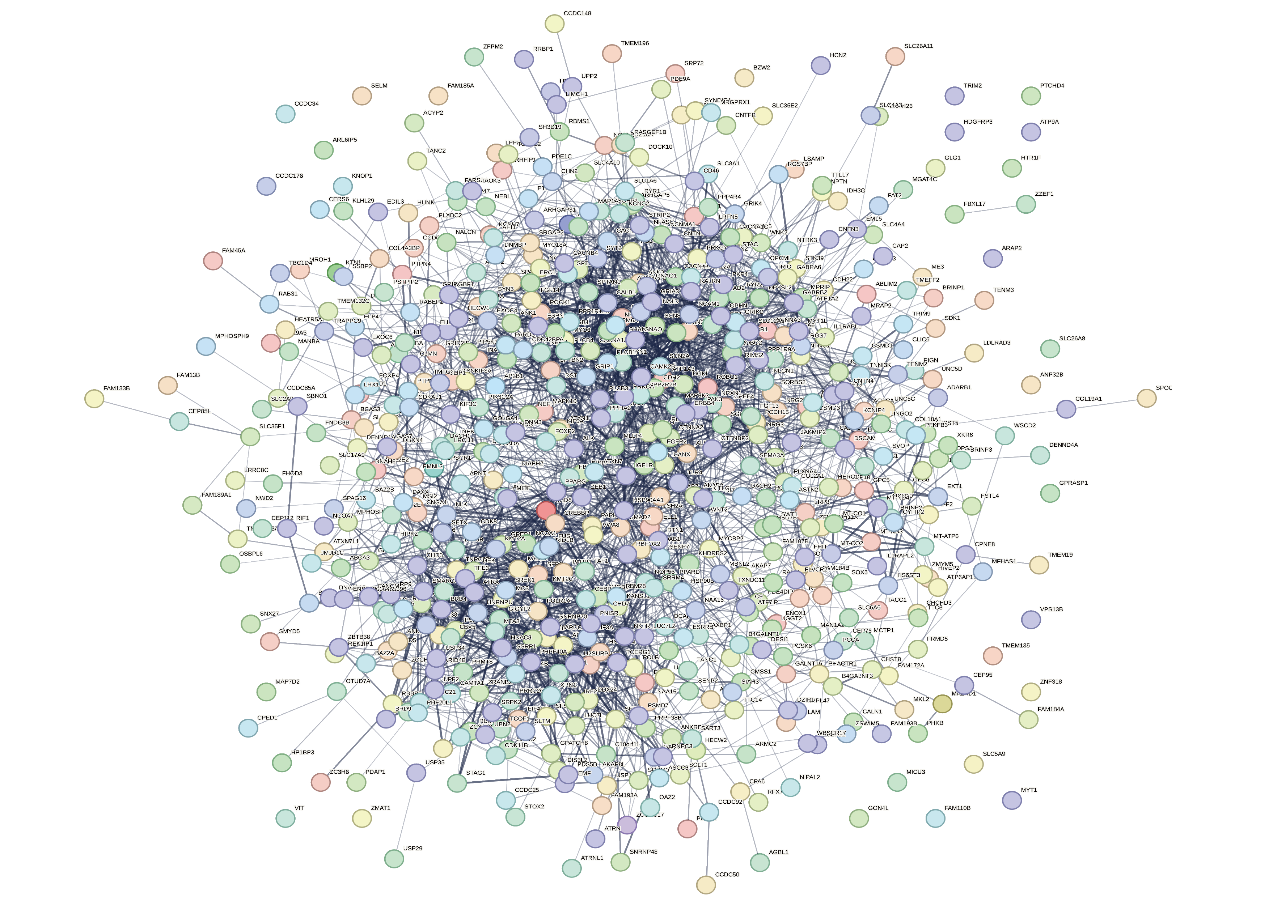
**

**Figure S3**. A protein–protein interaction network for genes differentially expressed between TLR4^WT^ and TLR4^PKO^ mice. Each node represents a connected protein. Line thickness is proportional to the number of connections between communities.


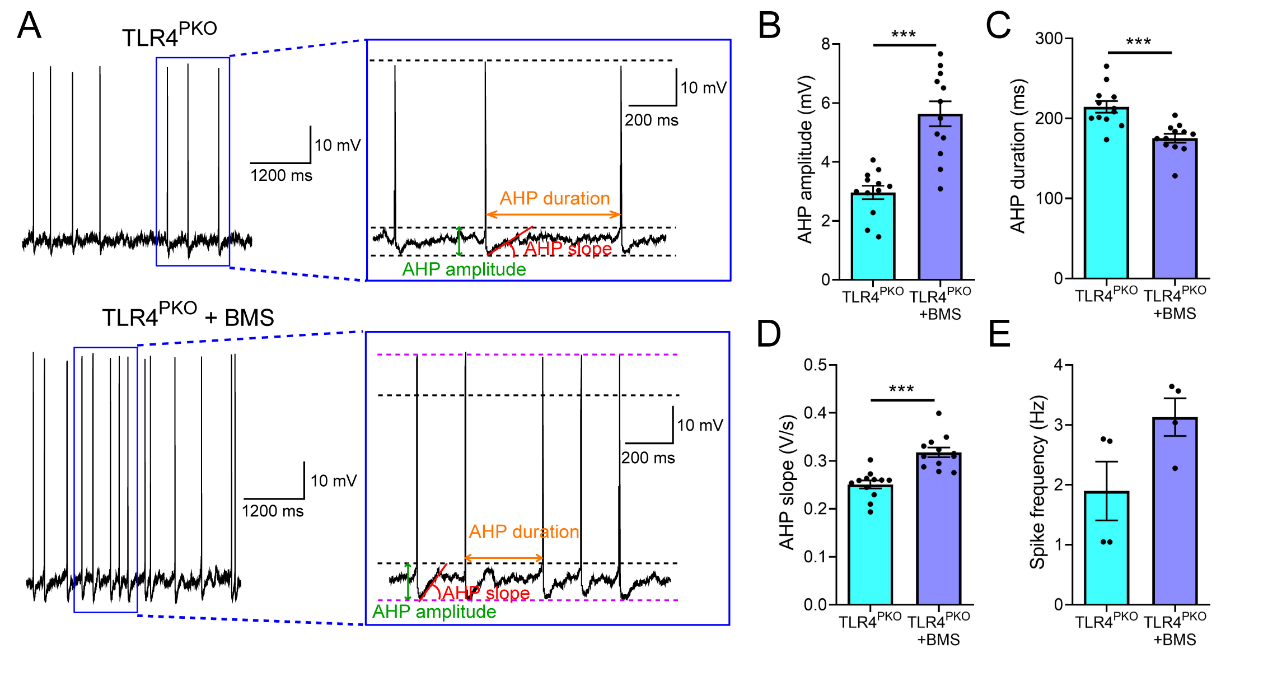


**Figure S4**. BK channel opener BMS-191011 (BMS) rescued the impaired BK-mediated after-hyperpolarizations (AHPs) in TLR4^PKO^ mice. (A) Representative whole-cell current-clamp recordings showing spontaneous firing of PNs in cerebellar slices from TLR4^PKO^ mice perfused with aCSF or aCSF containing BSM. The blue frame insets show expanded views of the spikes. The orange and green dotted lines mark the AHP duration and AHP amplitude, respectively. The angle between the red line and the black/purple dotted line (bottom) marks the AHP slope. (B-D) Average AHP amplitude (B), and slope (D) were all increased, while average AHP duration (C) was reduced, by BMS incubation. (E) Spike frequency was not affected by BMS incubation. Each point in the histogram represents a cell from 4 mice in the TLR4^PKO^ group (B-E). Data are represented as mean ± SEM, *** *p* < 0.001.

**
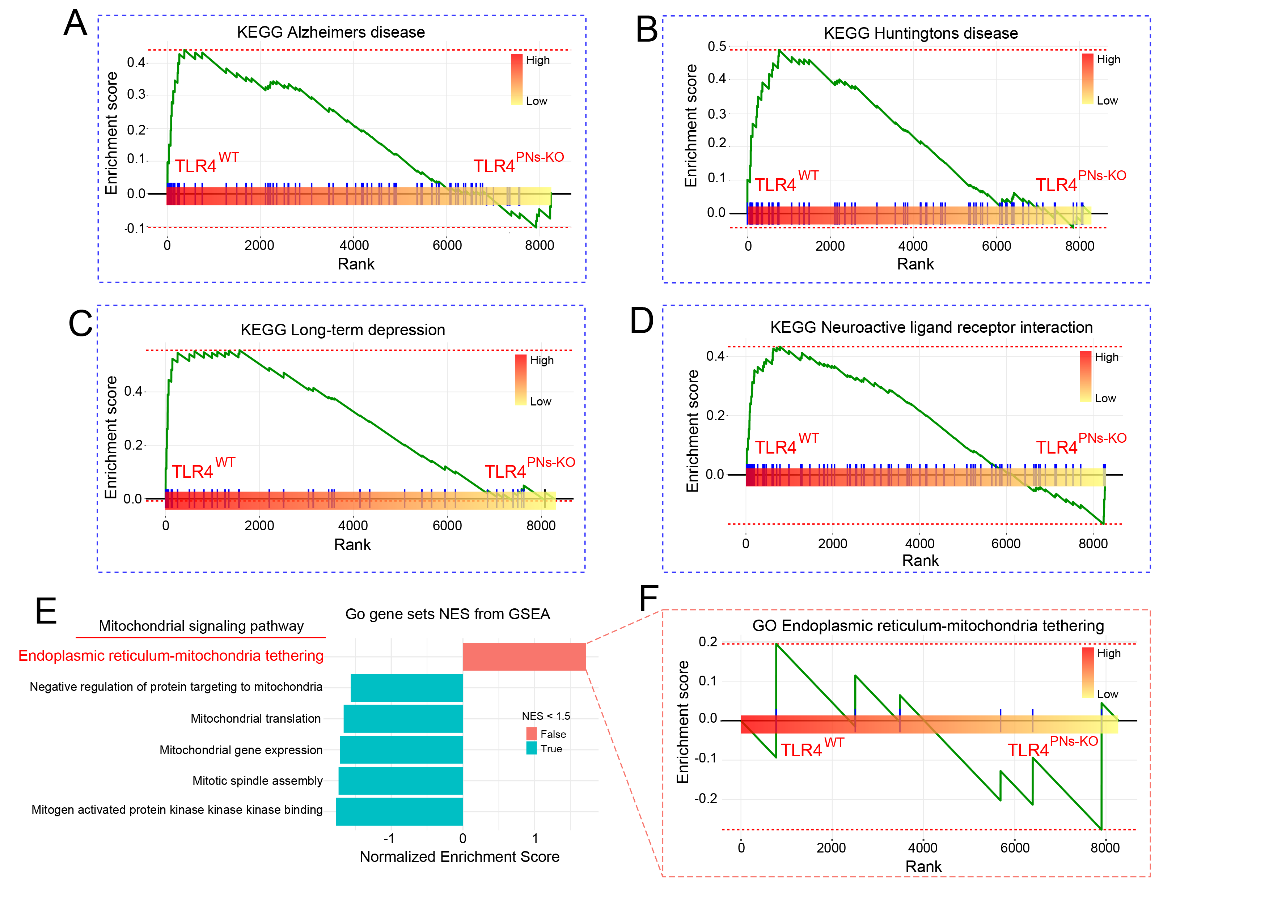
**

**Figure S5**. Differentially expressed genes are enriched in neurological disease-related genes and genes associated with various neurocellular process pathways. (A-D) GSEA visualization of the Alzheimer’s disease pathway (A), Huntington’s disease pathway (B), long-term depression pathway (C), and neuroactive ligand receptor interaction pathway (D). (E) The top GO mitochondrial signaling pathways downregulated in TLR4^PKO^ cerebellum compared to TLR4^WT^ cerebellum. (F) GSEA visualization of the endoplasmic reticulum–mitochondria tethering pathway. The significance threshold was set to an adjusted *p* < 0.05 (A- E).


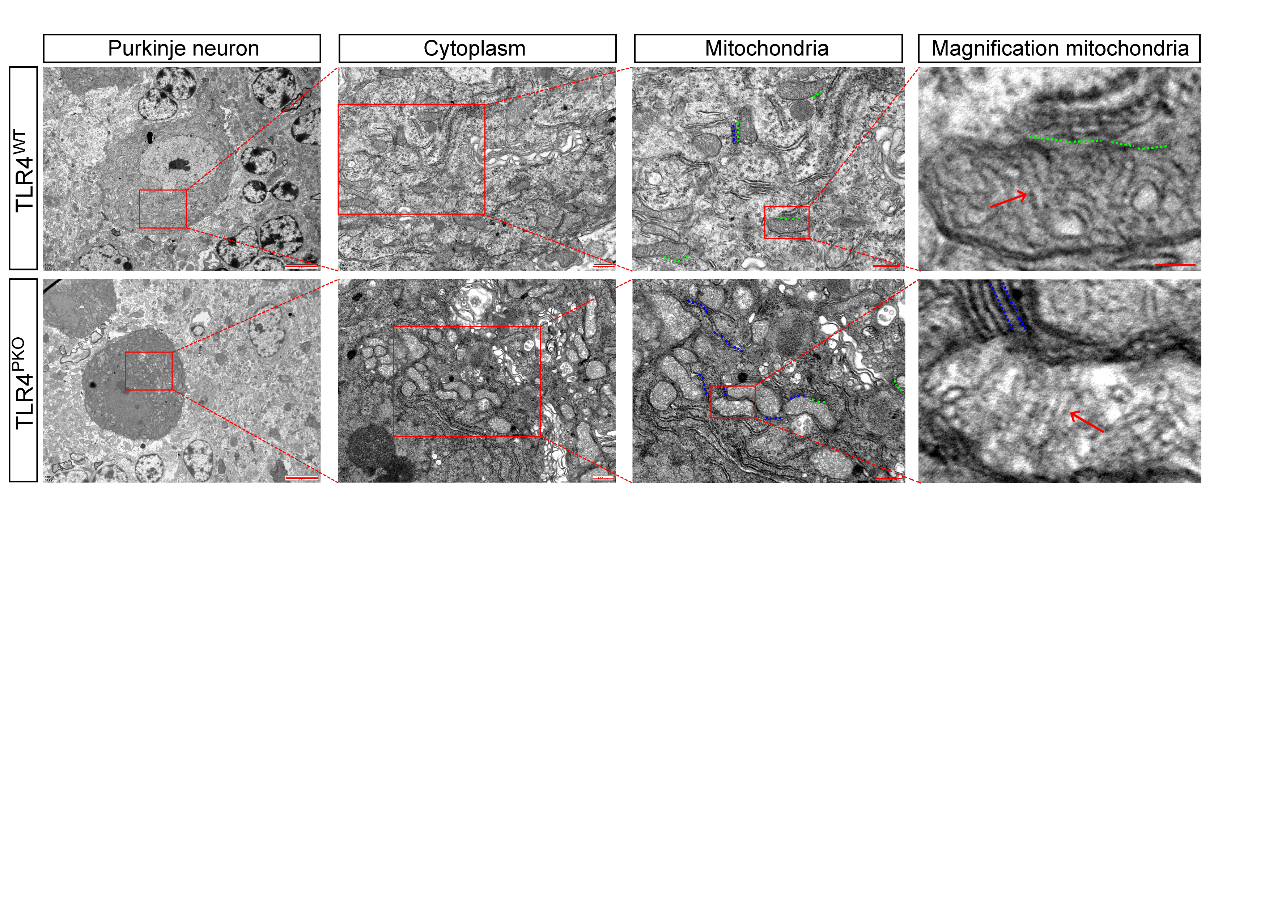


**Figure S6**. Representative transmission electron images of PNs in one-month-old TLR4^PKO^ cerebellum showing somal shrinkage, increased cytoplasmic density, increased electron density, nucleolar fragmentation, nucleoplasmic concentration, mitochondrial swelling, and degeneration of mitochondrial double membranes and cristae (red arrow). Mitochondria in TLR4^PKO^ PNs also showed weakened ER–mitochondria tethering (dotted green lines indicate ER in contact with mitochondria, dotted blue lines indicate ER not in contact with mitochondria). Scale bar = 5 μm, 500 nm, 250 nm, 100 nm in order of increasing magnification images. ER, endoplasmic reticulum.

**Table S1.** Antibodies information

| Antibody | Company | | Cat. No. | | Species | | Dilution | | Application | |
| --- | --- | --- | --- | --- | --- | --- | --- | --- | --- | --- |
| Calbindin | | Swant | | CB38 | | Rabbit | | 1:1000 | | IHC |
| Calbindin | | Swant | | 300 | | Mouse | | 1:1000 | | IHC |
| TLR4 | | Santa Cruz | | sc-293072 | | Mouse | | 1:200 | | IHC |
| MaxiKα | | Santa Cruz | | sc-374142 | | Mouse | | 1:200 | | IHC&WB |
| vGluT1 | | Synaptic Systems | | 135302 | | Rabbit | | 1:1000 | | IHC |
| vGluT2 | | Synaptic Systems | | 135403 | | Rabbit | | 1:1000 | | IHC |
| GAPDH | | CST | | 2118 | | Rabbit | | 1:1000 | | WB |
| Alexa Fluor 488 | | ThermoFisher | | A21202 | | Anti-mouse | | 1:1000 | | IHC |
| Alexa Fluor 555 | | ThermoFisher | | A31572 | | Anti-rabbit | | 1:1000 | | IHC |
| FluoTag-X2 488 | | Abnova | | RAB00881 | | Anti-mouse | | 1:500 | | IHC |
| m-IgG_2b_ BP | | Santa Cruz | | sc-542741 | | - | | 1:1000 | | WB |
| Goat-anti Rabbit | | Signalway | | L3012 | | - | | 1:5000 | | WB |

**Videos legends**

**Video S1.** Motor performance during the accelerating-rotarod test. TLR4^PKO^ mice (lane 2~4) fell off the rod at rotation speeds easily managed by TLR4^WT^ mice (lane 1).

**Video S2.** Motor performance during the beam-walking test. TLR4^PKO^ mice (right) walked slowly and displayed frequent limb slips compared to TLR4^WT^ mice (left). TLR4^PKO^ mice frequently clasped the beam with their hindlimbs while walking forward.

**Video S3.** Motor performance during the ledge test. TLR4^PKO^ mice (right) walked slowly and had difficulty placing their hind paws on the ledge surface, unlike TLR4^WT^ mice (left).

**Data legend**

**Data S1.** The detailed analysis results of GO Biological Processes.
