# Supplementary material for: Toll-like receptor 4 deficiency in Purkinje neurons drives cerebellar ataxia by impairing the BK channel-mediated after-hyperpolarization and cytosolic calcium homeostasis
Source: Cell Death Dis. 2024 Aug 15;15(8):594. doi: 10.1038/s41419-024-06988-w (PMC11327311; doi:10.1038/s41419-024-06988-w)
Supplement: Supplementary file 5 — supplemental data S1 [file 41419_2024_6988_MOESM5_ESM.pdf]

ID	Description	GeneRatio	BgRatio	pvalue	p.adjust	qvalue	geneID	Count
GO:0050808	synapse organization	65/652	494/28814	1.27E-30	4.98E-27	3.75E-27	Nrg2/Kalrn/Syndig1/Il1rapl1/Sdk1/Ppfia2/Negr1/Mdga2/Nfasc/Gphn/Shank1/Ppp1r9a/Lingo2/Lrrc4c/Zfp804a/Lrfn5/Gpm6a/Sorbs2/Clstn3/Nrg1/Dock10/Nlgn1/Erc1/Chd4/Dnm3/Ntng1/Tanc2/Snx27/Setd5/Malat1/Tanc1/Clstn2/Il1rapl2/Sez6l/Gripap1/Cttnbp2/Pclo/Gpc6/Syn1/Sptbn2/Nptn/Prkca/Pak3/Ntrk3/Nrxn1/Myo6/Mapt/Afdn/Lrp8/Kif1a/Insr/Igf1r/Grin2a/Gabrb2/Fgfr2/Fgf13/Erbb4/Epha5/Cdh2/Ctnna2/Camk2b/Cacng2/Cacnb4/Cacnb2/Ank3	65
GO:0016358	dendrite development	41/652	317/28814	2.10E-19	4.12E-16	3.10E-16	Kalrn/Il1rapl1/Sdk1/Hecw2/Ppfia2/Fstl4/Shank1/Ppp1r9a/Arid1b/Csmd3/Phactr1/Nrg1/Dock10/Nlgn1/Dnm3/Hecw1/Rbfox2/Tanc2/Grip1/Rere/Spire1/Camsap2/Slc12a5/Clip1/Sgk1/Sema3a/Robo1/Prkg1/Pak3/Myo6/Map1a/Afdn/Lrp8/Kif1a/Srgap2/Epha5/Dscam/Crebbp/Ctnna2/Camk2b/Alk	41
GO:0042391	regulation of membrane potential	46/652	469/28814	8.31E-17	9.05E-14	6.81E-14	Akap7/Nalcn/Dgki/Kcnc2/Shank1/Ppp1r9a/Atp1a3/Ctnna3/Nlgn1/Kcnh7/Rims2/Scn2a/Akap9/Tmem135/Arl6ip5/Slc4a4/Rgs7bp/Bin1/Pclo/Rgs7/Slc8a1/Slc1a6/Scn8a/Atxn1/Ryr2/Ntrk3/Nrxn1/Mapt/Afdn/Kcnma1/Hcn2/Grin2a/Grik2/Gnaq/Gabrb2/Gabra6/Fgf14/Fgf13/Cnga1/Cacng2/Cacnb4/Cacnb2/Cacna1g/Cacna1d/Ank3/Parp1	46
GO:0007416	synapse assembly	30/652	193/28814	9.22E-17	9.05E-14	6.81E-14	Nrg2/Syndig1/Il1rapl1/Sdk1/Negr1/Mdga2/Ppp1r9a/Lingo2/Lrfn5/Gpm6a/Clstn3/Nrg1/Nlgn1/Dnm3/Setd5/Clstn2/Il1rapl2/Pclo/Gpc6/Sptbn2/Nptn/Prkca/Ntrk3/Nrxn1/Myo6/Mapt/Gabrb2/Fgf13/Erbb4/Cdh2	30

GO:0050807	regulation of synapse organization	34/652	263/28814	2.67E-16	2.10E-13	1.58E-13	Nrg2/Kalrn/Syndig1/Il1rapl1/Ppfia2/Negr1/Mdga2/Ppp1r9a/Lingo2/Zfp804a/Lrfn5/Gpm6a/Clstn3/Nlgn1/Dnm3/Tanc2/Setd5/Malat1/Tanc1/Clstn2/Il1rapl2/Gripap1/Cttnbp2/Gpc6/Prkca/Pak3/Ntrk3/Nrxn1/Afdn/Lrp8/Kif1a/Cdh2/Ctnna2/Camk2b	34
GO:0007409	axonogenesis	46/652	493/28814	5.59E-16	3.28E-13	2.47E-13	Kalrn/Nefh/Fstl4/Cntn4/Nfasc/Robo2/Plxna4/Lrrc4c/Plppr4/Trio/Nrg1/Unc5d/Adarb1/Mycbp2/Ntng1/Sptbn4/Golga4/Tenm2/Unc5c/Sema3a/Nptn/Robo1/Prkca/Pak3/Ntrk3/Nefm/Ncam1/Mapt/Map1a/Lhx1/Kif5a/Igf1r/Hsp90aa1/Hsp90ab1/Fgfr2/Fgf13/Ext1/Epha5/Dst/Dscam/Dcc/Dpysl2/Cntn1/Cdh2/Ctnna2/Ank3	46
GO:0099003	vesicle-mediated transport in synapse	33/652	254/28814	6.35E-16	3.28E-13	2.47E-13	Syndig1/Ppfia2/Cadps2/Dgki/Dennd1a/Nrg1/Nlgn1/Rims2/Erc1/Dnm3/Trim9/Grip1/Dnajc6/Ap2b1/Snap47/Gripap1/Bin1/Syn3/Pclo/Mapk10/Syt2/Syt1/Syn1/Sptbn2/Itsn2/Rock1/Prkca/Nrxn1/Myo6/Fgf14/Cdh2/Canx/Cacnb4	33
GO:0050803	regulation of synapse structure or activity	34/652	271/28814	6.68E-16	3.28E-13	2.47E-13	Nrg2/Kalrn/Syndig1/Il1rapl1/Ppfia2/Negr1/Mdga2/Ppp1r9a/Lingo2/Zfp804a/Lrfn5/Gpm6a/Clstn3/Nlgn1/Dnm3/Tanc2/Setd5/Malat1/Tanc1/Clstn2/Il1rapl2/Gripap1/Cttnbp2/Gpc6/Prkca/Pak3/Ntrk3/Nrxn1/Afdn/Lrp8/Kif1a/Cdh2/Ctnna2/Camk2b	34
GO:0034329	cell junction assembly	42/652	441/28814	5.29E-15	2.31E-12	1.74E-12	Nrg2/Syndig1/Il1rapl1/Sdk1/Negr1/Mdga2/Nfasc/Ppp1r9a/Lingo2/Lrfn5/Gpm6a/Clstn3/Pkp4/Nrg1/Bcas3/Nlgn1/Cdh22/Dnm3/Pard3/Limch1/Setd5/Clstn2/Il1rapl2/Dlc1/Map4k4/Pclo/Gpc6/Sptbn2/Nptn/Rock1/Prkca/Ntrk3/Nrxn1/Myo6/Mapt/Afdn/Gabrb2/Fgf13/Erbb4/Dst/Patj/Cdh2	42

GO:0008380	RNA splicing	40/652	404/28814	6.56E-15	2.58E-12	1.94E-12	Nova1/Sf3b2/Setx/Fus/Sfswap/Srek1/Luc7l2/Khdrbs2/Mbnl2/Psip1/Rbfox2/Hnrnpm/Malat1/Sfpq/Srrm4/Fam172a/Rbm28/Snrnp48/Paxbp1/Zc3h13/Srek1ip1/Rnpc3/Rbm25/Luc7l/Prpf38b/Prpf40a/Tcerg1/Rp9/Sart3/Zranb2/Rbfox3/Hnrnpu/Supt6/Srpk2/Son/Snrnp70/Rbm6/Prpf4b/Zfp638/Aff2	40
GO:0099504	synaptic vesicle cycle	30/652	227/28814	8.48E-15	3.03E-12	2.28E-12	Syndig1/Ppfia2/Cadps2/Dgki/Dennd1a/Nlgn1/Rims2/Erc1/Dnm3/Trim9/Dnajc6/Ap2b1/Snap47/Gripap1/Bin1/Syn3/Pclo/Syt2/Syt1/Syn1/Sptbn2/Itsn2/Rock1/Prkca/Nrxn1/Myo6/Fgf14/Cdh2/Canx/Cacnb4	30
GO:0006397	mRNA processing	43/652	472/28814	1.19E-14	3.91E-12	2.94E-12	Nova1/Sf3b2/Setx/Sfswap/Safb2/Srek1/Luc7l2/Khdrbs2/Adarb1/Mbnl2/Psip1/Rbfox2/Hnrnpm/Malat1/Sfpq/Srrm4/Fam172a/Rbm28/Snrnp48/Paxbp1/Zc3h13/Srek1ip1/Rnpc3/Rbm25/Luc7l/Prpf38b/Sltm/Prpf40a/Tcerg1/Akap8l/Sart3/Zranb2/Rbfox3/Hnrnpu/Supt6/Srpk2/Son/Snrnp70/Rbm6/Rbbp6/Prpf4b/Plcb1/Aff2	43
GO:0106027	neuron projection organization	21/652	109/28814	4.84E-14	1.46E-11	1.10E-11	Kalrn/Ppfia2/Shank1/Ppp1r9a/Zfp804a/Atp1a3/Dock10/Nlgn1/Dnm3/Tanc2/Tanc1/Pak3/Map1a/Afdn/Lrp8/Kif1a/Insr/Igf1r/Grin2a/Epha5/Camk2b	21
GO:0031346	positive regulation of cell projection organization	40/652	443/28814	1.38E-13	3.86E-11	2.91E-11	Kalrn/Il1rapl1/Negr1/Setx/Robo2/Plxna4/Ppp1r9a/Lrrc7/Zfp804a/Gpm6a/Nrg1/Bcas3/Nlgn1/Ccdc88a/Dnm3/Grip1/Arap1/Dzip1/Clip1/Espn/Golga4/Ahi1/Tenm3/Tenm1/Sgk1/Nptn/Robo1/Pak3/Ntrk3/Mapt/Afdn/Lrp8/Kif3c/Itpr1/Igf1r/Dscam/Dcc/Cntn1/Camk2b/Alk	40
GO:0048813	dendrite morphogenesis	26/652	187/28814	1.57E-13	4.12E-11	3.10E-11	Kalrn/Il1rapl1/Hecw2/Ppfia2/Shank1/Ppp1r9a/Phactr1/Dock10/Nlgn1/Dnm3/Hecw1/Rbfox2/Tanc2/Grip1/Rere/Sgk1/Sema3a/Robo1/Pak3/Afdn/Lrp8/Kif1a/Epha5/Dscam/Ctnna2/Camk2b	26

GO:0060996	dendritic spine development	22/652	130/28814	1.98E-13	4.86E-11	3.65E-11	Kalrn/Sdk1/Ppfia2/Fstl4/Shank1/Ppp1r9a/Arid1b/Nrg1/Dock10/Nlgn1/Dnm3/Tanc2/Spire1/Slc12a5/Pak3/Afdn/Lrp8/Kif1a/Srgap2/Epha5/Crebbp/Camk2b	22
GO:0099173	postsynapse organization	27/652	207/28814	2.59E-13	5.99E-11	4.50E-11	Kalrn/Il1rapl1/Ppfia2/Gphn/Shank1/Ppp1r9a/Zfp804a/Sorbs2/Dock10/Nlgn1/Dnm3/Tanc2/Snx27/Tanc1/Sptbn2/Pak3/Ntrk3/Nrxn1/Afdn/Lrp8/Kif1a/Insr/Igf1r/Grin2a/Epha5/Cdh2/Camk2b	27
GO:0007626	locomotory behavior	30/652	265/28814	5.31E-13	1.16E-10	8.72E-11	Bc1/Kalrn/Negr1/Chd7/Lsamp/Atp1a3/Gigyf2/Nrg1/Atp1a2/Slc4a10/Sptbn4/Sez6l/Espn/Mapk10/Scn8a/Atxn1/Myo6/Mapt/Kcnma1/Hipk2/Grin2a/Gnao1/Fgf14/Dscam/Cntn1/Calb1/Cacnb4/Pcdh15/Zfhx3/Alk	30
GO:0097061	dendritic spine organization	19/652	101/28814	1.22E-12	2.53E-10	1.90E-10	Kalrn/Ppfia2/Shank1/Ppp1r9a/Zfp804a/Dock10/Nlgn1/Dnm3/Tanc2/Tanc1/Pak3/Afdn/Lrp8/Kif1a/Insr/Igf1r/Grin2a/Epha5/Camk2b	19
GO:0050890	cognition	34/652	359/28814	2.55E-12	5.01E-10	3.76E-10	Kalrn/Chd7/Dgki/Shank1/Atp1a3/Kmt2a/Nrg1/Zzef1/Foxp2/Scn2a/Atp1a2/Csmd1/Slc24a2/Setd5/Chd8/Tanc1/Arl6ip5/Slc12a5/Brinp1/Rp9/Sgk1/Nptn/Atxn1/Plcb1/Prkca/Nrxn1/Ncam1/Mapt/Map1a/Grin2a/Aff2/Fgf13/Crebbp/Calb1	34
GO:0034765	regulation of ion transmembrane transport	40/652	498/28814	5.71E-12	1.07E-09	8.03E-10	Akap7/Nalcn/Hecw2/Chd7/Dpp10/Kcnc2/Shank1/Clic6/Nlgn1/Kcnh7/Scn2a/Akap9/Atp1a2/Hecw1/Kcnip4/Snx27/Wnk2/Arl6ip5/Stk39/Bin1/Rgs7/Stim1/Stac/Slc8a1/Scn8a/Ryr2/Plcb1/Prkca/Pik3c2a/Nrxn1/Kcnma1/Hcn2/Grin2a/Fgf14/Fgf13/Cacng2/Cacnb4/Cacnb2/Cacna1d/Ank3	40
GO:0035418	protein localization to synapse	17/652	87/28814	9.89E-12	1.77E-09	1.33E-09	Kalrn/Gphn/Shank1/Clstn3/Nlgn1/Grip1/Snap47/Gripap1/Pclo/Mapk10/Gpc6/Nrxn1/Mapt/Map1a/Kif5a/Grin2a/Cacng2	17

GO:0007611	learning or memory	31/652	322/28814	1.56E-11	2.67E-09	2.01E-09	Kalrn/Dgki/Shank1/Atp1a3/Kmt2a/Nrg1/Zzef1/Foxp2/Scn2a/Atp1a2/Csmd1/Slc24a2/Chd8/Tanc1/Arl6ip5/Slc12a5/Brinp1/Sgk1/Nptn/Atxn1/Plcb1/Prkca/Nrxn1/Ncam1/Mapt/Map1a/Grin2a/Aff2/Fgf13/Crebbp/Calb1	31
GO:0098742	cell-cell adhesion via plasma-membrane adhesion molecules	25/652	214/28814	2.33E-11	3.82E-09	2.87E-09	Il1rapl1/Sdk1/Mdga2/Robo2/Fat2/Lrrc4c/Lrfn5/Clstn3/Nrg1/Unc5d/Nlgn1/Cdh22/Ntng1/Clstn2/Tenm3/Tenm2/Tenm1/Gpc6/Nptn/Robo1/Nrxn1/Dscam/Cdh2/Cdh13/Pcdh15	25
GO:1902414	protein localization to cell junction	19/652	121/28814	3.41E-11	5.35E-09	4.03E-09	Kalrn/Gphn/Shank1/Clstn3/Nlgn1/Grip1/Snap47/Gripap1/Pclo/Mapk10/Gpc6/Nrxn1/Mapt/Map1a/Afdn/Kif5a/Kif3a/Grin2a/Cacng2	19
GO:0030534	adult behavior	23/652	189/28814	6.40E-11	9.67E-09	7.27E-09	Bc1/Kalrn/Sdk1/Chd7/Shank1/Atp1a3/Gigyf2/Atp1a2/Sptbn4/Sez6l/Sptbn2/Scn8a/Atxn1/Nrxn1/Mapt/Kcnma1/Hipk2/Fgf14/Crebbp/Cacnb4/Pcdh15/Alk/Parp1	23
GO:0035637	multicellular organismal signaling	21/652	157/28814	7.24E-11	1.02E-08	7.70E-09	Tnni3k/Nfasc/Ctnna3/Scn2a/Akap9/Rbfox2/Sptbn4/Bin1/Slc8a1/Slc4a3/Scn8a/Ryr2/Ntrk3/Kcnma1/Grik2/Cacng2/Cacnb4/Cacnb2/Cacna1g/Cacna1d/Ank3	21
GO:0060998	regulation of dendritic spine development	16/652	85/28814	7.29E-11	1.02E-08	7.70E-09	Kalrn/Sdk1/Ppfia2/Fstl4/Shank1/Ppp1r9a/Nrg1/Nlgn1/Dnm3/Tanc2/Pak3/Afdn/Lrp8/Kif1a/Crebbp/Camk2b	16
GO:1990778	protein localization to cell periphery	32/652	370/28814	1.20E-10	1.62E-08	1.22E-08	Kalrn/Nfasc/Dpp10/Gphn/Mrap2/Ccdc88a/Lin7a/Rab31/Kcnip4/Sptbn4/Snx27/Grip1/Snap47/Arl6ip5/Clip1/Gripap1/Golga4/Rabep1/Mapk10/Gpc6/Stac/Rock1/Nrxn1/Afdn/Grin2a/Fgf13/Cdh2/Camk2b/Cacng2/Cacnb2/Ank3/Ank1	32
GO:0050905	neuromuscular process	23/652	198/28814	1.65E-10	2.16E-08	1.62E-08	Shank1/Herc1/Gigyf2/Nrg1/Adarb1/Mycbp2/Camta1/Csmd1/Rbfox2/Chd8/Stac/Scn8a/Nrxn1/Map1a/Kcnma1/Itpr1/Hipk2/Grin2a/Fgf14/Ctnna2/Camk2b/Pcdh15/Parp1	23

GO:0006836	neurotransmitter transport	25/652	242/28814	3.32E-10	3.89E-08	2.92E-08	Ppfia2/Cadps2/Dgki/Ppp1r9a/Nlgn1/Rims2/Erc1/Lin7a/Atp1a2/Trim9/Mctp1/Snap47/Arl6ip5/Syn3/Pclo/Slc6a6/Syt2/Syt1/Syn1/Sptbn2/Slc1a6/Prkca/Nrxn1/Myo6/Cacnb4	25
GO:0000375	RNA splicing, via transesterification reactions	27/652	281/28814	3.36E-10	3.89E-08	2.92E-08	Nova1/Sf3b2/Setx/Sfswap/Luc7l2/Khdrbs2/Mbnl2/Psip1/Rbfox2/Hnrnpm/Malat1/Sfpq/Srrm4/Fam172a/Paxbp1/Rnpc3/Rbm25/Luc7l/Prpf40a/Sart3/Rbfox3/Hnrnpu/Srpk2/Son/Snrnp70/Rbm6/Prpf4b	27
GO:0000377	RNA splicing, via transesterification reactions with bulged	27/652	281/28814	3.36E-10	3.89E-08	2.92E-08	Nova1/Sf3b2/Setx/Sfswap/Luc7l2/Khdrbs2/Mbnl2/Psip1/Rbfox2/Hnrnpm/Malat1/Sfpq/Srrm4/Fam172a/Paxbp1/Rnpc3/Rbm25/Luc7l/Prpf40a/Sart3/Rbfox3/Hnrnpu/Srpk2/Son/Snrnp70/Rbm6/Prpf4b	27
GO:0000398	mRNA splicing, via spliceosome	27/652	281/28814	3.36E-10	3.89E-08	2.92E-08	Nova1/Sf3b2/Setx/Sfswap/Luc7l2/Khdrbs2/Mbnl2/Psip1/Rbfox2/Hnrnpm/Malat1/Sfpq/Srrm4/Fam172a/Paxbp1/Rnpc3/Rbm25/Luc7l/Prpf40a/Sart3/Rbfox3/Hnrnpu/Srpk2/Son/Snrnp70/Rbm6/Prpf4b	27
GO:0051648	vesicle localization	22/652	191/28814	4.84E-10	5.43E-08	4.09E-08	Syndig1/Ppfia2/Nlgn1/Lin7a/Gbf1/Dnm3/Tanc2/Ahi1/Syn3/Pclo/Syn1/Exoc4/Rasgrp1/Nrxn1/Myo6/Kif5a/Kif1a/Fgfr2/Dpysl2/Cdh2/Atp9a/Ap3b2	22
GO:0099175	regulation of postsynapse organization	17/652	111/28814	5.51E-10	6.01E-08	4.52E-08	Kalrn/Il1rapl1/Ppfia2/Ppp1r9a/Zfp804a/Nlgn1/Dnm3/Tanc2/Tanc1/Pak3/Ntrk3/Nrxn1/Afdn/Lrp8/Kif1a/Cdh2/Camk2b	17
GO:0097479	synaptic vesicle localization	13/652	60/28814	7.05E-10	7.49E-08	5.63E-08	Syndig1/Nlgn1/Lin7a/Dnm3/Syn3/Pclo/Syn1/Nrxn1/Kif5a/Fgfr2/Dpysl2/Cdh2/Ap3b2	13
GO:0030900	forebrain development	32/652	398/28814	7.56E-10	7.81E-08	5.88E-08	Chd7/Tacc1/Robo2/Plxna4/Phactr1/Nrg1/Foxp2/Atp1a2/Slc4a10/Trappc9/Dlc1/Atrx/Sema3a/Robo1/Prkg1/Plcb1/Nrg3/Ncam1/Afdn/Lrp8/Lhx1/Kif3a/Kif1a/Gnaq/Srgap2/Fgfr2/Fgf13/Ext1/Erbb4/Epha5/Cdh2/Alk	32

GO:0060997	dendritic spine morphogenesis	14/652	73/28814	8.69E-10	8.75E-08	6.58E-08	Kalrn/Ppfia2/Shank1/Ppp1r9a/Dock10/Nlgn1/Dnm3/Tanc2/Pak3/Afdn/Lrp8/Kif1a/Epha5/Camk2b	14
GO:0010959	regulation of metal ion transport	34/652	446/28814	8.93E-10	8.77E-08	6.60E-08	Akap7/Hecw2/Chd7/Dpp10/Kcnc2/Akap9/Atp1a2/Hecw1/Kcnip4/Sptbn4/Snx27/Wnk2/Stk39/Bin1/Rgs7/Stim1/Stac/Slc8a1/Sgk1/Ryr2/Plcb4/Plcb1/Pik3c2a/Itpr1/Gnaq/Gnao1/Fgf14/Fgf13/Cntn1/Cacnb4/Cacnb2/Cacna1g/Cacna1d/Ank3	34
GO:0001505	regulation of neurotransmitter levels	25/652	254/28814	9.20E-10	8.82E-08	6.63E-08	Ppfia2/Cadps2/Dgki/Ppp1r9a/Nlgn1/Rims2/Erc1/Lin7a/Atp1a2/Trim9/Mctp1/Snap47/Arl6ip5/Syn3/Pclo/Syt2/Syt1/Syn1/Sptbn2/Slc1a6/Prkca/Nrxn1/Myo6/Gad2/Cacnb4	25
GO:1904062	regulation of cation transmembrane transport	31/652	381/28814	1.05E-09	9.58E-08	7.21E-08	Akap7/Hecw2/Chd7/Dpp10/Kcnc2/Shank1/Nlgn1/Akap9/Atp1a2/Hecw1/Kcnip4/Snx27/Wnk2/Stk39/Bin1/Rgs7/Stim1/Stac/Slc8a1/Ryr2/Plcb1/Pik3c2a/Nrxn1/Grin2a/Fgf14/Fgf13/Cacng2/Cacnb4/Cacnb2/Cacna1d/Ank3	31
GO:0001508	action potential	19/652	147/28814	1.05E-09	9.58E-08	7.21E-08	Kcnc2/Ctnna3/Kcnh7/Scn2a/Akap9/Bin1/Scn8a/Ryr2/Ntrk3/Kcnma1/Grin2a/Grik2/Gnaq/Fgf13/Cacnb4/Cacnb2/Cacna1g/Cacna1d/Ank3	19
GO:0032409	regulation of transporter activity	27/652	298/28814	1.24E-09	1.10E-07	8.30E-08	Akap7/Hecw2/Kcnc2/Shank1/Kmt2a/Nlgn1/Akap9/Atp1a2/Hecw1/Snx27/Wnk2/Stk39/Stim1/Stac/Ryr2/Plcb1/Prkca/Nrxn1/Insr/Grin2a/Fgf14/Fgf13/Cacng2/Cacnb4/Cacnb2/Cacna1d/Ank3	27
GO:0097120	receptor localization to synapse	14/652	75/28814	1.26E-09	1.10E-07	8.30E-08	Kalrn/Gphn/Lrrc7/Nlgn1/Cep112/Grip1/Snap47/Gripap1/Mapk10/Gpc6/Nptn/Nrxn1/Kif5a/Cacng2	14
GO:0035725	sodium ion transmembrane transport	19/652	150/28814	1.48E-09	1.27E-07	9.52E-08	Gm13629/Nalcn/Hecw2/Atp1a3/Scn2a/Atp1a2/Hecw1/Slc24a2/Wnk2/Slc4a4/Stk39/Slc6a6/Slc8a1/Scn8a/Plcb1/Hcn2/Fgf14/Fgf13/Ank3	19

GO:0007269	neurotransmitter secretion	21/652	185/28814	1.57E-09	1.28E-07	9.66E-08	Ppfia2/Cadps2/Dgki/Ppp1r9a/Nlgn1/Rims2/Erc1/Lin7a/Trim9/Mctp1/Snap47/Syn3/Pclo/Syt2/Syt1/Syn1/Sptbn2/Prkca/Nrxn1/Myo6/Cacnb4	21
GO:0099643	signal release from synapse	21/652	185/28814	1.57E-09	1.28E-07	9.66E-08	Ppfia2/Cadps2/Dgki/Ppp1r9a/Nlgn1/Rims2/Erc1/Lin7a/Trim9/Mctp1/Snap47/Syn3/Pclo/Syt2/Syt1/Syn1/Sptbn2/Prkca/Nrxn1/Myo6/Cacnb4	21
GO:0031503	protein-containing complex localization	22/652	204/28814	1.70E-09	1.37E-07	1.03E-07	Kalrn/Gphn/Lrrc7/Nrg1/Nlgn1/Dnm3/Akap9/Cep112/Grip1/Ap2b1/Sfpq/Snap47/Dzip1/Gripap1/Hnrnpu/Mapk10/Gpc6/Nptn/Nrxn1/Myo6/Kif5a/Cacng2	22
GO:0010976	positive regulation of neuron projection	23/652	225/28814	2.08E-09	1.63E-07	1.23E-07	Kalrn/Negr1/Setx/Ppp1r9a/Lrrc7/Zfp804a/Nrg1/Clip1/Ahi1/Tenm3/Nptn/Pak3/Ntrk3/Mapt/Afdn/Lrp8/Kif3c/Itpr1/Igf1r/Dcc/Cntn1/Camk2b/Alk	23
GO:0051963	regulation of synapse assembly	17/652	121/28814	2.16E-09	1.67E-07	1.25E-07	Nrg2/Syndig1/Il1rapl1/Negr1/Mdga2/Ppp1r9a/Lingo2/Lrfn5/Clstn3/Nlgn1/Setd5/Clstn2/Il1rapl2/Gpc6/Prkca/Ntrk3/Nrxn1	17
GO:0006814	sodium ion transport	24/652	248/28814	2.80E-09	2.11E-07	1.59E-07	Gm13629/Nalcn/Hecw2/Atp1a3/Slc5a9/Scn2a/Atp1a2/Hecw1/Slc4a10/Sptbn4/Slc24a2/Wnk2/Slc4a4/Stk39/Slc6a6/Slc8a1/Sgk1/Scn8a/Plcb1/Hcn2/Fgf14/Fgf13/Cntn1/Ank3	24
GO:0016570	histone modification	35/652	495/28814	3.62E-09	2.64E-07	1.99E-07	Prmt8/Taf1/Atxn7/Phf20l1/Smyd5/Kmt2c/Chd3/Kmt2a/Mta3/Baz2a/Jmjd1c/Chd4/Arid4b/Kansl1/Rtf1/Usp16/Setd5/Sfpq/Hdac8/Kmt2e/Paxbp1/Brd4/Akap8/Akap8l/Kat6b/Sart3/Rif1/Atrx/Supt6/Prkca/Kat2b/Nsd1/Ncoa1/Daxx/Crebbp	35
GO:0036465	synaptic vesicle recycling	14/652	81/28814	3.63E-09	2.64E-07	1.99E-07	Dennd1a/Nlgn1/Dnm3/Dnajc6/Ap2b1/Gripap1/Bin1/Pclo/Syt2/Syt1/Itsn2/Rock1/Fgf14/Canx	14

GO:0008344	adult locomotory behavior	16/652	111/28814	4.39E-09	3.09E-07	2.32E-07	Bc1/Kalrn/Chd7/Atp1a3/Gigyf2/Atp1a2/Sptbn4/Sez6l/Scn8a/Atxn1/Mapt/Kcnma1/Hipk2/Fgf14/Cacnb4/Pcdh15	16
GO:0032412	regulation of ion transmembrane transporter activity	25/652	274/28814	4.40E-09	3.09E-07	2.32E-07	Akap7/Hecw2/Kcnc2/Shank1/Nlgn1/Akap9/Atp1a2/Hecw1/Snx27/Wnk2/Stk39/Stim1/Stac/Ryr2/Plcb1/Prkca/Nrxn1/Grin2a/Fgf14/Fgf13/Cacng2/Cacnb4/Cacnb2/Cacna1d/Ank3	25
GO:0021537	telencephalon development	24/652	258/28814	6.13E-09	4.22E-07	3.18E-07	Chd7/Tacc1/Robo2/Plxna4/Phactr1/Nrg1/Foxp2/Atp1a2/Trappc9/Sema3a/Robo1/Plcb1/Nrg3/Afdn/Lrp8/Lhx1/Kif3a/Srgap2/Fgf13/Ext1/Erbb4/Epha5/Cdh2/Alk	24
GO:0048639	positive regulation of developmental growth	22/652	220/28814	6.99E-09	4.65E-07	3.50E-07	Chd7/Nrg1/Rims2/Sptbn4/Sgip1/Golga4/Zfpm2/Wnt2/Syt2/Syt1/Itsn2/Ppard/Plcb1/Ntrk3/Ncam1/Mapt/Afdn/Insr/Igf1r/Fgfr2/Erbb4/Dscam	22
GO:1901888	regulation of cell junction assembly	22/652	220/28814	6.99E-09	4.65E-07	3.50E-07	Nrg2/Syndig1/Il1rapl1/Negr1/Mdga2/Ppp1r9a/Lingo2/Lrfn5/Clstn3/Bcas3/Nlgn1/Limch1/Setd5/Clstn2/Il1rapl2/Dlc1/Map4k4/Gpc6/Rock1/Prkca/Ntrk3/Nrxn1	22
GO:0022898	regulation of transmembrane transporter activity	25/652	284/28814	9.09E-09	5.95E-07	4.48E-07	Akap7/Hecw2/Kcnc2/Shank1/Nlgn1/Akap9/Atp1a2/Hecw1/Snx27/Wnk2/Stk39/Stim1/Stac/Ryr2/Plcb1/Prkca/Nrxn1/Grin2a/Fgf14/Fgf13/Cacng2/Cacnb4/Cacnb2/Cacna1d/Ank3	25
GO:0050684	regulation of mRNA processing	18/652	150/28814	9.53E-09	6.14E-07	4.62E-07	Nova1/Sfswap/Safb2/Khdrbs2/Adarb1/Mbnl2/Rbfox2/Malat1/Srrm4/Fam172a/Rbm25/Sltm/Rbfox3/Hnrnpu/Supt6/Srpk2/Son/Snrnp70	18
GO:0010639	negative regulation of organelle organization	29/652	373/28814	1.01E-08	6.37E-07	4.79E-07	Mphosph9/Nav3/Atxn7/Shank1/Ppp1r9a/Fhod3/Toml12/Tbc1d4/Kank1/Trim9/Mphosph8/Arap1/Camsap2/Tmeff2/Espn/Hnrnpu/Dlc1/Hdgfl3/Atrx/Sptan1/Sgk1/Pparg/Kat2b/Mapt/Map1a/Fgfr2/Fgf13/Ctnna2/Parp1	29

GO:0006338	chromatin remodeling	22/652	229/28814	1.46E-08	9.10E-07	6.85E-07	Chd7/Chd2/Arid1b/Chd3/Mta3/Baz2a/Chd4/Rere/Smarcc2/Chd8/Anp32b/Smarca2/Brd4/Kat6b/Sart3/Atrx/Supt6/Ssrp1/Kat2b/Hp1bp3/Daxx/Chd1	22
GO:0000380	alternative mRNA splicing, via spliceosome	13/652	76/28814	1.50E-08	9.19E-07	6.91E-07	Nova1/Sfswap/Khdrbs2/Mbnl2/Rbfox2/Hnrnpm/Malat1/Sfpq/Srrm4/Fam172a/Rbm25/Rbfox3/Hnrnpu	13
GO:0022604	regulation of cell morphogenesis	27/652	338/28814	1.84E-08	1.11E-06	8.38E-07	Kalrn/Il1rapl1/Strip2/Plxna4/Fgd4/Rims2/Kank1/Ntng1/Grip1/Dnmbp/Fmnl2/Arap1/Prpf40a/Dlc1/Sh3d19/Ttc3/Syt2/Syt1/Itsn2/Sgk1/Pak3/Myo10/Afdn/Lrp8/Kif3a/Gas7/Camk2b	27
GO:0051494	negative regulation of cytoskeleton organization	19/652	176/28814	2.15E-08	1.28E-06	9.61E-07	Nav3/Atxn7/Shank1/Ppp1r9a/Fhod3/Kank1/Mphosph8/Arap1/Camsap2/Tmeff2/Espn/Dlc1/Hdgfl3/Sptan1/Sgk1/Kat2b/Map1a/Fgf13/Ctnna2	19
GO:0001764	neuron migration	20/652	196/28814	2.40E-08	1.41E-06	1.06E-06	Cep85l/Mdga2/Gpm6a/Phactr1/Nrg1/Unc5d/Ntng1/Astn2/Sema3a/Prkg1/Ntrk3/Nrg3/Mapt/Lhx1/Srgap2/Fgf13/Erbb4/Dcc/Ctnna2/Camk2b	20
GO:0007411	axon guidance	23/652	258/28814	2.81E-08	1.62E-06	1.22E-06	Kalrn/Cntn4/Nfasc/Robo2/Plxna4/Trio/Unc5d/Mycbp2/Tenm2/Unc5c/Sema3a/Nptn/Robo1/Ncam1/Lhx1/Kif5a/Ext1/Epha5/Dscam/Dcc/Dpysl2/Cntn1/Ank3	23
GO:0007613	memory	18/652	161/28814	2.92E-08	1.66E-06	1.25E-06	Kalrn/Shank1/Atp1a3/Nrg1/Scn2a/Csmd1/Slc24a2/Chd8/Brinp1/Sgk1/Atxn1/Plcb1/Mapt/Map1a/Grin2a/Fgf13/Crebbp/Calb1	18
GO:0097485	neuron projection guidance	23/652	259/28814	3.02E-08	1.70E-06	1.28E-06	Kalrn/Cntn4/Nfasc/Robo2/Plxna4/Trio/Unc5d/Mycbp2/Tenm2/Unc5c/Sema3a/Nptn/Robo1/Ncam1/Lhx1/Kif5a/Ext1/Epha5/Dscam/Dcc/Dpysl2/Cntn1/Ank3	23
GO:0061337	cardiac conduction	12/652	68/28814	3.73E-08	2.07E-06	1.55E-06	Tnni3k/Ctnna3/Akap9/Rbfox2/Sptbn4/Bin1/Slc8a1/Slc4a3/Ryr2/Cacnb2/Cacna1g/Cacna1d	12

GO:0006813	potassium ion transport	22/652	243/28814	4.26E-08	2.33E-06	1.75E-06	Akap7/Nalcn/Dpp10/Kcnc2/Atp1a3/Kcnh7/Akap9/Atp1a2/Kcnip4/Snx27/Slc24a2/Wnk2/Slc12a5/Stk39/Bin1/Rgs7/Plcb4/Kcnma1/Hcn2/Gnaq/Cacna1d/Ank3	22
GO:0061001	regulation of dendritic spine	11/652	56/28814	4.33E-08	2.33E-06	1.75E-06	Kalrn/Ppfia2/Ppp1r9a/Nlgn1/Dnm3/Tanc2/Pak3/Afdn/Lrp8/Kif1a/Camk2b	11
GO:0048638	regulation of developmental growth	29/652	402/28814	5.19E-08	2.75E-06	2.07E-06	Chd7/Fstl4/Plxna4/Nrg1/Rims2/Sptbn4/Sgip1/Golga4/Zfpm2/Wnt2/Syt2/Syt1/Itsn2/Sema3a/Ppard/Plcb1/Ntrk3/Ncam1/Mapt/Afdn/Insr/Igf1r/Fgfr2/Fgf13/Erbb4/Dscam/Daxx/Dpysl2/Atrn	29
GO:0071805	potassium ion transmembrane transport	20/652	206/28814	5.52E-08	2.89E-06	2.18E-06	Akap7/Nalcn/Dpp10/Kcnc2/Atp1a3/Kcnh7/Akap9/Atp1a2/Kcnip4/Snx27/Slc24a2/Wnk2/Slc12a5/Stk39/Bin1/Rgs7/Kcnma1/Hcn2/Cacna1d/Ank3	20
GO:0007612	learning	19/652	187/28814	5.73E-08	2.96E-06	2.23E-06	Kalrn/Dgki/Shank1/Atp1a3/Kmt2a/Zzef1/Foxp2/Atp1a2/Csmd1/Slc24a2/Tanc1/Slc12a5/Sgk1/Nptn/Atxn1/Nrxn1/Map1a/Grin2a/Fgf13	19
GO:0051962	positive regulation of nervous system development	27/652	359/28814	6.42E-08	3.28E-06	2.46E-06	Kalrn/Syndig1/Il1rapl1/Robo2/Plxna4/Lingo2/Clstn3/Nrg1/Nlgn1/Clstn2/Golga4/Bin1/Wnt2/Sgk1/Nptn/Atxn1/Robo1/Pparg/Prkca/Pak3/Ntrk3/Nrxn1/Mapt/Afdn/Lrp8/Dscam/Camk2b	27
GO:0035249	synaptic transmission, glutamatergic	15/652	117/28814	6.68E-08	3.36E-06	2.53E-06	Kalrn/Dgki/Clstn3/Plppr4/Zzef1/Nlgn1/Grik4/Syt1/Nrxn1/Grin2a/Grik2/Ext1/Cdh2/Cacng2/Cacnb4	15
GO:0060078	regulation of postsynaptic membrane	15/652	118/28814	7.49E-08	3.70E-06	2.78E-06	Dgki/Shank1/Ppp1r9a/Nlgn1/Rims2/Rgs7bp/Pclo/Rgs7/Atxn1/Nrxn1/Afdn/Grin2a/Grik2/Gabra6/Fgf14	15
GO:0097091	synaptic vesicle	7/652	18/28814	7.54E-08	3.70E-06	2.78E-06	Syndig1/Nlgn1/Syn3/Pclo/Syn1/Nrxn1/Cdh2	7

GO:0051258	protein polymerization	23/652	276/28814	9.71E-08	4.71E-06	3.54E-06	Nav3/Ppp1r9a/Fhod3/Kank1/Akap9/Pde4dip/Cyfip2/Mphosph8/Spire1/Camsap2/Clip1/Diaph2/Bin1/Hdgfl3/Tenm1/Sptan1/Sgk1/Pstpip2/Pak3/Mapt/Hsp90aa1/Gas7/Fgf13	23
GO:0099172	presynapse organization	11/652	61/28814	1.10E-07	5.26E-06	3.95E-06	Il1rapl1/Mdga2/Lrfn5/Clstn3/Nlgn1/Chd4/Il1rapl2/Pclo/Gpc6/Ntrk3/Nrxn1	11
GO:1903829	positive regulation of cellular protein localization	32/652	491/28814	1.12E-07	5.30E-06	3.99E-06	Malrd1/Myo18a/Dpp10/Clstn3/Nrg1/Bcas3/Nlgn1/Ccdc88a/Hnrnpm/Snx27/Ap2b1/Anp32b/Dzip1/Fbxw7/Tlr4/Stac/Nptn/Pparg/Ppard/Oaz2/Nrxn1/Mapt/Map1a/Kif3a/Hsp90ab1/Erbb4/Cacng2/Cacnb4/Cacna1d/Bicd1/Ank3/Parp1	32
GO:1905606	regulation of presynapse	9/652	38/28814	1.34E-07	6.23E-06	4.69E-06	Il1rapl1/Mdga2/Lrfn5/Clstn3/Nlgn1/Il1rapl2/Gpc6/Ntrk3/Nrxn1	9
GO:0046847	filopodium assembly	12/652	76/28814	1.35E-07	6.23E-06	4.69E-06	Ppp1r9a/Gpm6a/Fgd4/Bcas3/Nlgn1/Dnm3/Arap1/Espn/Tenm1/Nrxn1/Myo10/Srgap2	12
GO:0043414	macromolecule methylation	25/652	329/28814	1.62E-07	7.39E-06	5.56E-06	Prmt8/Thada/Smyd5/Kmt2c/Kmt2a/Baz2a/Arid4b/Ftx/Kansl1/Rtf1/Mphosph8/Setd5/Kmt2e/Paxbp1/Zc3h13/Kcnq1ot1/Brd4/Atf7ip/Rif1/Atrx/Supt6/Nsd1/Meg3/Crebbp/Parp1	25
GO:0035264	multicellular organism growth	20/652	223/28814	2.03E-07	9.19E-06	6.91E-06	Chd7/Kmt2c/Gigyf2/Rasal2/Selenom/Adarb1/Slc4a10/Sptbn4/Ankrd11/Sgip1/Slc12a5/Atrx/Sptbn2/Rbbp6/Nek1/Meg3/Ext1/Daxx/Pcdh15/Atrn	20
GO:0043266	regulation of potassium ion transport	14/652	112/28814	2.51E-07	1.12E-05	8.43E-06	Akap7/Dpp10/Kcnc2/Akap9/Kcnip4/Snx27/Wnk2/Stk39/Bin1/Rgs7/Plcb4/Gnaq/Cacna1d/Ank3	14
GO:0051271	negative regulation of cellular component	24/652	314/28814	2.56E-07	1.13E-05	8.51E-06	Lrch1/Nav3/Frmd5/Nrg1/Adarb1/Kank1/Mctp1/Limch1/Tmeff2/Dlc1/Bin1/Sema3a/Robo1/Prkg1/Pparg/Ppard/Plcb1/Nrg3/Afdn/Mitf/Hmgb1/Srgap2/Erbb4/Dach1	24
GO:0016571	histone methylation	17/652	167/28814	2.77E-07	1.21E-05	9.08E-06	Prmt8/Smyd5/Kmt2c/Kmt2a/Baz2a/Arid4b/Kansl1/Rtf1/Setd5/Kmt2e/Paxbp1/Brd4/Rif1/Atrx/Supt6/Nsd1/Crebbp	17

GO:0002028	regulation of sodium ion transport	13/652	97/28814	2.96E-07	1.28E-05	9.60E-06	Hecw2/Atp1a2/Hecw1/Sptbn4/Wnk2/Stk39/Slc8a1/Sgk1/Plcb1/Fgf14/Fgf13/Cntn1/Ank3	13
GO:1902903	regulation of supramolecular fiber organization	27/652	388/28814	3.06E-07	1.31E-05	9.83E-06	Nav3/Atxn7/Shank1/Ppp1r9a/Fhod3/Ccdc88a/Kank1/Akap9/Pde4dip/Limch1/Cyfip2/Mphosph8/Arap1/Camsap2/Clip1/Tmeff2/Dlc1/Bin1/Hdgfl3/Tenm1/Sptan1/Sgk1/Pak3/Mapt/Map1a/Fgf13/Ctnna2	27
GO:0099054	presynapse assembly	10/652	54/28814	3.21E-07	1.35E-05	1.02E-05	Il1rapl1/Mdga2/Lrfn5/Clstn3/Nlgn1/Il1rapl2/Pclo/Gpc6/Ntrk3/Nrxn1	10
GO:0099174	regulation of presynapse	9/652	42/28814	3.37E-07	1.41E-05	1.06E-05	Il1rapl1/Mdga2/Lrfn5/Clstn3/Nlgn1/Il1rapl2/Gpc6/Ntrk3/Nrxn1	9
GO:2001222	regulation of neuron	10/652	55/28814	3.84E-07	1.59E-05	1.19E-05	Phactr1/Nrg1/Unc5d/Ntng1/Sema3a/Nrg3/Srgap2/Erbb4/Ctnna2/Camk2b	10
GO:0045927	positive regulation of growth	24/652	322/28814	4.05E-07	1.64E-05	1.24E-05	Chd7/Nrg1/Rims2/Sptbn4/Sgip1/Golga4/Ahi1/Zfpm2/Wnt2/Syt2/Syt1/Itsn2/Sgk1/Pard/Plcb1/Ntrk3/Ncam1/Mapt/Afdn/Insr/Igf1r/Fgfr2/Erbb4/Dscam	24
GO:0032411	positive regulation of transporter	15/652	134/28814	4.06E-07	1.64E-05	1.24E-05	Akap7/Kcnc2/Kmt2a/Akap9/Wnk2/Stk39/Stim1/Stac/Ryr2/Plcb1/Fgf14/Cacng2/Cacnb4/Cacnb2/Ank3	15
GO:0051650	establishment of vesicle localization	17/652	172/28814	4.22E-07	1.69E-05	1.27E-05	Ppfia2/Nlgn1/Lin7a/Gbf1/Dnm3/Tanc2/Ahi1/Pclo/Exoc4/Rasgrp1/Myo6/Kif5a/Kif1a/Fgfr2/Dpysl2/Atp9a/Ap3b2	17
GO:0048488	synaptic vesicle	11/652	70/28814	4.72E-07	1.86E-05	1.40E-05	Dennd1a/Nlgn1/Dnm3/Dnajc6/Ap2b1/Bin1/Syt2/Syt1/Itsn2/Rock1/Canx	11
GO:0140238	presynaptic endocytosis	11/652	70/28814	4.72E-07	1.86E-05	1.40E-05	Dennd1a/Nlgn1/Dnm3/Dnajc6/Ap2b1/Bin1/Syt2/Syt1/Itsn2/Rock1/Canx	11
GO:0072659	protein localization to plasma membrane	23/652	302/28814	4.84E-07	1.88E-05	1.42E-05	Kalrn/Nfasc/Dpp10/Mrap2/Ccdc88a/Rab31/Kcnip4/Sptbn4/Snx27/Grip1/Arl6ip5/Gripap1/Golga4/Stac/Rock1/Nrxn1/Afdn/Fgf13/Cdh2/Camk2b/Cacnb2/Ank3/Ank1	23
GO:0009791	post-embryonic development	15/652	136/28814	4.92E-07	1.90E-05	1.43E-05	Gigyf2/Kmt2a/Foxp2/Slc4a10/Atrx/Sox6/Slc8a1/Myt1/Meg3/Smad2/Lhx1/Itpr1/Gnaq/Fgfr2/Dscam	15

GO:0060560	developmental growth involved in morphogenesis	22/652	281/28814	5.37E-07	2.04E-05	1.53E-05	Fstl4/Plxna4/Nrg1/Rims2/Cyfip2/Golga4/Syt2/Syt1/Itsn2/Sema3a/Robo1/Ntrk3/Mapt/Afdn/Lhx1/Hsp90aa1/Hsp90ab1/Fgfr2/Fgf13/Ext1/Dscam/Dpysl2	22
GO:0016079	synaptic vesicle exocytosis	15/652	137/28814	5.41E-07	2.04E-05	1.53E-05	Ppfia2/Cadps2/Dgki/Nlgn1/Rims2/Erc1/Trim9/Snap47/Pclo/Syt2/Syt1/Sptbn2/Prkca/Myo6/Cacnb4	15
GO:0051489	regulation of filopodium	10/652	57/28814	5.44E-07	2.04E-05	1.53E-05	Ppp1r9a/Gpm6a/Bcas3/Nlgn1/Dnm3/Arap1/Espn/Tenm1/Nrxn1/Myo10	10
GO:2000146	negative regulation of cell motility	23/652	305/28814	5.75E-07	2.13E-05	1.60E-05	Lrch1/Nav3/Frmd5/Nrg1/Adarb1/Kank1/Mctp1/Limch1/Tmeff2/Dlc1/Sema3a/Robo1/Prkg1/Pparg/Ppard/Plcb1/Nrg3/Afdn/Mitf/Hmgb1/Srgap2/Erbb4/Dach1	23
GO:0030705	cytoskeleton-dependent intracellular	18/652	198/28814	6.73E-07	2.47E-05	1.86E-05	Nefh/Ppfia2/Ccdc88a/Tanc2/Sfpq/Hnrnpu/Rasgrp1/Nefm/Myo6/Mapt/Map1a/Kif5a/Kif3c/Kif3a/Kif1a/Dst/Bicd1/Ap3b2	18
GO:1903311	regulation of mRNA metabolic process	23/652	308/28814	6.82E-07	2.47E-05	1.86E-05	Nova1/Fus/Sfswap/Gigyf2/Safb2/Tnrc6b/Khdrbs2/Adarb1/Mbnl2/Rbfox2/Hnrnpm/Malat1/Srrm4/Fam172a/Rbm25/Sltm/Rbfox3/Hnrnpu/Supt6/Srpk2/Son/Snrnp70/Rock1	23
GO:1902904	negative regulation of supramolecular	17/652	178/28814	6.86E-07	2.47E-05	1.86E-05	Nav3/Atxn7/Shank1/Ppp1r9a/Fhod3/Kank1/Mphosph8/Arap1/Camsap2/Tmeff2/Dlc1/Hdgfl3/Sptan1/Sgk1/Map1a/Fgf13/Ctnna2	17
GO:0099560	synaptic membrane	8/652	34/28814	7.11E-07	2.54E-05	1.91E-05	Mdga2/Lrrc4c/Lrfn5/Nrg1/Nlgn1/Ntng1/Gpc6/Nrxn1	8
GO:0007015	actin filament organization	29/652	458/28814	7.93E-07	2.81E-05	2.11E-05	Shank1/Ppp1r9a/Sorbs2/Fhod3/Phactr1/Ctnna3/Ccdc88a/Kank1/Limch1/Cyfip2/Mphosph8/Nebl/Arap1/Spire1/Tmeff2/Espn/Diaph2/Dlc1/Bin1/Mprip/Tenm1/Hsp90b1/Sptan1/Pstpip2/Pak3/Myo6/Gas7/Ctnna2/Pcdh15	29
GO:0070507	regulation of microtubule cytoskeleton	16/652	161/28814	8.54E-07	3.00E-05	2.25E-05	Nav3/Atxn7/Akap9/Pde4dip/Camsap2/Clip1/Hnrnpu/Hdgfl3/Sgk1/Rock1/Mapt/Map1a/Fgf13/Dst/Patj/Bicd1	16
GO:1901379	regulation of potassium ion	12/652	90/28814	8.93E-07	3.08E-05	2.32E-05	Akap7/Dpp10/Kcnc2/Akap9/Kcnip4/Snx27/Wnk2/Stk39/Bin1/Rgs7/Cacna1d/Ank3	12

GO:0000381	regulation of alternative mRNA splicing,	10/652	60/28814	8.94E-07	3.08E-05	2.32E-05	Nova1/Khdrbs2/Mbnl2/Rbfox2/Malat1/Srrm4/Fam172a/Rbm25/Rbfox3/Hnrnpu	10
GO:0032886	regulation of microtubule-based process	21/652	268/28814	9.55E-07	3.26E-05	2.45E-05	Nefh/Tacc1/Nav3/Atxn7/Cep76/Akap9/Pde4dip/Camsap2/Clip1/Hnrnpu/Hdgfl3/Sgk1/Rock1/Kat2b/Mapt/Map1a/Kif3a/Fgf13/Dst/Patj/Bicd1	21
GO:0043484	regulation of RNA splicing	16/652	163/28814	1.01E-06	3.41E-05	2.57E-05	Nova1/Setx/Fus/Sfswap/Khdrbs2/Mbnl2/Rbfox2/Malat1/Srrm4/Fam172a/Rbm25/Rbfox3/Hnrnpu/Son/Snrnp70/Aff2	16
GO:0030336	negative regulation of cell migration	22/652	292/28814	1.02E-06	3.44E-05	2.59E-05	Lrch1/Nav3/Nrg1/Adarb1/Kank1/Mctp1/Limch1/Tmeff2/Dlc1/Sema3a/Robo1/Prkg1/Pparg/Ppard/Plcb1/Nrg3/Afdn/Mitf/Hmgb1/Srgap2/Erbb4/Dach1	22
GO:0040013	negative regulation of locomotion	24/652	340/28814	1.07E-06	3.54E-05	2.66E-05	Lrch1/Robo2/Nav3/Frmd5/Nrg1/Adarb1/Kank1/Mctp1/Limch1/Tmeff2/Dlc1/Sema3a/Robo1/Prkg1/Pparg/Ppard/Plcb1/Nrg3/Afdn/Mitf/Hmgb1/Srgap2/Erbb4/Dach1	24
GO:0032414	positive regulation of ion	14/652	126/28814	1.08E-06	3.54E-05	2.66E-05	Akap7/Kcnc2/Akap9/Wnk2/Stk39/Stim1/Stac/Ryr2/Plcb1/Fgf14/Cacng2/Cacnb4/Cacnb2/Ank3	14
GO:0006887	exocytosis	27/652	414/28814	1.08E-06	3.54E-05	2.66E-05	Il1rapl1/Ppfia2/Cadps2/Dgki/Slc17a9/Nlgn1/Rims2/Erc1/Lin7a/Exoc6/Rab31/Trim9/Snap47/Pclo/Syt2/Syt1/Sptbn2/Exoc4/Rasgrp1/Prkca/Pik3c2a/Nrxn1/Ncam1/Myo6/Cacnb4/Cacna1g/Atp9a	27
GO:0099072	regulation of postsynaptic membrane	12/652	93/28814	1.28E-06	4.11E-05	3.09E-05	Kalrn/Gphn/Nrg1/Dnm3/Grip1/Ap2b1/Snap47/Gripap1/Mapk10/Gpc6/Myo6/Cacng2	12
GO:0140029	exocytic process	12/652	93/28814	1.28E-06	4.11E-05	3.09E-05	Cadps2/Nlgn1/Rims2/Erc1/Exoc6/Snap47/Pclo/Syt2/Syt1/Exoc4/Nrxn1/Ncam1	12
GO:0021953	central nervous system neuron differentiation	18/652	207/28814	1.29E-06	4.11E-05	3.09E-05	Plxna4/Herc1/Gigyf2/Foxp2/Adarb1/Mycbp2/Slc4a10/Sptbn4/Sox5/Sema3a/Prkca/Nrxn1/Lhx1/Hsp90aa1/Hsp90ab1/Gnaq/Fgfr2/Dcc	18

GO:0032535	regulation of cellular component size	26/652	393/28814	1.31E-06	4.14E-05	3.12E-05	Fstl4/Plxna4/Ppp1r9a/Fhod3/Nrg1/Kank1/Cyfip2/Mphosph8/Slc12a5/Golga4/Bin1/Pclo/Tenm1/Sptan1/Sema3a/Pak3/Ntrk3/Nefm/Mapt/Kcnma1/Hsp90ab1/Hp1bp3/Fgf13/Dscam/Dpysl2/Arhgap5	26
GO:0021954	central nervous system neuron development	12/652	94/28814	1.43E-06	4.51E-05	3.39E-05	Plxna4/Adarb1/Mycbp2/Slc4a10/Sptbn4/Sema3a/Prkca/Hsp90aa1/Hsp90ab1/Gnaq/Fgfr2/Dcc	12
GO:0031497	chromatin assembly	16/652	168/28814	1.51E-06	4.70E-05	3.54E-05	Ubn2/Baz2a/Mphosph8/Fam172a/Anp32b/Smarca2/Kcnq1ot1/Atf7ip/Kat6b/Sart3/Rif1/Atrx/Meg3/Hp1bp3/Hmgb1/Daxx	16
GO:0007156	homophilic cell adhesion via plasma membrane	13/652	112/28814	1.58E-06	4.86E-05	3.65E-05	Sdk1/Robo2/Fat2/Clstn3/Cdh22/Clstn2/Tenm3/Nptn/Robo1/Dscam/Cdh2/Cdh13/Pcdh15	13
GO:0048024	regulation of mRNA splicing, via spliceosome	13/652	112/28814	1.58E-06	4.86E-05	3.65E-05	Nova1/Sfswap/Khdrbs2/Mbnl2/Rbfox2/Malat1/Srrm4/Fam172a/Rbm25/Rbfox3/Hnrnpu/Son/Snrnp70	13
GO:0071824	protein-DNA complex subunit organization	17/652	190/28814	1.70E-06	5.19E-05	3.90E-05	Bdp1/Taf1/Chd2/Taf3/Smarcc2/Anp32b/Smarca2/Atf7ip/Kat6b/Sart3/Atrx/Supt6/Ssrp1/Hp1bp3/Hmgb1/Daxx/Chd1	17
GO:0031110	regulation of microtubule polymerization	12/652	96/28814	1.80E-06	5.44E-05	4.09E-05	Nav3/Atxn7/Akap9/Pde4dip/Camsap2/Clip1/Hdgfl3/Sgk1/Mapt/Map1a/Fgf13/Dst	12
GO:0032259	methylation	25/652	376/28814	1.91E-06	5.71E-05	4.29E-05	Prmt8/Thada/Smyd5/Kmt2c/Kmt2a/Baz2a/Arid4b/Ftx/Kansl1/Rtf1/Mphosph8/Setd5/Kmt2e/Paxbp1/Zc3h13/Kcnq1ot1/Brd4/Atf7ip/Rif1/Atrx/Supt6/Nsd1/Meg3/Crebbp/Parp1	25
GO:0060999	positive regulation of	10/652	65/28814	1.92E-06	5.71E-05	4.29E-05	Kalrn/Shank1/Ppp1r9a/Nrg1/Nlgn1/Pak3/Afdn/Lrp8/Crebbp/Camk2b	10
GO:0051656	establishment of organelle localization	27/652	427/28814	1.94E-06	5.74E-05	4.32E-05	Nefh/Ppfia2/Tacc1/Cdc42bpa/Nlgn1/Lin7a/Gbf1/Dnm3/Clmn/Pard3/Tanc2/Spire1/Ahi1/Hnrnpu/Pclo/Exoc4/Rasgrp1/Myo6/Mapt/Kif5a/Kif3c/Kif1a/Fgfr2/Dpysl2/Bicd1/Atp9a/Ap3b2	27

GO:0070252	actin-mediated cell contraction	12/652	98/28814	2.25E-06	6.59E-05	4.95E-05	Ctnna3/Akap9/Atp1a2/Pard3/Limch1/Bin1/Ryr2/Rock1/Fgf13/Cacnb2/Cacna1g/Cacna1d	12
GO:0060047	heart contraction	19/652	238/28814	2.37E-06	6.89E-05	5.18E-05	Tnni3k/Atp1a3/Ctnna3/Akap9/Atp1a2/Rbfox2/Sptbn4/Bin1/Slc8a1/Slc4a3/Sema3a/Ryr2/Prkca/Gnao1/Fgf13/Ext1/Cacnb2/Cacna1g/Cacna1d	19
GO:0034968	histone lysine methylation	14/652	135/28814	2.47E-06	7.15E-05	5.38E-05	Smyd5/Kmt2c/Kmt2a/Baz2a/Arid4b/Kansl1/Rtf1/Setd5/Kmt2e/Brd4/Rif1/Atrx/Supt6/Nsd1	14
GO:0008016	regulation of heart contraction	17/652	197/28814	2.80E-06	7.97E-05	5.99E-05	Tnni3k/Ctnna3/Akap9/Atp1a2/Rbfox2/Sptbn4/Bin1/Slc8a1/Slc4a3/Sema3a/Ryr2/Prkca/Gnao1/Fgf13/Cacnb2/Cacna1g/Cacna1d	17
GO:0031056	regulation of histone modification	17/652	197/28814	2.80E-06	7.97E-05	5.99E-05	Kmt2a/Baz2a/Kansl1/Rtf1/Setd5/Hdac8/Kmt2e/Paxbp1/Brd4/Akap8/Akap8l/Sart3/Rif1/Atrx/Supt6/Nsd1/Daxx	17
GO:0050773	regulation of dendrite development	14/652	137/28814	2.95E-06	8.33E-05	6.27E-05	Kalrn/Il1rapl1/Hecw2/Csmd3/Hecw1/Camsap2/Clip1/Sgk1/Robo1/Pak3/Afdn/Lrp8/Camk2b/Alk	14
GO:0032970	regulation of actin filament-based process	26/652	414/28814	3.39E-06	9.53E-05	7.16E-05	Shank1/Ppp1r9a/Fhod3/Ctnna3/Bcas3/Ccdc88a/Kank1/Akap9/Atp1a2/Pard3/Limch1/Cyfip2/Mphosph8/Arap1/Tmeff2/Dlc1/Bin1/Tenm1/Sptan1/Ryr2/Rock1/Pak3/Ntrk3/Fgf13/Epha5/Ctnna2	26
GO:0007254	JNK cascade	16/652	180/28814	3.73E-06	0.00010384	7.81E-05	Taok3/Fgd4/Spag9/Gps2/Mfhas1/Map4k4/Mapk10/Tlr4/Rasgrp1/Plcb1/Igf1r/Hmgb1/Hipk2/Grik2/Fgf14/Daxx	16
GO:0140058	neuron projection	7/652	30/28814	3.81E-06	0.00010532	7.92E-05	Phactr1/Nlgn1/Ntng1/Grip1/Sema3a/Rock1/Afdn	7
GO:1903539	protein localization to	9/652	56/28814	4.32E-06	0.0001173	8.82E-05	Kalrn/Gphn/Grip1/Snap47/Gripap1/Mapk10/Gpc6/Grin2a/Cacng2	9
GO:2000649	regulation of sodium ion transmembrane	9/652	56/28814	4.32E-06	0.0001173	8.82E-05	Hecw2/Atp1a2/Hecw1/Wnk2/Stk39/Plcb1/Fgf14/Fgf13/Ank3	9

GO:0051403	stress-activated MAPK cascade	19/652	248/28814	4.33E-06	0.0001173	8.82E-05	Taok3/Fgd4/Spag9/Arl6ip5/Gps2/Stk39/Mfhas1/Map4k4/Mapk10/Map3k3/Tlr4/Rasgrp1/Plcb1/Igf1r/Hmgb1/Hipk2/Grik2/Fgf14/Daxx	19
GO:0018205	peptidyl-lysine modification	25/652	395/28814	4.56E-06	0.00012263	9.22E-05	Taf1/Atxn7/Phf20l1/Smyd5/Kmt2c/Kmt2a/Baz2a/Arid4b/Kansl1/Rtf1/Senp2/Setd5/Hdac8/Kmt2e/Senp7/Brd4/Kat6b/Rif1/Desi1/Atrx/Supt6/Kat2b/Nsd1/Ncoa1/Crebbp	25
GO:0043087	regulation of GTPase activity	23/652	345/28814	4.66E-06	0.00012465	9.37E-05	Kalrn/Lrch1/Rasgef1b/Dgki/Plxna4/Pkp4/Dennd1a/Arap2/Tbc1d4/Dock10/Bcas3/Chn2/Arap1/Bin1/Map4k4/Rgs7/Rasgrp1/Prkg1/Ntrk3/Afdn/Gnao1/Srgap2/Epha5	23
GO:0003015	heart process	19/652	250/28814	4.86E-06	0.00012912	9.71E-05	Tnni3k/Atp1a3/Ctnna3/Akap9/Atp1a2/Rbfox2/Sptbn4/Bin1/Slc8a1/Slc4a3/Sema3a/Ryr2/Prkca/Gnao1/Fgf13/Ext1/Cacnb2/Cacna1g/Cacna1d	19
GO:0062237	protein localization to	9/652	57/28814	5.02E-06	0.00013162	9.90E-05	Kalrn/Gphn/Grip1/Snap47/Gripap1/Mapk10/Gpc6/Grin2a/Cacng2	9
GO:0086001	cardiac muscle cell action	9/652	57/28814	5.02E-06	0.00013162	9.90E-05	Ctnna3/Akap9/Bin1/Ryr2/Fgf13/Cacnb2/Cacna1g/Cacna1d/Ank3	9
GO:0006479	protein methylation	17/652	207/28814	5.46E-06	0.0001412	0.0001062	Prmt8/Smyd5/Kmt2c/Kmt2a/Baz2a/Arid4b/Kansl1/Rtf1/Setd5/Kmt2e/Paxbp1/Brd4/Rif1/Atrx/Supt6/Nsd1/Crebbp	17
GO:0008213	protein alkylation	17/652	207/28814	5.46E-06	0.0001412	0.0001062	Prmt8/Smyd5/Kmt2c/Kmt2a/Baz2a/Arid4b/Kansl1/Rtf1/Setd5/Kmt2e/Paxbp1/Brd4/Rif1/Atrx/Supt6/Nsd1/Crebbp	17
GO:0021955	central nervous system neuron	8/652	44/28814	5.69E-06	0.00014603	0.0001098	Plxna4/Adarb1/Mycbp2/Sptbn4/Prkca/Hsp90aa1/Hsp90ab1/Dcc	8
GO:0048588	developmental cell growth	20/652	277/28814	5.97E-06	0.00015229	0.0001145	Fstl4/Plxna4/Sorbs2/Nrg1/Rims2/Cyfip2/Golga4/Syt2/Syt1/Itsn2/Sema3a/Ntrk3/Mapt/Afdn/Hsp90aa1/Hsp90ab1/Fgf13/Ext1/Dscam/Dpysl2	20
GO:0032271	regulation of protein polymerization	17/652	209/28814	6.21E-06	0.00015744	0.0001184	Nav3/Ppp1r9a/Fhod3/Kank1/Akap9/Pde4dip/Cyfip2/Mphosph8/Camsap2/Clip1/Bin1/Tenm1/Sptan1/Sgk1/Pak3/Mapt/Hsp90aa1	17

GO:0043270	positive regulation of ion transport	22/652	329/28814	7.12E-06	0.00017941	0.0001349	Akap7/Kcnc2/Akap9/Wnk2/Gabbr1/Stk39/Rgs7/Syt1/Stim1/Stac/Sgk1/Ryr2/Plcb1/Itpr1/Fgf14/Dpysl2/Cntn1/Cacng2/Cacnb4/Cacnb2/Cacna1d/Ank3	22
GO:0060079	excitatory postsynaptic	11/652	92/28814	7.48E-06	0.00018728	0.0001408	Dgki/Shank1/Ppp1r9a/Nlgn1/Rims2/Pclo/Atxn1/Nrxn1/Afdn/Grin2a/Grik2	11
GO:0031098	stress-activated protein kinase signaling	19/652	258/28814	7.65E-06	0.00019033	0.0001431	Taok3/Fgd4/Spag9/Arl6ip5/Gps2/Stk39/Mfhas1/Map4k4/Mapk10/Map3k3/Tlr4/Rasgrp1/Plcb1/Igf1r/Hmgb1/Hipk2/Grik2/Fgf14/Daxx	19
GO:0030048	actin filament-based movement	13/652	129/28814	7.72E-06	0.00019081	0.0001435	Ctnna3/Akap9/Atp1a2/Pard3/Limch1/Bin1/Ryr2/Rock1/Myo6/Fgf13/Cacnb2/Cacna1g/Cacna1d	13
GO:1904064	positive regulation of cation	15/652	170/28814	8.15E-06	0.00020007	0.0001505	Akap7/Kcnc2/Akap9/Wnk2/Rgs7/Stim1/Stac/Ryr2/Plcb1/Fgf14/Cacng2/Cacnb4/Cacnb2/Cacna1d/Ank3	15
GO:0018022	peptidyl-lysine methylation	14/652	150/28814	8.52E-06	0.00020801	0.0001564	Smyd5/Kmt2c/Kmt2a/Baz2a/Arid4b/Kansl1/Rtf1/Setd5/Kmt2e/Brd4/Rif1/Atrx/Supt6/Nsd1	14
GO:0034767	positive regulation of ion	16/652	193/28814	9.10E-06	0.00022073	0.000166	Akap7/Kcnc2/Akap9/Wnk2/Stk39/Rgs7/Stim1/Stac/Ryr2/Plcb1/Fgf14/Cacng2/Cacnb4/Cacnb2/Cacna1d/Ank3	16
GO:0002027	regulation of heart rate	12/652	112/28814	9.17E-06	0.00022098	0.0001662	Tnni3k/Ctnna3/Akap9/Rbfox2/Sptbn4/Bin1/Slc8a1/Sema3a/Ryr2/Cacnb2/Cacna1g/Cacna1d	12
GO:0007628	adult walking behavior	8/652	47/28814	9.51E-06	0.00022772	0.0001713	Chd7/Sptbn4/Scn8a/Mapt/Kcnma1/Hipk2/Cacnb4/Pcdh15	8
GO:0019226	transmission of nerve impulse	11/652	95/28814	1.02E-05	0.00024203	0.000182	Nfasc/Scn2a/Sptbn4/Scn8a/Ntrk3/Kcnma1/Grik2/Cacng2/Cacnb4/Cacna1g/Ank3	11
GO:0031060	regulation of histone	11/652	95/28814	1.02E-05	0.00024203	0.000182	Kmt2a/Baz2a/Kansl1/Rtf1/Kmt2e/Paxbp1/Brd4/Rif1/Atrx/Supt6/Nsd1	11
GO:0034728	nucleosome organization	12/652	116/28814	1.32E-05	0.00030945	0.0002327	Chd2/Smarcc2/Anp32b/Smarca2/Kat6b/Sart3/Atrx/Supt6/Ssrp1/Hp1bp3/Daxx/Chd1	12
GO:0043271	negative regulation of ion transport	15/652	177/28814	1.32E-05	0.0003098	0.000233	Hecw2/Atp1a2/Hecw1/Snx27/Wnk2/Arl6ip5/Gabbr1/Stk39/Bin1/Plcb4/Prkca/Pik3c2a/Gnaq/Gnao1/Ank3	15

GO:0051491	positive regulation of	7/652	36/28814	1.39E-05	0.00032255	0.0002426	Gpm6a/Bcas3/Nlgn1/Dnm3/Arap1/Espn/Tenm1	7
GO:0030902	hindbrain development	15/652	178/28814	1.42E-05	0.00032747	0.0002463	Herc1/Foxp2/Rbfox2/Pdss2/Rere/Sez6l/Ahi1/Dlc1/Sptbn2/Nrxn1/Lhx1/Igf1r/Cntn1/Ctnna2/Atrn	15
GO:0010038	response to metal ion	20/652	295/28814	1.51E-05	0.0003462	0.0002604	Gphn/Fus/Nlgn1/Cpne8/Spag16/Syt2/Syt1/Stim1/Ryr2/Pparg/Pde1c/Nrxn1/Ncam1/Mapt/Kcnma1/Grin2a/Daxx/Camk2b/Cacna1g/Ank3	20
GO:0090659	walking behavior	8/652	50/28814	1.53E-05	0.00034924	0.0002626	Chd7/Sptbn4/Scn8a/Mapt/Kcnma1/Hipk2/Cacnb4/Pcdh15	8
GO:0031058	positive regulation of	12/652	118/28814	1.57E-05	0.00035566	0.0002675	Kmt2a/Baz2a/Kansl1/Rtf1/Kmt2e/Paxbp1/Brd4/Akap8/Akap8l/Sart3/Rif1/Daxx	12
GO:0001964	startle response	7/652	37/28814	1.68E-05	0.00037672	0.0002833	Nrg1/Csmd1/Chd8/Nrxn1/Grin2a/Ctnna2/Pcdh15	7
GO:0086091	regulation of heart rate by	7/652	37/28814	1.68E-05	0.00037672	0.0002833	Ctnna3/Akap9/Rbfox2/Bin1/Cacnb2/Cacna1g/Cacna1d	7
GO:2001257	regulation of cation channel activity	15/652	181/28814	1.73E-05	0.00038592	0.0002902	Akap7/Kcnc2/Shank1/Nlgn1/Snx27/Stim1/Stac/Nrxn1/Grin2a/Fgf14/Fgf13/Cacng2/Cacnb4/Cacnb2/Ank3	15
GO:0050769	positive regulation of neurogenesis	20/652	298/28814	1.74E-05	0.00038717	0.0002912	Kalrn/Il1rapl1/Robo2/Plxna4/Nrg1/Golga4/Bin1/Wnt2/Sgk1/Nptn/Atxn1/Robo1/Pparg/Pak3/Ntrk3/Mapt/Afdn/Lrp8/Dscam/Camk2b	20
GO:0050767	regulation of neurogenesis	26/652	455/28814	1.79E-05	0.00039582	0.0002977	Kalrn/Il1rapl1/Chd7/Fstl4/Robo2/Plxna4/Nrg1/Brinp1/Golga4/Bin1/Wnt2/Sgk1/Sema3a/Nptn/Atxn1/Robo1/Pparg/Pak3/Ntrk3/Mapt/Afdn/Lrp8/Fgf13/Dscam/Camk2b/Arntl	26
GO:1990138	neuron projection extension	16/652	204/28814	1.82E-05	0.00039977	0.0003006	Plxna4/Nrg1/Rims2/Cyfip2/Golga4/Syt2/Syt1/Itsn2/Sema3a/Ntrk3/Mapt/Afdn/Hsp90aa1/Hsp90ab1/Dscam/Dpysl2	16
GO:0008306	associative learning	12/652	120/28814	1.86E-05	0.00040542	0.0003049	Shank1/Atp1a3/Kmt2a/Zzef1/Atp1a2/Csmd1/Tanc1/Sgk1/Nptn/Atxn1/Map1a/Grin2a	12

GO:0043254	regulation of protein-containing complex assembly	25/652	429/28814	1.87E-05	0.00040542	0.0003049	Nav3/Ppp1r9a/Fhod3/Nrg1/Kank1/Akap9/Trim9/Pde4dip/Cyfip2/Mphosph8/Camsap2/Clip1/Atf7ip/Bin1/Abca3/Tenm1/Tlr4/Sptan1/Sgk1/Pak3/Ncam1/Mapt/Hsp90aa1/Hmgb1/Parp1	25
GO:1902305	regulation of sodium ion	9/652	67/28814	1.95E-05	0.00042174	0.0003172	Hecw2/Atp1a2/Hecw1/Wnk2/Stk39/Plcb1/Fgf14/Fgf13/Ank3	9
GO:0008088	axo-dendritic transport	10/652	84/28814	2.01E-05	0.00043227	0.0003251	Sfpq/Hnrnpu/Nefm/Mapt/Map1a/Kif5a/Kif3a/Kif1a/Dst/Ap3b2	10
GO:0048167	regulation of synaptic plasticity	23/652	378/28814	2.04E-05	0.00043484	0.000327	Kalrn/Dgki/Mir124a-1hg/Ppp1r9a/Kmt2a/Zzef1/Nlgn1/Grid2ip/Rims2/Erc1/Slc4a10/Slc24a2/Snap47/Syn1/Nptn/Myo6/Mapt/Map1a/Grin2a/Grik2/Fgf14/Camk2b/Calb1	23
GO:1902473	regulation of protein	6/652	26/28814	2.05E-05	0.00043591	0.0003278	Kalrn/Clstn3/Nlgn1/Gripap1/Gpc6/Mapt	6
GO:0034764	positive regulation of transmembrane	18/652	253/28814	2.10E-05	0.00044361	0.0003336	Akap7/Kcnc2/Akap9/Wnk2/Stk39/Rgs7/Stim1/Stac/Ryr2/Plcb1/Insr/Fgf14/Erbb4/Cacng2/Cacnb4/Cacnb2/Cacna1d/Ank3	18
GO:0099565	chemical synaptic	11/652	103/28814	2.22E-05	0.00046529	0.0003499	Dgki/Shank1/Ppp1r9a/Nlgn1/Rims2/Pclo/Atxn1/Nrxn1/Afdn/Grin2a/Grik2	11
GO:0051966	regulation of synaptic	10/652	85/28814	2.24E-05	0.00046529	0.0003499	Kalrn/Dgki/Plppr4/Nlgn1/Syt1/Nrxn1/Grin2a/Grik2/Cdh2/Cacng2	10
GO:0050770	regulation of axonogenesis	15/652	185/28814	2.24E-05	0.00046529	0.0003499	Fstl4/Robo2/Plxna4/Lrrc4c/Nrg1/Golga4/Sema3a/Robo1/Pak3/Ntrk3/Nefm/Mapt/Fgf13/Dscam/Cdh2	15
GO:0043547	positive regulation of GTPase activity	18/652	256/28814	2.46E-05	0.00050811	0.0003821	Kalrn/Rasgef1b/Pkp4/Dennd1a/Arap2/Tbc1d4/Dock10/Bcas3/Chn2/Arap1/Bin1/Map4k4/Rgs7/Rasgrp1/Ntrk3/Afdn/Gnao1/Srgap2	18
GO:0098876	vesicle-mediated transport to	13/652	144/28814	2.53E-05	0.00052137	0.0003921	Dennd1a/Exoc6/Rab31/Snx27/Grip1/Snap47/Gripap1/Golga4/Rabep1/Mapk10/Exoc4/Atp9a/Ank3	13

GO:0070997	neuron death	25/652	438/28814	2.64E-05	0.00053939	0.0004056	Ncoa7/Scn2a/Adarb1/Trim2/Ppp2r2b/Ap2b1/Cpeb4/Arl6ip5/Fbxw7/Tlr4/Srpk2/Rock1/Polb/Pak3/Mapt/Kcnma1/Itpr1/Hsp90ab1/Hipk2/Grik2/Gabrb2/Dcc/Daxx/Cntfr/Parp1	25
GO:0048511	rhythmic process	20/652	307/28814	2.67E-05	0.00054379	0.0004089	Setx/Enox1/Kmt2a/Nlgn1/Mycbp2/Sfpq/Hnrnpu/Fbxl17/Fbxw7/Mapk10/Pparg/Kat2b/Ntrk3/Kcnma1/Igf1r/Grin2a/Crebbp/Zfhx3/Arntl/Alb	20
GO:0050885	neuromuscular process	9/652	70/28814	2.80E-05	0.00056374	0.0004239	Shank1/Herc1/Gigyf2/Camta1/Rbfox2/Nrxn1/Kcnma1/Camk2b/Pcdh15	9
GO:0086003	cardiac muscle cell	9/652	70/28814	2.80E-05	0.00056374	0.0004239	Ctnna3/Akap9/Atp1a2/Bin1/Ryr2/Fgf13/Cacnb2/Cacna1g/Cacna1d	9
GO:0010970	transport along microtubule	14/652	167/28814	2.88E-05	0.00057735	0.0004342	Nefh/Sfpq/Hnrnpu/Rasgrp1/Nefm/Mapt/Map1a/Kif5a/Kif3c/Kif3a/Kif1a/Dst/Bicd1/Ap3b2	14
GO:0030100	regulation of endocytosis	17/652	237/28814	3.19E-05	0.00063551	0.0004779	Nrg1/Nlgn1/Rab31/Pard3/Mctp1/Sgip1/Dnajc6/Ap2b1/Ahi1/Bin1/Rock1/Pparg/Prkca/Kif3a/Insr/Cdh13/Bicd1	17
GO:0031109	microtubule polymerization	12/652	128/28814	3.55E-05	0.00070427	0.0005296	Nav3/Atxn7/Akap9/Pde4dip/Camsap2/Clip1/Hdgfl3/Sgk1/Mapt/Map1a/Fgf13/Dst	12
GO:0048863	stem cell differentiation	17/652	240/28814	3.74E-05	0.00073752	0.0005546	Chd2/Gpm6a/Smyd5/Nrg1/Msi2/Fam172a/Hnrnpu/Esrrb/Tcof1/Sox6/Sox5/Sema3a/Kitl/Fgfr2/Ext1/Erbb4/Cdh2	17
GO:0008542	visual learning	9/652	73/28814	3.93E-05	0.00077262	0.000581	Atp1a3/Kmt2a/Zzef1/Atp1a2/Tanc1/Sgk1/Nptn/Atxn1/Grin2a	9
GO:0031365	N-terminal protein amino	6/652	29/28814	4.00E-05	0.00077748	0.0005847	Naa15/Naa16/Metap2/Kat2b/Nmt2/Crebbp	6
GO:0060045	positive regulation of cardiac muscle	6/652	29/28814	4.00E-05	0.00077748	0.0005847	Nrg1/Zfpm2/Wnt2/Ncam1/Fgfr2/Erbb4	6
GO:0021549	cerebellum development	11/652	111/28814	4.47E-05	0.00086611	0.0006513	Herc1/Foxp2/Pdss2/Rere/Sez6l/Sptbn2/Nrxn1/Lhx1/Igf1r/Cntn1/Atrn	11
GO:0018023	peptidyl-lysine trimethylation	8/652	58/28814	4.66E-05	0.00089256	0.0006712	Smyd5/Kmt2a/Arid4b/Rtf1/Setd5/Kmt2e/Brd4/Atrx	8

GO:0031062	positive regulation of	8/652	58/28814	4.66E-05	0.00089256	0.0006712	Kmt2a/Baz2a/Kansl1/Rtf1/Kmt2e/Paxbp1/Brd4/Rif1	8
GO:0048814	regulation of dendrite	10/652	93/28814	4.91E-05	0.00093684	0.0007045	Kalrn/Il1rapl1/Hecw2/Hecw1/Sgk1/Robo1/Pak3/Afdn/Lrp8/Camk2b	10
GO:0035304	regulation of protein	10/652	94/28814	5.39E-05	0.00101395	0.0007625	Camta1/Ppp4r4/Ppp2r2b/Mfhas1/Dlc1/Mprip/Hsp90b1/Rock1/Ppp1r12a/Hsp90ab1	10
GO:0051899	membrane depolarization	10/652	94/28814	5.39E-05	0.00101395	0.0007625	Scn2a/Scn8a/Hcn2/Cnga1/Cacng2/Cacnb2/Cacna1g/Cacna1d/Ank3/Parp1	10
GO:0006305	DNA alkylation	9/652	76/28814	5.43E-05	0.00101395	0.0007625	Kmt2a/Baz2a/Ftx/Mphosph8/Kmt2e/Kcnq1ot1/Atf7ip/Meg3/Parp1	9
GO:0006306	DNA methylation	9/652	76/28814	5.43E-05	0.00101395	0.0007625	Kmt2a/Baz2a/Ftx/Mphosph8/Kmt2e/Kcnq1ot1/Atf7ip/Meg3/Parp1	9
GO:0050919	negative chemotaxis	7/652	44/28814	5.45E-05	0.00101395	0.0007625	Robo2/Plxna4/Nrg1/Unc5c/Sema3a/Robo1/Nrg3	7
GO:1905475	regulation of protein localization to	15/652	200/28814	5.53E-05	0.0010243	0.0007703	Kalrn/Dpp10/Mrap2/Sorbs2/Gpc5/Snx27/Ap2b1/Gripap1/Gpc6/Stac/Nrxn1/Cdh2/Camk2b/Cacng2/Ank3	15
GO:0086010	membrane depolarization	6/652	31/28814	5.96E-05	0.00109461	0.0008232	Scn2a/Scn8a/Cacnb2/Cacna1g/Cacna1d/Ank3	6
GO:1903421	regulation of synaptic	6/652	31/28814	5.96E-05	0.00109461	0.0008232	Nlgn1/Dnm3/Gripap1/Pclo/Rock1/Fgf14	6
GO:0007632	visual behavior	9/652	77/28814	6.03E-05	0.00110236	0.000829	Atp1a3/Kmt2a/Zzef1/Atp1a2/Tanc1/Sgk1/Nptn/Atxn1/Grin2a	9
GO:0070588	calcium ion transmembrane transport	19/652	300/28814	6.16E-05	0.00112021	0.0008424	Nalcn/Chd7/Atp1a2/Micu3/Slc24a2/Bin1/Stim1/Stac/Slc8a1/Ryr2/Ryr1/Itpr1/Grin2a/Fgf14/Cacng2/Cacnb4/Cacnb2/Cacna1g/Cacna1d	19
GO:0033674	positive regulation of kinase activity	24/652	435/28814	6.47E-05	0.00117158	0.0008811	Taok3/Ppp1r9a/Fgd4/Nrg1/Ccdc88a/Fbxw7/Tcl1b2/Tenm1/Tlr4/Robo1/Rasgrp1/Ntrk3/Nrxn1/Kitl/Lrp8/Insr/Igf1r/Hsp90aa1/Hsp90ab1/Fgfr2/Erbb4/Epha5/Daxx/Alk	24
GO:0010766	negative regulation of	5/652	20/28814	6.83E-05	0.00122563	0.0009217	Hecw2/Atp1a2/Hecw1/Wnk2/Stk39	5
GO:0032252	secretory granule	5/652	20/28814	6.83E-05	0.00122563	0.0009217	Ppfia2/Tanc2/Rasgrp1/Kif5a/Kif1a	5

GO:0021700	developmental maturation	21/652	355/28814	6.90E-05	0.00123191	0.0009264	Nrg2/Nfasc/Shank1/Slc26a8/Ankrd17/Sptbn4/Srrm4/Rere/Sclt1/Sez6l/Ryr1/Pparg/Ppard/Plcb1/Nrxn1/Kcnma1/Gnaq/Ext1/Erbb4/Camk2b/Ank3	21
GO:0060074	synapse	6/652	32/28814	7.20E-05	0.00127971	0.0009624	Nrg2/Shank1/Sez6l/Nrxn1/Erbb4/Camk2b	6
GO:0032874	positive regulation of stress-	12/652	139/28814	7.96E-05	0.00140902	0.0010596	Taok3/Fgd4/Arl6ip5/Stk39/Mfhas1/Map4k4/Map3k3/Tlr4/Rasgrp1/Plcb1/Hmgb1/Hipk2	12
GO:0032872	regulation of stress-activated MAPK	15/652	207/28814	8.16E-05	0.00143234	0.0010772	Taok3/Fgd4/Arl6ip5/Gps2/Stk39/Mfhas1/Map4k4/Map3k3/Tlr4/Rasgrp1/Plcb1/Igf1r/Hmgb1/Hipk2/Grik2	15
GO:0051965	positive regulation of	9/652	80/28814	8.17E-05	0.00143234	0.0010772	Syndig1/Il1rapl1/Lingo2/Clstn3/Nlgn1/Clstn2/Prkca/Ntrk3/Nrxn1	9
GO:0007215	glutamate receptor	7/652	47/28814	8.43E-05	0.00146513	0.0011018	Kalrn/Atp1a3/Grid2ip/Cpeb4/Plcb1/Grin2a/Gnaq	7
GO:0048489	synaptic vesicle	7/652	47/28814	8.43E-05	0.00146513	0.0011018	Nlgn1/Lin7a/Dnm3/Kif5a/Fgfr2/Dpysl2/Ap3b2	7
GO:0034763	negative regulation of	12/652	140/28814	8.53E-05	0.00147672	0.0011105	Hecw2/Atp1a2/Hecw1/Snx27/Arl6ip5/Stk39/Ahi1/Bin1/Prkca/Pik3c2a/Oaz2/Ank3	12
GO:0044030	regulation of DNA methylation	6/652	33/28814	8.63E-05	0.00148724	0.0011185	Kmt2a/Baz2a/Ftx/Mphosph8/Kcnq1ot1/Parp1	6
GO:0099111	microtubule-based transport	15/652	209/28814	9.09E-05	0.00156032	0.0011734	Nefh/Sfpq/Spag16/Hnrnpu/Rasgrp1/Nefm/Mapt/Map1a/Kif5a/Kif3c/Kif3a/Kif1a/Dst/Bicd1/Ap3b2	15
GO:0070304	positive regulation of stress-	12/652	141/28814	9.14E-05	0.00156085	0.0011738	Taok3/Fgd4/Arl6ip5/Stk39/Mfhas1/Map4k4/Map3k3/Tlr4/Rasgrp1/Plcb1/Hmgb1/Hipk2	12
GO:0021543	pallium development	13/652	163/28814	9.19E-05	0.00156287	0.0011753	Tacc1/Phactr1/Trappc9/Robo1/Plcb1/Afdn/Lrp8/Kif3a/Srgap2/Fgf13/Epha5/Cdh2/Alk	13
GO:0070302	regulation of stress-activated	15/652	210/28814	9.59E-05	0.00162472	0.0012218	Taok3/Fgd4/Arl6ip5/Gps2/Stk39/Mfhas1/Map4k4/Map3k3/Tlr4/Rasgrp1/Plcb1/Igf1r/Hmgb1/Hipk2/Grik2	15

GO:0018209	peptidyl-serine modification	21/652	364/28814	9.81E-05	0.00165497	0.0012446	Egflam/Galnt16/Akap9/Spock2/Sptbn4/Stk39/Tcl1b2/Tenm1/Srpk2/Sgk1/Rock1/Prkca/Ntrk3/Nsd1/Nrxn1/Nlk/Hsp90aa1/Hsp90ab1/Hipk2/Camk2b/Parp1	21
GO:0045055	regulated exocytosis	18/652	287/28814	0.00010786	0.00181097	0.0013619	Ppfia2/Cadps2/Dgki/Nlgn1/Rims2/Erc1/Rab31/Trim9/Snap47/Pclo/Syt2/Syt1/Sptbn2/Rasgrp1/Prkca/Myo6/Cacnb4/Cacna1g	18
GO:0031111	negative regulation of microtubule	7/652	49/28814	0.0001107	0.00184371	0.0013865	Nav3/Atxn7/Camsap2/Hdgfl3/Sgk1/Map1a/Fgf13	7
GO:0007346	regulation of mitotic cell cycle	25/652	479/28814	0.00011075	0.00184371	0.0013865	Taok3/Hecw2/Brinp2/Gigyf2/Ube2e2/Tom1l2/Brinp3/Mta3/Gbf1/Ankrd17/Senp2/Kmt2e/Anp32b/Brd4/Brinp1/Hnrnpu/Plcb1/Prkca/Insr/Igf1r/Hmgb1/Fgfr2/Crebbp/Cdk11b/Cacnb4	25
GO:0071625	vocalization	5/652	22/28814	0.00011177	0.00185291	0.0013934	Shank1/Foxp2/Brinp1/Nrxn1/Ext1	5
GO:2001259	positive regulation of	9/652	84/28814	0.00011967	0.00197555	0.0014857	Akap7/Kcnc2/Stim1/Stac/Fgf14/Cacng2/Cacnb4/Cacnb2/Ank3	9
GO:0032984	protein-containing complex	16/652	239/28814	0.00012155	0.00199262	0.0014985	Setx/Nav3/Atxn7/Nrg1/Mphosph8/Dnajc6/Smarcc2/Camsap2/Hdgfl3/Ssrp1/Sptan1/Sgk1/Map1a/Insr/Igf1r/Fgf13	16
GO:0007026	negative regulation of	6/652	35/28814	0.00012172	0.00199262	0.0014985	Nav3/Atxn7/Camsap2/Hdgfl3/Map1a/Fgf13	6
GO:1901890	positive regulation of	11/652	124/28814	0.00012281	0.00200218	0.0015057	Syndig1/Il1rapl1/Lingo2/Clstn3/Nlgn1/Clstn2/Map4k4/Rock1/Prkca/Ntrk3/Nrxn1	11
GO:0007018	microtubule-based movement	23/652	426/28814	0.00012572	0.0020326	0.0015286	Nefh/Dnah6/Ttll5/Dnah7b/Slc26a8/Armc2/Sfpq/Spag16/Dzip1/Hnrnpu/Rasgrp1/Nefm/Mapt/Map1a/Ktn1/Kif5a/Kif3c/Kif3a/Kif21a/Kif1a/Dst/Bicd1/Ap3b2	23
GO:0007157	heterophilic cell-cell adhesion via	7/652	50/28814	0.00012623	0.0020326	0.0015286	Il1rapl1/Nlgn1/Tenm3/Tenm2/Tenm1/Nrxn1/Cdh2	7
GO:0021988	olfactory lobe development	7/652	50/28814	0.00012623	0.0020326	0.0015286	Chd7/Robo2/Atp1a2/Sema3a/Robo1/Ext1/Erbb4	7
GO:0022037	metencephalon development	11/652	125/28814	0.00013196	0.00209965	0.001579	Herc1/Foxp2/Pdss2/Rere/Sez6l/Sptbn2/Nrxn1/Lhx1/Igf1r/Cntn1/Atrn	11

GO:0030041	actin filament polymerization	13/652	169/28814	0.00013235	0.00209965	0.001579	Ppp1r9a/Fhod3/Kank1/Cyfip2/Mphosph8/Spire1/Diaph2/Bin1/Tenm1/Sptan1/Pstpip2/Pak3/Gas7	13
GO:0006898	receptor-mediated endocytosis	17/652	266/28814	0.00013253	0.00209965	0.001579	Ldlrad3/Nrg1/Rab31/Dnm3/Pard3/Sgip1/Dnajc6/Ap2b1/Ahi1/Itsn2/Prkca/Myo6/Kif3a/Insr/Canx/Cacng2/Bicd1	17
GO:1903522	regulation of blood circulation	17/652	266/28814	0.00013253	0.00209965	0.001579	Tnni3k/Ctnna3/Akap9/Atp1a2/Rbfox2/Sptbn4/Bin1/Slc8a1/Slc4a3/Sema3a/Ryr2/Prkca/Gnao1/Fgf13/Cacnb2/Cacna1g/Cacna1d	17
GO:0048015	phosphatidylinositol-mediated signaling	13/652	170/28814	0.0001404	0.00221535	0.001666	Nrg1/Rasgrp1/Ptpn13/Ppard/Plcb4/Plcb3/Plcb1/Pik3c2a/Ntrk3/Insr/Igf1r/Erbb4/Car8	13
GO:0008038	neuron recognition	7/652	51/28814	0.00014348	0.00223935	0.0016841	Robo2/Sema3a/Nptn/Robo1/Ncam1/Ext1/Dscam	7
GO:0061647	histone H3-K9 modification	7/652	51/28814	0.00014348	0.00223935	0.0016841	Kmt2a/Baz2a/Arid4b/Hdac8/Rif1/Atrx/Kat2b	7
GO:0046785	microtubule polymerization	9/652	86/28814	0.00014363	0.00223935	0.0016841	Nav3/Akap9/Pde4dip/Camsap2/Clip1/Hdgfl3/Sgk1/Mapt/Fgf13	9
GO:0006304	DNA modification	10/652	106/28814	0.000149	0.00231157	0.0017384	Kmt2a/Baz2a/Ftx/Ascc3/Mphosph8/Kmt2e/Kcnq1ot1/Atf7ip/Meg3/Parp1	10
GO:0009416	response to light stimulus	19/652	321/28814	0.00014944	0.00231157	0.0017384	Atp1a3/Kmt2a/Zzef1/Atp1a2/Slc4a10/Slc24a2/Tanc1/Fbxl17/Fbxw7/Mapk10/Sgk1/Nptn/Atxn1/Pparg/Grin2a/Ext1/Crebbp/Cacnb4/Parp1	19
GO:0048839	inner ear development	15/652	219/28814	0.00015277	0.00235378	0.0017701	Chd7/Plppr4/Lin7a/Anp32b/Ahi1/Esrrb/Ush2a/Zeb1/Myo6/Kif3a/Kcnma1/Gabrb2/Fgfr2/Col2a1/Pcdh15	15
GO:0065004	protein-DNA complex	12/652	149/28814	0.00015455	0.00237203	0.0017838	Bdp1/Taf1/Taf3/Anp32b/Smarca2/Atf7ip/Kat6b/Sart3/Atrx/Hp1bp3/Hmgb1/Daxx	12
GO:0034766	negative regulation of	10/652	107/28814	0.00016108	0.00246262	0.001852	Hecw2/Atp1a2/Hecw1/Snx27/Arl6ip5/Stk39/Bin1/Prkca/Pik3c2a/Ank3	10
GO:0045807	positive regulation of	11/652	128/28814	0.00016295	0.00248157	0.0018662	Nlgn1/Rab31/Pard3/Sgip1/Ap2b1/Ahi1/Bin1/Pparg/Kif3a/Insr/Bicd1	11

GO:0048017	inositol lipid-mediated signaling	13/652	173/28814	0.00016714	0.00252571	0.0018994	Nrg1/Rasgrp1/Ptpn13/Ppard/Plcb4/Plcb3/Plcb1/Pik3c2a/Ntrk3/Insr/Igf1r/Erbb4/Car8	13
GO:0051017	actin filament bundle assembly	13/652	173/28814	0.00016714	0.00252571	0.0018994	Shank1/Ppp1r9a/Phactr1/Ccdc88a/Limch1/Arap1/Spire1/Tmeff2/Espn/Dlc1/Hsp90b1/Gas7/Pcdh15	13
GO:0006473	protein acetylation	15/652	221/28814	0.00016877	0.00253093	0.0019033	Taf1/Atxn7/Phf20l1/Kmt2a/Kansl1/Naa15/Setd5/Hdac8/Naa16/Brd4/Kat6b/Kat2b/Ncoa1/Crebbp/Arntl	15
GO:0007623	circadian rhythm	15/652	221/28814	0.00016877	0.00253093	0.0019033	Setx/Kmt2a/Nlgn1/Mycbp2/Sfpq/Hnrnpu/Fbxl17/Fbxw7/Mapk10/Pparg/Ntrk3/Kcnma1/Zfhx3/Arntl/Alb	15
GO:0010842	retina layer	5/652	24/28814	0.00017377	0.00258616	0.0019449	Sdk1/Ahi1/Lhx1/Hipk2/Calb1	5
GO:0098901	regulation of cardiac muscle	5/652	24/28814	0.00017377	0.00258616	0.0019449	Ctnna3/Akap9/Bin1/Ryr2/Fgf13	5
GO:0046328	regulation of JNK cascade	12/652	152/28814	0.00018639	0.00276346	0.0020782	Taok3/Fgd4/Gps2/Mfhas1/Map4k4/Tlr4/Rasgrp1/Plcb1/Igf1r/Hmgb1/Hipk2/Grik2	12
GO:0045787	positive regulation of cell cycle	20/652	355/28814	0.00019532	0.00286534	0.0021548	Eif4g3/Pkp4/Ube2e2/Mta3/Ankrd17/Sfpq/Kmt2e/Anp32b/Brd4/Hnrnpu/Atrx/Srpk2/Plcb1/Prkca/Insr/Igf1r/Hmgb1/Fgfr2/Crebbp/Camk2b	20
GO:0031114	regulation of microtubule	6/652	38/28814	0.00019545	0.00286534	0.0021548	Nav3/Atxn7/Camsap2/Hdgfl3/Map1a/Fgf13	6
GO:0086004	regulation of cardiac muscle	6/652	38/28814	0.00019545	0.00286534	0.0021548	Ctnna3/Akap9/Atp1a2/Bin1/Ryr2/Fgf13	6
GO:0061572	actin filament bundle organization	13/652	176/28814	0.00019814	0.00289404	0.0021764	Shank1/Ppp1r9a/Phactr1/Ccdc88a/Limch1/Arap1/Spire1/Tmeff2/Espn/Dlc1/Hsp90b1/Gas7/Pcdh15	13
GO:0051592	response to calcium ion	11/652	131/28814	0.00019992	0.00290921	0.0021878	Fus/Nlgn1/Cpne8/Spag16/Syt2/Syt1/Stim1/Ryr2/Pde1c/Nrxn1/Kcnma1	11
GO:0007019	microtubule depolymerizatio	7/652	54/28814	0.00020701	0.00300128	0.0022571	Nav3/Atxn7/Camsap2/Hdgfl3/Sgk1/Map1a/Fgf13	7
GO:0043244	regulation of protein-containing	11/652	132/28814	0.00021372	0.00307587	0.0023132	Setx/Nav3/Atxn7/Mphosph8/Camsap2/Hdgfl3/Sptan1/Map1a/Insr/Igf1r/Fgf13	11

GO:0060048	cardiac muscle contraction	11/652	132/28814	0.00021372	0.00307587	0.0023132	Atp1a3/Ctnna3/Akap9/Atp1a2/Bin1/Slc8a1/Ryr2/Fgf13/Cacnb2/Cacna1g/Cacna1d	11
GO:0030004	cellular monovalent	10/652	111/28814	0.00021801	0.00312608	0.0023509	Atp6ap1l/Atp1a3/Atp1a2/Slc4a10/Slc12a5/Slc4a4/Slc8a1/Slc4a3/Sgk1/Kcnma1	10
GO:0048168	regulation of neuronal	8/652	72/28814	0.00022043	0.00314938	0.0023684	Kalrn/Kmt2a/Slc4a10/Syn1/Nptn/Grin2a/Grik2/Camk2b	8
GO:0021680	cerebellar Purkinje cell	6/652	39/28814	0.0002266	0.00322577	0.0024259	Herc1/Foxp2/Rere/Sez6l/Sptbn2/Lhx1	6
GO:0007062	sister chromatid	7/652	55/28814	0.00023262	0.00329953	0.0024814	Pds5b/Sfpq/Hdac8/Fbxw7/Atrx/Stag1/Smc3	7
GO:0021987	cerebral cortex development	10/652	112/28814	0.00023461	0.0033158	0.0024936	Tacc1/Phactr1/Trappc9/Robo1/Plcb1/Afdn/Lrp8/Srgap2/Fgf13/Cdh2	10
GO:0120032	regulation of plasma membrane bounded cell	14/652	205/28814	0.00025846	0.00363979	0.0027372	Mphosph9/Ppp1r9a/Gpm6a/Bcas3/Nlgn1/Ccdc88a/Kank1/Dnm3/Arap1/Dzip1/Espn/Tenm1/Nrxn1/Myo10	14
GO:0044728	DNA methylation or	9/652	93/28814	0.00026124	0.00366577	0.0027568	Kmt2a/Baz2a/Ftx/Mphosph8/Kmt2e/Kcnq1ot1/Atf7ip/Meg3/Parp1	9
GO:0019932	second-messenger-mediated	18/652	309/28814	0.00026838	0.00375258	0.0028221	Kcnc2/Ppp1r9a/Nrg1/Camta1/Cap2/Pde10a/Tenm2/Slc8a1/Exoc4/Ryr2/Prkg1/Pde9a/Ncam1/Mapt/Itpr1/Epha5/Cdh13/Cacna1d	18
GO:1901214	regulation of neuron death	21/652	392/28814	0.00027047	0.00376837	0.0028339	Ncoa7/Trim2/Ppp2r2b/Ap2b1/Cpeb4/Fbxw7/Tlr4/Srpk2/Rock1/Pak3/Mapt/Kcnma1/Itpr1/Hsp90ab1/Hipk2/Grik2/Gabrb2/Dcc/Daxx/Cntfr/Parp1	21
GO:0036376	sodium ion export across	4/652	15/28814	0.00029066	0.0039933	0.0030031	Atp1a3/Atp1a2/Slc4a4/Slc8a1	4
GO:1901387	positive regulation of voltage-gated	4/652	15/28814	0.00029066	0.0039933	0.0030031	Stac/Fgf14/Cacnb4/Cacnb2	4
GO:1902306	negative regulation of	4/652	15/28814	0.00029066	0.0039933	0.0030031	Hecw2/Atp1a2/Hecw1/Stk39	4
GO:0032956	regulation of actin cytoskeleton organization	20/652	366/28814	0.00029068	0.0039933	0.0030031	Shank1/Ppp1r9a/Fhod3/Bcas3/Ccdc88a/Kank1/Limch1/Cyfip2/Mphosph8/Arap1/Tmeff2/Dlc1/Bin1/Tenm1/Sptan1/Rock1/Pak3/Ntrk3/Epha5/Ctnna2	20

GO:0060491	regulation of cell projection assembly	14/652	208/28814	0.00029984	0.00407206	0.0030623	Mphosph9/Ppp1r9a/Gpm6a/Bcas3/Nlgn1/Ccdc88a/Kank1/Dnm3/Arap1/Dzip1/Espn/Tenm1/Nrxn1/Myo10	14
GO:0043267	negative regulation of	6/652	41/28814	0.00030056	0.00407206	0.0030623	Snx27/Stk39/Bin1/Plcb4/Gnaq/Ank3	6
GO:0086065	cell communication	6/652	41/28814	0.00030056	0.00407206	0.0030623	Ctnna3/Slc8a1/Ryr2/Cacnb2/Cacna1g/Cacna1d	6
GO:2000463	positive regulation of excitatory	6/652	41/28814	0.00030056	0.00407206	0.0030623	Shank1/Nlgn1/Rims2/Nrxn1/Afdn/Grin2a	6
GO:0033002	muscle cell proliferation	16/652	259/28814	0.00030276	0.00408781	0.0030742	Kalrn/Nrg1/Paxbp1/Zfpm2/Wnt2/Tlr4/Prkg1/Pparg/Ppard/Prkca/Ncam1/Igf1r/Gnaq/Fgfr2/Erbb4/Cdh13	16
GO:0006376	mRNA splice	5/652	27/28814	0.00031206	0.00419888	0.0031577	Setx/Sfswap/Luc7l2/Psip1/Luc7l	5
GO:0043666	regulation of phosphoprotein	7/652	58/28814	0.00032511	0.00435956	0.0032785	Ppp4r4/Ppp2r2b/Mprip/Hsp90b1/Rock1/Ppp1r12a/Hsp90ab1	7
GO:0051588	regulation of neurotransmitte	11/652	139/28814	0.00033479	0.00447417	0.0033647	Ppfia2/Dgki/Ppp1r9a/Nlgn1/Rims2/Mctp1/Arl6ip5/Syt1/Prkca/Myo6/Cacnb4	11
GO:0006029	proteoglycan metabolic	8/652	77/28814	0.00035112	0.00466061	0.0035049	Hs3st5/Egflam/Spock2/Chst8/Hs6st3/Ppard/Ext1/Col2a1	8
GO:0043113	receptor clustering	8/652	77/28814	0.00035112	0.00466061	0.0035049	Gphn/Lrrc7/Sorbs2/Nlgn1/Snx27/Nrxn1/Grik2/Cdh2	8
GO:0048872	homeostasis of number of cells	20/652	372/28814	0.00035819	0.00472214	0.0035512	Cadps2/Gigyf2/Kmt2a/Dock10/Exoc6/Rbfox2/Kmt2e/Smarca2/Sart3/Mfhas1/Sox6/Pol1b/Plcb1/Afdn/Kitl/Kif3a/Hmgb1/Ext1/Cdh2/Ank1	20
GO:0043543	protein acylation	16/652	263/28814	0.00035884	0.00472214	0.0035512	Taf1/Atxn7/Phf20l1/Kmt2a/Kansl1/Naa15/Setd5/Hdac8/Naa16/Brd4/Kat6b/Kat2b/Nmt2/Ncoa1/Crebbp/Arntl	16
GO:0007528	neuromuscular junction	7/652	59/28814	0.00036176	0.00472214	0.0035512	Kalrn/Gphn/Sorbs2/Fgfr2/Cacng2/Cacnb4/Cacnb2	7
GO:0031113	regulation of microtubule	7/652	59/28814	0.00036176	0.00472214	0.0035512	Nav3/Akap9/Pde4dip/Camsap2/Clip1/Sgk1/Mapt	7
GO:0050775	positive regulation of	7/652	59/28814	0.00036176	0.00472214	0.0035512	Kalrn/Il1rapl1/Sgk1/Pak3/Afdn/Lrp8/Camk2b	7

GO:0031589	cell-substrate adhesion	20/652	373/28814	0.00037067	0.00482244	0.0036266	Egflam/Fat2/Atrnl1/Bcas3/Kank1/Spock2/Ntng1/Limch1/Vit/Fndc3b/Tmeff2/Dlc1/Map4k4/Ush2a/Rock1/Ppard/Srgap2/Edil3/Cdh13/Atrn	20
GO:0010765	positive regulation of	6/652	43/28814	0.00039222	0.00503607	0.0037873	Wnk2/Sgk1/Plcb1/Fgf14/Cntn1/Ank3	6
GO:0086002	cardiac muscle cell action potential	6/652	43/28814	0.00039222	0.00503607	0.0037873	Ctnna3/Bin1/Ryr2/Cacnb2/Cacna1g/Cacna1d	6
GO:0097484	dendrite	6/652	43/28814	0.00039222	0.00503607	0.0037873	Rims2/Cyfip2/Syt2/Syt1/Itsn2/Afdn	6
GO:2000008	regulation of protein	6/652	43/28814	0.00039222	0.00503607	0.0037873	Nrg1/Gbf1/Astn2/Map1a/Hsp90ab1/Erbb4	6
GO:0008361	regulation of cell size	14/652	214/28814	0.00039984	0.00511722	0.0038483	Fstl4/Plxna4/Nrg1/Slc12a5/Golga4/Sema3a/Ntrk3/Mapt/Kcnma1/Hsp90ab1/Fgf13/Dscam/Dpysl2/Arhgap5	14
GO:0098815	modulation of excitatory	7/652	60/28814	0.00040165	0.00512366	0.0038532	Shank1/Nlgn1/Rims2/Pclo/Nrxn1/Afdn/Grin2a	7
GO:0060541	respiratory system development	16/652	267/28814	0.00042365	0.00536942	0.004038	Chd7/Foxp2/Foxp4/Fndc3b/Fbxw7/Abca3/Zfpm2/Wnt2/Atxn1/Myt1/Meg3/Man1a2/Smad2/Hmgb1/Fgfr2/Ext1	16
GO:0090068	positive regulation of cell cycle	16/652	267/28814	0.00042365	0.00536942	0.004038	Eif4g3/Pkp4/Ube2e2/Mta3/Ankrd17/Sfpq/Kmt2e/Anp32b/Brd4/Hnrnpu/Atrx/Plcb1/Insr/Igf1r/Crebbp/Camk2b	16
GO:0045664	regulation of neuron differentiation	15/652	241/28814	0.00042715	0.00539641	0.0040583	Cntn4/Brinp2/Brinp3/Nlgn1/Spag9/Brinp1/Bin1/Ttc3/Zeb1/Sox5/Rock1/Ncoa1/Dpysl2/Zfhx3/Alk	15
GO:0010720	positive regulation of cell development	20/652	378/28814	0.00043898	0.00545351	0.0041012	Kalrn/Il1rapl1/Robo2/Plxna4/Nrg1/Golga4/Bin1/Wnt2/Sgk1/Nptn/Atxn1/Robo1/Pparg/Pak3/Ntrk3/Mapt/Afdn/Lrp8/Dscam/Camk2b	20
GO:0061003	positive regulation of	5/652	29/28814	0.00044222	0.00545351	0.0041012	Kalrn/Pak3/Afdn/Lrp8/Camk2b	5
GO:0099637	neurotransmitter receptor	5/652	29/28814	0.00044222	0.00545351	0.0041012	Grip1/Snap47/Gripap1/Mapk10/Cacng2	5
GO:1903861	positive regulation of	5/652	29/28814	0.00044222	0.00545351	0.0041012	Rims2/Syt2/Syt1/Itsn2/Afdn	5

GO:0016573	histone acetylation	12/652	167/28814	0.00044272	0.00545351	0.0041012	Taf1/Atxn7/Phf20l1/Kmt2a/Kansl1/Setd5/Hdac8/Brd4/Kat6b/Kat2b/Ncoa1/Crebbp	12
GO:0040029	regulation of gene expression,	12/652	167/28814	0.00044272	0.00545351	0.0041012	Smyd5/Baz2a/Arid4b/Ftx/Kansl1/Mphosph8/Fam172a/Kcnq1ot1/Atf7ip/Rif1/Hnrnpu/Meg3	12
GO:0042551	neuron maturation	7/652	61/28814	0.00044498	0.00545351	0.0041012	Nfasc/Sptbn4/Srrm4/Sclt1/Nrxn1/Gnaq/Ank3	7
GO:0000245	spliceosomal	6/652	44/28814	0.00044555	0.00545351	0.0041012	Setx/Sfswap/Luc7l2/Psip1/Luc7l/Srpk2	6
GO:0010464	regulation of mesenchymal	6/652	44/28814	0.00044555	0.00545351	0.0041012	Foxp2/Wnt2/Zeb1/Hmgb1/Fgfr2/Arhgap5	6
GO:1901021	positive regulation of calcium ion	6/652	44/28814	0.00044555	0.00545351	0.0041012	Stim1/Stac/Ryr2/Fgf14/Cacnb4/Cacnb2	6
GO:0046928	regulation of neurotransmitte	10/652	122/28814	0.00046735	0.0057025	0.0042885	Ppfia2/Dgki/Ppp1r9a/Nlgn1/Rims2/Mctp1/Syt1/Prkca/Myo6/Cacnb4	10
GO:0035235	ionotropic glutamate	4/652	17/28814	0.00048886	0.00590999	0.0044445	Kalrn/Atp1a3/Cpeb4/Grin2a	4
GO:0086012	membrane depolarization during cardiac	4/652	17/28814	0.00048886	0.00590999	0.0044445	Cacnb2/Cacna1g/Cacna1d/Ank3	4
GO:1990806	ligand-gated ion channel	4/652	17/28814	0.00048886	0.00590999	0.0044445	Kalrn/Atp1a3/Cpeb4/Grin2a	4
GO:0035176	social behavior	7/652	62/28814	0.00049195	0.00592909	0.0044589	Kalrn/Shank1/Chd8/Brinp1/Atxn1/Nrxn1/Ext1	7
GO:0043279	response to alkaloid	8/652	81/28814	0.00049602	0.00594167	0.0044683	Kalrn/Sdk1/Slc8a1/Ryr2/Ryr1/Ncam1/Crebbp/Parp1	8
GO:1901880	negative regulation of	8/652	81/28814	0.00049602	0.00594167	0.0044683	Nav3/Atxn7/Mphosph8/Camsap2/Hdgfl3/Sptan1/Map1a/Fgf13	8
GO:0051446	positive regulation of	5/652	30/28814	0.00052087	0.00622032	0.0046779	Eif4g3/Plcb1/Insr/Igf1r/Camk2b	5
GO:0014855	striated muscle cell	8/652	82/28814	0.00053893	0.00641649	0.0048254	Nrg1/Paxbp1/Zfpm2/Wnt2/Ppard/Ncam1/Fgfr2/Erbb4	8
GO:0017157	regulation of exocytosis	15/652	247/28814	0.00055203	0.00655261	0.0049278	Il1rapl1/Ppfia2/Cadps2/Dgki/Nlgn1/Rims2/Trim9/Pclo/Syt2/Syt1/Prkca/Myo6/Cacnb4/Cacna1g/Atp9a	15

GO:0051261	protein depolymerizatio	10/652	125/28814	0.00056631	0.00667409	0.0050191	Nav3/Atxn7/Mphosph8/Dnajc6/Camsap2/Hdgfl3/Sptan1/Sgk1/Map1a/Fgf13	10
GO:0055023	positive regulation of	6/652	46/28814	0.00056906	0.00667409	0.0050191	Nrg1/Zfpm2/Wnt2/Ncam1/Fgfr2/Erbb4	6
GO:1900087	positive regulation of G1/S transition	6/652	46/28814	0.00056906	0.00667409	0.0050191	Ube2e2/Ankrd17/Kmt2e/Anp32b/Plcb1/Crebbp	6
GO:1903115	regulation of actin filament-	6/652	46/28814	0.00056906	0.00667409	0.0050191	Ctnna3/Akap9/Atp1a2/Bin1/Ryr2/Fgf13	6
GO:0045860	positive regulation of protein kinase activity	19/652	358/28814	0.00058224	0.00680836	0.0051201	Taok3/Ppp1r9a/Fgd4/Nrg1/Ccdc88a/Fbxw7/Tcl1b2/Tenm1/Tlr4/Robo1/Rasgrp1/Ntrk3/Nrxn1/Kitl/Lrp8/Insr/Hsp90aa1/Hsp90ab1/Daxx	19
GO:1901016	regulation of potassium ion transmembrane	7/652	64/28814	0.00059773	0.00696876	0.0052407	Akap7/Kcnc2/Akap9/Snx27/Stk39/Cacna1d/Ank3	7
GO:0110053	regulation of actin filament organization	16/652	276/28814	0.00060721	0.00705835	0.0053081	Shank1/Ppp1r9a/Fhod3/Ccdc88a/Kank1/Limch1/Cyfip2/Mphosph8/Arap1/Tmeff2/Dlc1/Bin1/Tenm1/Sptan1/Pak3/Ctnna2	16
GO:0021952	central nervous system projection	5/652	31/28814	0.00060957	0.00706487	0.005313	Plxna4/Adarb1/Mycbp2/Sptbn4/Dcc	5
GO:0008154	actin polymerization or	13/652	198/28814	0.00061533	0.0070922	0.0053336	Ppp1r9a/Fhod3/Kank1/Cyfip2/Mphosph8/Spire1/Diaph2/Bin1/Tenm1/Sptan1/Pstpip2/Pak3/Gas7	13
GO:0006474	N-terminal protein amino	4/652	18/28814	0.00061734	0.0070922	0.0053336	Naa15/Naa16/Kat2b/Crebbp	4
GO:0032793	positive regulation of	4/652	18/28814	0.00061734	0.0070922	0.0053336	Lrp8/Epha5/Crebbp/Cacna1d	4
GO:0009100	glycoprotein metabolic process	18/652	332/28814	0.00062832	0.00719731	0.0054126	Hs3st5/Tmtc2/Egflam/Manba/Galnt16/Slc4a10/Spock2/Chst8/Mgat4c/Uggt2/Hs6st3/Ppard/Plcb1/Pcsk6/Man1a2/Insr/Ext1/Col2a1	18
GO:0006260	DNA replication	16/652	277/28814	0.00063128	0.00721014	0.0054223	Zbtb38/Ccdc88a/Fancm/Nucks1/Ankrd17/Senp2/Rbms1/Ddx21/Atrx/Ssrp1/Srpk2/Rbbp6/Polb/Fhit/Dach1/Smc3	16

GO:2000300	regulation of synaptic	8/652	84/28814	0.00063374	0.00721734	0.0054277	Ppfia2/Dgki/Nlgn1/Rims2/Syt1/Prkca/Myo6/Cacnb4	8
GO:0006941	striated muscle contraction	12/652	174/28814	0.00063914	0.00724656	0.0054497	Atp1a3/Ctnna3/Akap9/Atp1a2/Bin1/Stac/Slc8a1/Ryr2/Fgf13/Cacnb2/Cacna1g/Cacna1d	12
GO:0021772	olfactory bulb	6/652	47/28814	0.00064	0.00724656	0.0054497	Chd7/Robo2/Sema3a/Robo1/Ext1/Erbb4	6
GO:0046330	positive regulation of	9/652	105/28814	0.00064271	0.00725635	0.005457	Taok3/Fgd4/Mfhas1/Map4k4/Tlr4/Rasgrp1/Plcb1/Hmgb1/Hipk2	9
GO:0043583	ear development	15/652	251/28814	0.00065156	0.0073352	0.0055163	Chd7/Plppr4/Lin7a/Anp32b/Ahi1/Esrrb/Ush2a/Zeb1/Myo6/Kif3a/Kcnma1/Gabrb2/Fgfr2/Col2a1/Pcdh15	15
GO:0051703	biological process involved in	7/652	65/28814	0.00065698	0.0073751	0.0055463	Kalrn/Shank1/Chd8/Brinp1/Atxn1/Nrxn1/Ext1	7
GO:0061387	regulation of extent of cell	10/652	128/28814	0.00068201	0.0076342	0.0057412	Fstl4/Plxna4/Nrg1/Golga4/Sema3a/Ntrk3/Mapt/Fgf13/Dscam/Dpysl2	10
GO:0072384	organelle transport along	8/652	85/28814	0.00068596	0.00765661	0.005758	Nefh/Rasgrp1/Mapt/Kif5a/Kif3c/Kif1a/Bicd1/Ap3b2	8
GO:0050806	positive regulation of synaptic transmission	18/652	335/28814	0.0006972	0.00776005	0.0058358	Kalrn/Mir124a-1hg/Ppp1r9a/Clstn3/Nlgn1/Rims2/Slc24a2/Snap47/Clstn2/Syt1/Nptn/Nrxn1/Grin2a/Grik2/Erbb4/Camk2b/Calb1/Cacng2	18
GO:0007605	sensory perception of sound	16/652	280/28814	0.00070844	0.00784827	0.0059022	Chd7/Mir124a-1hg/Sptbn4/Srrm4/Ccdc50/Fam107b/Espn/Ush2a/Scn8a/Myo6/Map1a/Kcnma1/Gabrb2/Col2a1/Cacna1d/Pcdh15	16
GO:1903859	regulation of dendrite	5/652	32/28814	0.00070912	0.00784827	0.0059022	Rims2/Syt2/Syt1/Itsn2/Afdn	5
GO:0001941	postsynaptic membrane	6/652	48/28814	0.00071761	0.00791987	0.005956	Gphn/Sorbs2/Nlgn1/Snx27/Nrxn1/Cdh2	6
GO:0007517	muscle organ development	19/652	365/28814	0.00073427	0.00808113	0.0060773	Chd7/Chd2/Nrg1/Foxp2/Adarb1/Paxbp1/Luc7l/Zfpm2/Wnt2/Scn8a/Ryr2/Ryr1/Myt1/Meg3/Fgfr2/Col19a1/Cntfr/Zfhx3/Arntl	19
GO:0006475	internal protein amino	12/652	177/28814	0.0007433	0.00813484	0.0061177	Taf1/Atxn7/Phf20l1/Kmt2a/Kansl1/Setd5/Hdac8/Brd4/Kat6b/Kat2b/Ncoa1/Crebbp	12

GO:0018393	internal peptidyl-lysine	12/652	177/28814	0.0007433	0.00813484	0.0061177	Taf1/Atxn7/Phf20l1/Kmt2a/Kansl1/Setd5/Hdac8/Brd4/Kat6b/Kat2b/Ncoa1/Crebbp	12
GO:0070646	protein modification by	11/652	153/28814	0.00075321	0.00822047	0.0061821	Atxn7/Usp35/Otud7a/Usp8/Senp2/Usp16/Senp7/Usp29/Sart3/Desi1/Usp34	11
GO:0048608	reproductive structure development	23/652	484/28814	0.00075682	0.00823691	0.0061944	Nefh/Chd7/Safb2/Jmjd1c/Akap9/Arid4b/Csmd1/Senp2/Esrrb/Zfpm2/Atrx/Wnt2/Slc8a1/Sema3a/Pparg/Ppard/Ncoa1/Kitl/Lhx1/Insr/Igf1r/Hsp90ab1/Fgfr2	23
GO:0045292	mRNA cis splicing, via	4/652	19/28814	0.00076804	0.00833602	0.006269	Sfswap/Psip1/Prpf40a/Prpf4b	4
GO:0050954	sensory perception of mechanical stimulus	17/652	310/28814	0.00077535	0.00839216	0.0063112	Chd7/Mir124a-1hg/Sptbn4/Srrm4/Ccdc50/Fam107b/Espn/Ush2a/Tlr4/Scn8a/Myo6/Map1a/Kcnma1/Gabrb2/Col2a1/Cacna1d/Pcdh15	17
GO:0007163	establishment or maintenance of cell	14/652	229/28814	0.00078148	0.00843524	0.0063436	Myo18a/Lrrc7/Bcas3/Lin7a/Kank1/Gbf1/Pard3/Exoc4/Kif3a/Igf1r/Hsp90aa1/Hsp90ab1/Fgf13/Dst	14
GO:0090257	regulation of muscle system process	15/652	256/28814	0.0007971	0.00856576	0.0064417	Ctnna3/Akap9/Atp1a2/Bin1/Slc8a1/Ryr2/Ryr1/Rock1/Prkg1/Pparg/Prkca/Pde9a/Kcnma1/Fgf13/Parp1	15
GO:0032410	negative regulation of	8/652	87/28814	0.00080078	0.00856576	0.0064417	Hecw2/Atp1a2/Hecw1/Snx27/Stk39/Prkca/Insr/Ank3	8
GO:0019228	neuronal action	6/652	49/28814	0.00080229	0.00856576	0.0064417	Scn2a/Scn8a/Kcnma1/Grik2/Cacna1g/Ank3	6
GO:0060421	positive regulation of	6/652	49/28814	0.00080229	0.00856576	0.0064417	Nrg1/Zfpm2/Wnt2/Ncam1/Fgfr2/Erbb4	6
GO:0071559	response to transforming growth factor	14/652	230/28814	0.00081527	0.00867068	0.0065206	Zeb1/Sox6/Sox5/Glg1/Rock1/Pparg/Nlk/Smad2/Igf1r/Hsp90ab1/Hipk2/Crebbp/Zfhx3/Parp1	14
GO:1901216	positive regulation of	10/652	131/28814	0.00081653	0.00867068	0.0065206	Ppp2r2b/Fbxw7/Tlr4/Srpk2/Pak3/Mapt/Kcnma1/Grik2/Daxx/Parp1	10
GO:0022411	cellular component disassembly	21/652	427/28814	0.00082172	0.00870008	0.0065427	Setx/Nav3/Atxn7/Nrg1/Gbf1/Lrba/Mphosph8/Dnajc6/Smarcc2/Camsap2/Akap8l/Fbxw7/Hdgfl3/Map4k4/Ssrp1/Sptan1/Sgk1/Map1a/Insr/Igf1r/Fgf13	21

GO:0003007	heart morphogenesis	16/652	284/28814	0.00082373	0.00870008	0.0065427	Chd7/Robo2/Nrg1/Ahi1/Dlc1/Zfpm2/Wnt2/Slc8a1/Ryr2/Ryr1/Robo1/Kif3a/Insr/Fgfr2/Ext1/Col2a1	16
GO:0090150	establishment of protein localization to	15/652	257/28814	0.0008293	0.00873544	0.0065693	Rab31/Pard3/Snx27/Grip1/Snap47/Srp72/Clip1/Gripap1/Golga4/Mapk10/Exoc4/Afdn/Hsp90aa1/Cacng2/Ank3	15
GO:0061458	reproductive system development	23/652	488/28814	0.00084492	0.00887613	0.0066751	Nefh/Chd7/Safb2/Jmjd1c/Akap9/Arid4b/Csmd1/Senp2/Esrrb/Zfpm2/Atrx/Wnt2/Slc8a1/Sema3a/Pparg/Ppard/Ncoa1/Kitl/Lhx1/Insr/Igf1r/Hsp90ab1/Fgfr2	23
GO:0061951	establishment of protein localization to	7/652	68/28814	0.00086311	0.00904304	0.0068007	Rab31/Snx27/Grip1/Gripap1/Golga4/Afdn/Ank3	7
GO:0000082	G1/S transition of mitotic cell cycle	13/652	206/28814	0.00088884	0.00928793	0.0069848	Gigyf2/Ube2e2/Ankrd17/Senp2/Kmt2e/Anp32b/Usp29/Brd4/Esrrb/Plcb1/Crebbp/Camk2b/Cacnb4	13
GO:0003012	muscle system process	21/652	430/28814	0.00089737	0.00935211	0.0070331	Sorbs2/Atp1a3/Ctnna3/Akap9/Atp1a2/Bin1/Stac/Slc8a1/Ryr2/Ryr1/Rock1/Prkg1/Pparg/Prkca/Pde9a/Kcnma1/Fgf13/Cacnb2/Cacna1g/Cacna1d/Parp1	21
GO:0007264	small GTPase mediated signal transduction	21/652	431/28814	0.00092388	0.00957763	0.0072027	Kalrn/Rasgef1b/Dgki/Dennd1a/Cdc42bpa/Nrg1/Dock10/Ccdc88a/Kank1/Dennd4a/Usp8/Arhgap26/Dlc1/Map4k4/Rock1/Robo1/Rasgrp1/Kitl/Cdh2/Cdh13/Arhgap31	21
GO:0009314	response to radiation	21/652	431/28814	0.00092388	0.00957763	0.0072027	Atp1a3/Kmt2a/Zzef1/Atp1a2/Nucks1/Slc4a10/Slc24a2/Tanc1/Fbxl17/Fbxw7/Mapk10/Sgk1/Nptn/Atxn1/Pparg/Nek1/Grin2a/Ext1/Crebbp/Cacnb4/Parp1	21
GO:0008360	regulation of cell shape	11/652	157/28814	0.00093184	0.00963474	0.0072456	Strip2/Plxna4/Fgd4/Dnmbp/Fmnl2/Arap1/Prpf40a/Dlc1/Myo10/Kif3a/Gas7	11
GO:0021695	cerebellar cortex	7/652	69/28814	0.0009421	0.00968453	0.0072831	Herc1/Foxp2/Rere/Sez6l/Sptbn2/Nrxn1/Lhx1	7
GO:0098969	neurotransmitter receptor transport to	4/652	20/28814	0.00094298	0.00968453	0.0072831	Grip1/Snap47/Gripap1/Mapk10	4

GO:0031116	positive regulation of	5/652	34/28814	0.00094405	0.00968453	0.0072831	Nav3/Akap9/Pde4dip/Clip1/Mapt	5
GO:0032869	cellular response to insulin	13/652	208/28814	0.00097124	0.00989516	0.0074415	Tbc1d4/Kank1/Rab31/Nucks1/Sgk1/Ptpre/Pparg/Plcb1/Prkca/Kat2b/Insr/Igf1r/Parp1	13
GO:0035303	regulation of dephosphorylati	10/652	134/28814	0.00097214	0.00989516	0.0074415	Camta1/Ppp4r4/Ppp2r2b/Mfhas1/Dlc1/Mprip/Hsp90b1/Rock1/Ppp1r12a/Hsp90ab1	10
GO:0045931	positive regulation of	10/652	134/28814	0.00097214	0.00989516	0.0074415	Ube2e2/Mta3/Ankrd17/Kmt2e/Anp32b/Brd4/Plcb1/Prkca/Hmgb1/Crebbp	10
GO:0007548	sex differentiation	17/652	317/28814	0.00099063	0.01005732	0.0075634	Nefh/Chd7/Mir124a-1hg/Safb2/Jmjd1c/Akap9/Arid4b/Csmd1/Dach2/Zfpm2/Atrx/Sema3a/Kitl/Lhx1/Insr/Dach1/Cntfr	17
GO:0043268	positive regulation of	6/652	51/28814	0.00099461	0.01007174	0.0075743	Akap7/Kcnc2/Akap9/Wnk2/Stk39/Rgs7	6
GO:0051402	neuron apoptotic process	17/652	318/28814	0.00102519	0.01034321	0.0077784	Scn2a/Adarb1/Trim2/Ppp2r2b/Cpeb4/Fbxw7/Srpk2/Rock1/Polb/Pak3/Kcnma1/Hsp90ab1/Hipk2/Grik2/Gabrb2/Cntfr/Parp1	17
GO:0015800	acidic amino acid transport	7/652	70/28814	0.00102669	0.01034321	0.0077784	Lrrc8c/Arl6ip5/Gabbr1/Slc6a6/Slc1a6/Myo6/Dpysl2	7
GO:0030324	lung development	14/652	236/28814	0.00104499	0.01050066	0.0078968	Foxp2/Foxp4/Fndc3b/Fbxw7/Abca3/Zfpm2/Wnt2/Atxn1/Meg3/Man1a2/Smad2/Hmgb1/Fgfr2/Ext1	14
GO:0043242	negative regulation of protein-	8/652	91/28814	0.00107663	0.01075358	0.008087	Nav3/Atxn7/Mphosph8/Camsap2/Hdgfl3/Sptan1/Map1a/Fgf13	8
GO:0045814	negative regulation of	8/652	91/28814	0.00107663	0.01075358	0.008087	Smyd5/Baz2a/Mphosph8/Fam172a/Kcnq1ot1/Atf7ip/Rif1/Meg3	8
GO:0016339	calcium-dependent cell-cell adhesion	5/652	35/28814	0.00108111	0.01075358	0.008087	Nlgn1/Cdh22/Nrxn1/Cdh2/Cdh13	5
GO:0099590	neurotransmitter receptor	5/652	35/28814	0.00108111	0.01075358	0.008087	Nrg1/Dnm3/Ap2b1/Myo6/Cacng2	5
GO:0060043	regulation of cardiac muscle	6/652	52/28814	0.00110314	0.01093586	0.0082241	Nrg1/Zfpm2/Wnt2/Ncam1/Fgfr2/Erbb4	6

GO:0048285	organelle fission	23/652	498/28814	0.001105	0.01093586	0.0082241	Hecw2/Eif4g3/Tom1l2/Dis3l2/Fancm/Pds5b/Ppp2r2b/Tmem135/Spire1/Akap8/Akap8l/Hnrnpu/Atrx/Stag1/Pparg/Plcb1/Mapt/Insr/Igf1r/Fgfr2/Smc3/Cdk11b/Camk2b	23
GO:0055117	regulation of cardiac muscle	7/652	71/28814	0.00111712	0.01102807	0.0082935	Ctnna3/Akap9/Atp1a2/Bin1/Slc8a1/Ryr2/Fgf13	7
GO:0048148	behavioral response to	4/652	21/28814	0.00114415	0.01121034	0.0084305	Kalrn/Sdk1/Crebbp/Parp1	4
GO:0048172	regulation of short-term	4/652	21/28814	0.00114415	0.01121034	0.0084305	Kmt2a/Slc4a10/Syn1/Grik2	4
GO:1903540	establishment of protein localization to	4/652	21/28814	0.00114415	0.01121034	0.0084305	Grip1/Snap47/Gripap1/Mapk10	4
GO:0030323	respiratory tube development	14/652	239/28814	0.00117885	0.01152166	0.0086647	Foxp2/Foxp4/Fndc3b/Fbxw7/Abca3/Zfpm2/Wnt2/Atxn1/Meg3/Man1a2/Smad2/Hmgb1/Fgfr2/Ext1	14
GO:0006816	calcium ion transport	21/652	440/28814	0.00119447	0.01164529	0.0087576	Nalcn/Chd7/Atp1a2/Micu3/Slc24a2/Bin1/Stim1/Stac/Slc8a1/Ryr2/Ryr1/Itpr1/Grin2a/Gnao1/Fgf14/Camk2b/Cacng2/Cacnb4/Cacnb2/Cacna1g/Cacna1d	21
GO:0031032	actomyosin structure organization	13/652	213/28814	0.0012056	0.01172471	0.0088174	Myo18a/Ppp1r9a/Frmd5/Cdc42bpa/Fhod3/Phactr1/Ccdc88a/Limch1/Nebl/Arap1/Tmeff2/Dlc1/Rock1	13
GO:0002053	positive regulation of mesenchymal	5/652	36/28814	0.00123237	0.01187509	0.0089305	Foxp2/Wnt2/Hmgb1/Fgfr2/Arhgap5	5
GO:0048009	insulin-like growth factor	5/652	36/28814	0.00123237	0.01187509	0.0089305	Atxn7/Gigyf2/Atxn1/Plcb1/Igf1r	5
GO:0070897	transcription preinitiation	5/652	36/28814	0.00123237	0.01187509	0.0089305	Bdp1/Taf1/Taf3/Atf7ip/Hmgb1	5
GO:0120034	positive regulation of plasma membrane	9/652	115/28814	0.00123315	0.01187509	0.0089305	Gpm6a/Bcas3/Nlgn1/Ccdc88a/Dnm3/Arap1/Dzip1/Espn/Tenm1	9
GO:0070828	heterochromatin organization	8/652	93/28814	0.00124048	0.01191649	0.0089616	Baz2a/Mphosph8/Fam172a/Kcnq1ot1/Atf7ip/Rif1/Meg3/Hp1bp3	8

GO:0006937	regulation of muscle	11/652	164/28814	0.00132825	0.01270978	0.0095582	Ctnna3/Akap9/Atp1a2/Bin1/Slc8a1/Ryr2/Ryr1/Prkg1/Prkca/Kcnma1/Fgf13	11
GO:0032273	positive regulation of	8/652	94/28814	0.00132953	0.01270978	0.0095582	Nav3/Akap9/Pde4dip/Clip1/Bin1/Tenm1/Mapt/Hsp90aa1	8
GO:0000819	sister chromatid	12/652	190/28814	0.00137298	0.01300416	0.0097796	Hecw2/Dis3l2/Pds5b/Sfpq/Hdac8/Akap8/Akap8l/Hnrnpu/Fbxw7/Atrx/Stag1/Smc3	12
GO:0018394	peptidyl-lysine acetylation	12/652	190/28814	0.00137298	0.01300416	0.0097796	Taf1/Atxn7/Phf20l1/Kmt2a/Kansl1/Setd5/Hdac8/Brd4/Kat6b/Kat2b/Ncoa1/Crebbp	12
GO:0072578	neurotransmitter-gated ion	4/652	22/28814	0.00137356	0.01300416	0.0097796	Gphn/Nlgn1/Snx27/Nrxn1	4
GO:0098877	neurotransmitter receptor	4/652	22/28814	0.00137356	0.01300416	0.0097796	Grip1/Snap47/Gripap1/Mapk10	4
GO:0046777	protein autophosphorylation	14/652	243/28814	0.00137944	0.0130284	0.0097978	Taok3/Nrg1/Wnk2/Brd4/Stk39/Map3k3/Prkca/Nlk/Insr/Igf1r/Fgfr2/Erbb4/Camk2b/Alk	14
GO:0006903	vesicle	5/652	37/28814	0.00139871	0.0130846	0.00984	Nlgn1/Gbf1/Ahi1/Pclo/Exoc4	5
GO:0035640	exploration	5/652	37/28814	0.00139871	0.0130846	0.00984	Lsamp/Kmt2a/Atp1a2/Slc4a10/Brinp1	5
GO:0036475	neuron death in response to	5/652	37/28814	0.00139871	0.0130846	0.00984	Ncoa7/Arl6ip5/Fbxw7/Tlr4/Parp1	5
GO:0051647	nucleus	5/652	37/28814	0.00139871	0.0130846	0.00984	Tacc1/Cdc42bpa/Clmn/Bin1/Kif1a	5
GO:1901879	regulation of protein	8/652	95/28814	0.00142359	0.01327997	0.009987	Nav3/Atxn7/Mphosph8/Camsap2/Hdgfl3/Sptan1/Map1a/Fgf13	8
GO:0099518	vesicle cytoskeletal	7/652	74/28814	0.00142636	0.01327997	0.009987	Ppfia2/Tanc2/Rasgrp1/Myo6/Kif5a/Kif1a/Ap3b2	7
GO:0060249	anatomical structure homeostasis	17/652	328/28814	0.00143095	0.01329127	0.0099955	Gigyf2/Kmt2a/Acaca/Ankrd11/Nell2/Stk39/Abca3/Esrrb/Ush2a/Tlr4/Prkca/Map1a/Ext1/Erbb4/Col2a1/Calb1/Pcdh15	17
GO:0098739	import across plasma membrane	12/652	191/28814	0.00143566	0.01330357	0.0100047	Atp1a3/Lrrc8c/Atp1a2/Slc24a2/Wnk2/Arl6ip5/Slc12a5/Slc6a6/Slc8a1/Slc1a6/Hcn2/Cacna1d	12
GO:0071695	anatomical structure maturation	16/652	300/28814	0.00145788	0.01347768	0.0101357	Nfasc/Slc26a8/Ankrd17/Sptbn4/Srrm4/Rere/Sclt1/Ryr1/Pparg/Ppard/Plcb1/Nrxn1/Kcnma1/Gnaq/Ext1/Ank3	16
GO:0048675	axon extension	10/652	142/28814	0.00150902	0.01391774	0.0104666	Plxna4/Nrg1/Golga4/Sema3a/Ntrk3/Mapt/Hsp90aa1/Hsp90ab1/Dscam/Dpysl2	10

GO:0031112	positive regulation of microtubule	5/652	38/28814	0.00158102	0.01451364	0.0109147	Nav3/Akap9/Pde4dip/Clip1/Mapt	5
GO:0060292	long-term	5/652	38/28814	0.00158102	0.01451364	0.0109147	Dgki/Ppp1r9a/Grid2ip/Slc24a2/Mapt	5
GO:0055067	monovalent inorganic cation	11/652	168/28814	0.00161089	0.01475334	0.011095	Atp6ap1l/Atp1a3/Atp1a2/Slc4a10/Slc12a5/Slc4a4/Slc8a1/Slc4a3/Sgk1/Kcnma1/Ext1	11
GO:0031644	regulation of nervous system	12/652	194/28814	0.00163804	0.01496715	0.0112558	Shank1/Nrg1/Nlgn1/Rims2/Akap9/Pard3/Pclo/Tlr4/Nrxn1/Ncam1/Afdn/Grin2a	12
GO:0001667	ameboidal-type cell migration	21/652	452/28814	0.00165886	0.01512222	0.0113724	Fat2/Bcas3/Kank1/Fbxw7/Map4k4/Map3k3/Slc8a1/Sema3a/Rock1/Robo1/Pparg/Ppard/Prkca/Pik3c2a/Pak3/Kitl/Hmgb1/Srgap2/Erbb4/Cdh2/Cdh13	21
GO:0071248	cellular response to	11/652	169/28814	0.00168873	0.01535887	0.0115504	Fus/Nlgn1/Cpne8/Spag16/Syt2/Syt1/Pparg/Nrxn1/Grin2a/Daxx/Ank3	11
GO:1904063	negative regulation of	8/652	98/28814	0.0017378	0.01576858	0.0118585	Hecw2/Atp1a2/Hecw1/Snx27/Stk39/Bin1/Pik3c2a/Ank3	8
GO:0009266	response to temperature	11/652	170/28814	0.00176962	0.01602037	0.0120478	Psip1/Tmem135/Slc12a5/Tlr4/Stac/Pparg/Mapt/Hsp90aa1/Hsp90ab1/Grik2/Daxx	11
GO:0071320	cellular	5/652	39/28814	0.0017802	0.0160752	0.0120891	Akap7/Akap9/Slc8a1/Itpr1/Hcn2	5
GO:0019722	calcium-mediated	12/652	196/28814	0.00178558	0.0160752	0.0120891	Ppp1r9a/Nrg1/Camta1/Tenm2/Slc8a1/Exoc4/Ryr2/Ncam1/Mapt/Itpr1/Cdh13/Cacna1d	12
GO:0010463	mesenchymal cell	6/652	57/28814	0.00178795	0.0160752	0.0120891	Foxp2/Wnt2/Zeb1/Hmgb1/Fgfr2/Arhgap5	6
GO:1901992	positive regulation of mitotic cell	8/652	99/28814	0.00185389	0.01663002	0.0125063	Ube2e2/Mta3/Ankrd17/Kmt2e/Anp32b/Brd4/Plcb1/Crebbp	8
GO:0010769	regulation of cell morphogenesis	9/652	122/28814	0.00186183	0.01666318	0.0125313	Kalrn/Il1rapl1/Kank1/Ttc3/Sgk1/Pak3/Afdn/Lrp8/Camk2b	9
GO:0008090	retrograde	4/652	24/28814	0.00192512	0.01715142	0.0128984	Map1a/Kif5a/Kif1a/Dst	4
GO:2000010	positive regulation of protein	4/652	24/28814	0.00192512	0.01715142	0.0128984	Nrg1/Map1a/Hsp90ab1/Erbb4	4
GO:0006334	nucleosome assembly	7/652	78/28814	0.00193812	0.01718935	0.012927	Anp32b/Smarca2/Kat6b/Sart3/Atrx/Hp1bp3/Daxx	7

GO:0032413	negative regulation of ion	7/652	78/28814	0.00193812	0.01718935	0.012927	Hecw2/Atp1a2/Hecw1/Snx27/Stk39/Prkca/Ank3	7
GO:1902808	positive regulation of	6/652	58/28814	0.00195674	0.01731539	0.0130217	Ube2e2/Ankrd17/Kmt2e/Anp32b/Plcb1/Crebbp	6
GO:0006476	protein deacetylation	9/652	123/28814	0.00196946	0.01738875	0.0130769	Chd3/Mta3/Baz2a/Chd4/Sfpq/Hdac8/Akap8/Akap8l/Mapt	9
GO:0016575	histone deacetylation	8/652	100/28814	0.00197602	0.01740762	0.0130911	Chd3/Mta3/Baz2a/Chd4/Sfpq/Hdac8/Akap8/Akap8l	8
GO:0032232	negative regulation of	5/652	40/28814	0.00199716	0.01751523	0.013172	Shank1/Ppp1r9a/Arap1/Tmeff2/Dlc1	5
GO:0050850	positive regulation of	5/652	40/28814	0.00199716	0.01751523	0.013172	Nrg1/Camta1/Exoc4/Ncam1/Cdh13	5
GO:0018105	peptidyl-serine phosphorylation	17/652	339/28814	0.00202638	0.01769973	0.0133108	Akap9/Sptbn4/Stk39/Tcl1b2/Tenm1/Srpk2/Sgk1/Rock1/Prkca/Ntrk3/Nsd1/Nrxn1/Nlk/Hsp90aa1/Hsp90ab1/Hipk2/Camk2b	17
GO:0061351	neural precursor cell	12/652	199/28814	0.0020272	0.01769973	0.0133108	Tacc1/Zzef1/Rere/Wnt2/Sox5/Afdn/Lhx1/Kif3a/Kif1a/Fgfr2/Fgf13/Cdh2	12
GO:0071277	cellular response to	7/652	79/28814	0.00208586	0.01817153	0.0136656	Fus/Nlgn1/Cpne8/Spag16/Syt2/Syt1/Nrxn1	7
GO:0050772	positive regulation of	8/652	101/28814	0.0021044	0.01829246	0.0137565	Robo2/Plxna4/Nrg1/Golga4/Robo1/Ntrk3/Mapt/Dscam	8
GO:1903169	regulation of calcium ion	11/652	174/28814	0.0021254	0.01843417	0.0138631	Chd7/Atp1a2/Bin1/Stim1/Stac/Slc8a1/Ryr2/Fgf14/Cacnb4/Cacnb2/Cacna1d	11
GO:0043388	positive regulation of	6/652	59/28814	0.0021373	0.01849663	0.0139101	Brd4/Tlr4/Pparg/Hmgb1/Hipk2/Parp1	6
GO:0051235	maintenance of location	17/652	342/28814	0.0022208	0.0190214	0.0143047	Chd7/Taf3/Ccdc88a/Akap9/Tmem135/Dzip1/Hnrnpu/Mprip/Slc8a1/Ryr2/Ryr1/Pparg/Ppard/Itpr1/B4galnt1/Ank3/Alb	17
GO:1901381	positive regulation of potassium ion	5/652	41/28814	0.00223279	0.0190214	0.0143047	Akap7/Kcnc2/Akap9/Wnk2/Rgs7	5
GO:1901653	cellular response to peptide	16/652	313/28814	0.00223833	0.0190214	0.0143047	Atp1a3/Tbc1d4/Kank1/Rab31/Nucks1/Tlr4/Sgk1/Rock1/Ptpre/Pparg/Plcb1/Prkca/Kat2b/Insr/Igf1r/Parp1	16

GO:0006892	post-Golgi vesicle-	8/652	102/28814	0.00223925	0.0190214	0.0143047	Exoc6/Gbf1/Rab31/Golga4/Rabep1/Exoc4/Plcb3/Ank3	8
GO:0006359	regulation of transcription	4/652	25/28814	0.0022512	0.0190214	0.0143047	Dek/Chd8/Ddx21/Tenm1	4
GO:0007635	chemosensory	4/652	25/28814	0.0022512	0.0190214	0.0143047	Kalrn/Mrgprx1/Chd7/Shank1	4
GO:0014808	release of sequestered calcium ion	4/652	25/28814	0.0022512	0.0190214	0.0143047	Chd7/Slc8a1/Ryr2/Ryr1	4
GO:0021692	cerebellar Purkinje cell	4/652	25/28814	0.0022512	0.0190214	0.0143047	Herc1/Foxp2/Sptbn2/Lhx1	4
GO:0097062	dendritic spine	4/652	25/28814	0.0022512	0.0190214	0.0143047	Zfp804a/Tanc1/Insr/Igf1r	4
GO:1902683	regulation of receptor	4/652	25/28814	0.0022512	0.0190214	0.0143047	Kalrn/Gripap1/Gpc6/Nptn	4
GO:2000311	regulation of AMPA receptor	4/652	25/28814	0.0022512	0.0190214	0.0143047	Shank1/Nlgn1/Nrxn1/Cacng2	4
GO:0006936	muscle contraction	16/652	314/28814	0.00231057	0.0194791	0.0146489	Atp1a3/Ctnna3/Akap9/Atp1a2/Bin1/Stac/Slc8a1/Ryr2/Ryr1/Prkg1/Prkca/Kcnma1/Fgf13/Cacnb2/Cacna1g/Cacna1d	16
GO:0048259	regulation of receptor-	9/652	126/28814	0.0023224	0.0194791	0.0146489	Nrg1/Pard3/Sgip1/Dnajc6/Ahi1/Prkca/Kif3a/Insr/Bicd1	9
GO:0048278	vesicle docking	6/652	60/28814	0.00233016	0.0194791	0.0146489	Rims2/Exoc6/Syt1/Exoc4/Nrxn1/Ncam1	6
GO:0050879	multicellular organismal	6/652	60/28814	0.00233016	0.0194791	0.0146489	Gigyf2/Stac/Map1a/Itpr1/Hipk2/Parp1	6
GO:0050881	musculoskeletal	6/652	60/28814	0.00233016	0.0194791	0.0146489	Gigyf2/Stac/Map1a/Itpr1/Hipk2/Parp1	6
GO:0001894	tissue homeostasis	15/652	286/28814	0.00237488	0.01977606	0.0148722	Gigyf2/Kmt2a/Acaca/Ankrd11/Stk39/Abca3/Esrrb/Ush2a/Tlr4/Prkca/Map1a/Ext1/Erbb4/Col2a1/Pcdh15	15
GO:0014066	regulation of phosphatidylinositol 3-kinase	8/652	103/28814	0.00238078	0.01977606	0.0148722	Nrg1/Rasgrp1/Ptpn13/Ppard/Ntrk3/Insr/Igf1r/Erbb4	8
GO:0021761	limbic system development	8/652	103/28814	0.00238078	0.01977606	0.0148722	Atp1a2/Sema3a/Lrp8/Kif3a/Fgfr2/Fgf13/Epha5/Alk	8
GO:0022406	membrane docking	7/652	81/28814	0.00240733	0.01995446	0.0150064	Rims2/Exoc6/Syt1/Exoc4/Rock1/Nrxn1/Ncam1	7
GO:0006904	vesicle docking involved in	5/652	42/28814	0.00248804	0.02049369	0.0154119	Rims2/Exoc6/Exoc4/Nrxn1/Ncam1	5

GO:0010092	specification of animal organ	5/652	42/28814	0.00248804	0.02049369	0.0154119	Robo2/Wnt2/Robo1/Fgfr2/Ext1	5
GO:1902275	regulation of chromatin	5/652	42/28814	0.00248804	0.02049369	0.0154119	Mphosph8/Setd5/Atf7ip/Hnrnpu/Ssrp1	5
GO:0046660	female sex differentiation	10/652	152/28814	0.00249485	0.02050686	0.0154218	Nefh/Chd7/Mir124a-1hg/Csmd1/Dach2/Zfpm2/Kitl/Lhx1/Insr/Dach1	10
GO:0042596	fear response	6/652	61/28814	0.00253581	0.0208	0.0156423	Kalrn/Atp1a2/Spire1/Brinp1/Grik2/Ext1	6
GO:0042692	muscle cell differentiation	20/652	438/28814	0.00257895	0.02110976	0.0158752	Sorbs2/Fhod3/Nrg1/Ankrd17/Neb1/Spag9/Smarca2/Tanc1/Hnrnpu/Bin1/Zeb1/Supt6/Stim1/Sox6/Slc8a1/Ryr1/Fgfr2/Daxx/Cdh2/Cacnb4	20
GO:0042752	regulation of circadian	9/652	128/28814	0.00258437	0.02111017	0.0158755	Nlgn1/Sfpq/Fbxl17/Fbxw7/Mapk10/Pparg/Zfhx3/Arntl/Alb	9
GO:0006883	cellular sodium ion homeostasis	4/652	26/28814	0.0026134	0.02111048	0.0158758	Atp1a3/Atp1a2/Slc8a1/Sgk1	4
GO:0016082	synaptic	4/652	26/28814	0.0026134	0.02111048	0.0158758	Cadps2/Rims2/Erc1/Snap47	4
GO:0050927	positive regulation of	4/652	26/28814	0.0026134	0.02111048	0.0158758	Prkca/Ntrk3/Hmgb1/Cdh13	4
GO:0071108	protein K48-linked	4/652	26/28814	0.0026134	0.02111048	0.0158758	Otud7a/Usp8/Usp29/Usp34	4
GO:1903514	release of sequestered calcium ion	4/652	26/28814	0.0026134	0.02111048	0.0158758	Chd7/Slc8a1/Ryr2/Ryr1	4
GO:0030010	establishment of cell	10/652	153/28814	0.00261665	0.02111048	0.0158758	Myo18a/Bcas3/Kank1/Gbf1/Pard3/Exoc4/Igf1r/Hsp90aa1/Hsp90ab1/Fgf13	10
GO:0044843	cell cycle G1/S phase transition	13/652	233/28814	0.0026642	0.0214062	0.0160982	Gigyf2/Ube2e2/Ankrd17/Senp2/Kmt2e/Anp32b/Usp29/Brd4/Esrrb/Plcb1/Crebbp/Camk2b/Cacnb4	13
GO:0051056	regulation of small GTPase mediated signal	13/652	233/28814	0.0026642	0.0214062	0.0160982	Dgki/Dennd1a/Cdc42bpa/Nrg1/Kank1/Dennd4a/Arhgap26/Dlc1/Map4k4/Robo1/Rasgrp1/Kitl/Cdh2	13
GO:0014706	striated muscle tissue development	15/652	290/28814	0.0027093	0.02172418	0.0163373	Chd7/Sorbs2/Fhod3/Nrg1/Nebl/Luc7l/Hnrnpu/Zfpm2/Wnt2/Sox6/Slc8a1/Ryr2/Ncam1/Fgfr2/Erbb4	15
GO:0021575	hindbrain	6/652	62/28814	0.0027548	0.02199917	0.0165441	Herc1/Foxp2/Dlc1/Sptbn2/Nrxn1/Lhx1	6

GO:0046622	positive regulation of	6/652	62/28814	0.0027548	0.02199917	0.0165441	Nrg1/Zfpm2/Wnt2/Ncam1/Fgfr2/Erbb4	6
GO:0042220	response to	5/652	43/28814	0.0027638	0.02202631	0.0165645	Kalrn/Sdk1/Ncam1/Crebbp/Parp1	5
GO:0071902	positive regulation of protein	12/652	207/28814	0.00280493	0.02230886	0.016777	Taok3/Fgd4/Tcl1b2/Tenm1/Tlr4/Robo1/Rasgrp1/Ntrk3/Nrxn1/Kitl/Insr/Hsp90ab1	12
GO:0043406	positive regulation of	9/652	130/28814	0.00286922	0.02277405	0.0171268	Taok3/Fgd4/Tenm1/Tlr4/Robo1/Rasgrp1/Ntrk3/Kitl/Insr	9
GO:0031507	heterochromatin assembly	7/652	84/28814	0.00295946	0.02335419	0.0175631	Baz2a/Mphosph8/Fam172a/Kcnq1ot1/Atf7ip/Rif1/Meg3	7
GO:0050848	regulation of calcium-	7/652	84/28814	0.00295946	0.02335419	0.0175631	Nrg1/Camta1/Exoc4/Ncam1/Mapt/Itpr1/Cdh13	7
GO:0006893	Golgi to plasma membrane	6/652	63/28814	0.00298764	0.02335419	0.0175631	Exoc6/Rab31/Golga4/Rabep1/Exoc4/Ank3	6
GO:0044091	membrane	6/652	63/28814	0.00298764	0.02335419	0.0175631	Il1rapl1/Nlgn1/Exoc4/Nrxn1/Cdh2/Ank3	6
GO:0048260	positive regulation of	6/652	63/28814	0.00298764	0.02335419	0.0175631	Pard3/Sgip1/Ahi1/Kif3a/Insr/Bicd1	6
GO:0021884	forebrain	4/652	27/28814	0.00301364	0.02335419	0.0175631	Slc4a10/Sema3a/Gnaq/Fgfr2	4
GO:0031338	regulation of	4/652	27/28814	0.00301364	0.02335419	0.0175631	Tbc1d4/Trim9/Syt2/Syt1	4
GO:0050926	regulation of positive	4/652	27/28814	0.00301364	0.02335419	0.0175631	Prkca/Ntrk3/Hmgb1/Cdh13	4
GO:0051571	positive regulation of	4/652	27/28814	0.00301364	0.02335419	0.0175631	Kmt2a/Kansl1/Rtf1/Kmt2e	4
GO:0060004	reflex	4/652	27/28814	0.00301364	0.02335419	0.0175631	Shank1/Foxp2/Kcnma1/Pcdh15	4
GO:0060008	Sertoli cell	4/652	27/28814	0.00301364	0.02335419	0.0175631	Safb2/Akap9/Arid4b/Atrx	4
GO:0086019	cell-cell signaling	4/652	27/28814	0.00301364	0.02335419	0.0175631	Ryr2/Cacnb2/Cacna1g/Cacna1d	4
GO:0007616	long-term	5/652	44/28814	0.00306101	0.02367464	0.0178041	Shank1/Chd8/Sgk1/Crebbp/Calb1	5
GO:0071241	cellular response to	13/652	239/28814	0.00331175	0.0255636	0.0192247	Kcnc2/Fus/Nlgn1/Cpne8/Spag16/Atrx/Syt2/Syt1/Pparg/Nrxn1/Grin2a/Daxx/Ank3	13
GO:0010921	regulation of phosphatase	7/652	86/28814	0.00337809	0.02594208	0.0195093	Ppp4r4/Ppp2r2b/Mprip/Hsp90b1/Rock1/Ppp1r12a/Hsp90ab1	7
GO:0060415	muscle tissue morphogenesis	7/652	86/28814	0.00337809	0.02594208	0.0195093	Chd7/Nrg1/Adarb1/Zfpm2/Wnt2/Ryr2/Fgfr2	7
GO:0072583	clathrin-dependent	5/652	45/28814	0.00338059	0.02594208	0.0195093	Sgip1/Dnajc6/Ap2b1/Itsn2/Canx	5

GO:1903305	regulation of regulated	11/652	185/28814	0.00341137	0.02609584	0.0196249	Ppfia2/Dgki/Nlgn1/Rims2/Trim9/Syt2/Syt1/Prkca/Myo6/Cacnb4/Cacna1g	11
GO:0098657	import into cell	13/652	240/28814	0.00343117	0.02609584	0.0196249	Atp1a3/Plppr4/Lrrc8c/Atp1a2/Slc24a2/Wnk2/Arl6ip5/Slc12a5/Slc6a6/Slc8a1/Slc1a6/Hcn2/Cacna1d	13
GO:0032148	activation of protein kinase	4/652	28/28814	0.00345376	0.02609584	0.0196249	Nrg1/Ccdc88a/Ntrk3/Insr	4
GO:0038083	peptidyl-tyrosine	4/652	28/28814	0.00345376	0.02609584	0.0196249	Nrg1/Insr/Igf1r/Erbb4	4
GO:0045822	negative regulation of	4/652	28/28814	0.00345376	0.02609584	0.0196249	Atp1a2/Sptbn4/Bin1/Prkca	4
GO:0098884	postsynaptic neurotransmitte	4/652	28/28814	0.00345376	0.02609584	0.0196249	Nrg1/Dnm3/Ap2b1/Myo6	4
GO:0140239	postsynaptic	4/652	28/28814	0.00345376	0.02609584	0.0196249	Nrg1/Dnm3/Ap2b1/Myo6	4
GO:1901380	negative regulation of potassium ion	4/652	28/28814	0.00345376	0.02609584	0.0196249	Snx27/Stk39/Bin1/Ank3	4
GO:0060038	cardiac muscle cell	6/652	65/28814	0.00349703	0.02627664	0.0197609	Nrg1/Zfpm2/Wnt2/Ncam1/Fgfr2/Erbb4	6
GO:0098930	axonal	6/652	65/28814	0.00349703	0.02627664	0.0197609	Map1a/Kif5a/Kif3a/Kif1a/Dst/Ap3b2	6
GO:0071375	cellular response to peptide hormone	14/652	269/28814	0.00349776	0.02627664	0.0197609	Tbc1d4/Kank1/Rab31/Nucks1/Sgk1/Rock1/Ptpre/Pparg/Plcb1/Prkca/Kat2b/Insr/Igf1r/Parp1	14
GO:0014074	response to purine-	8/652	110/28814	0.0035781	0.02677778	0.0201378	Akap7/Akap9/Hsp90b1/Slc8a1/Ryr2/Ryr1/Itpr1/Hcn2	8
GO:0034446	substrate adhesion-	8/652	110/28814	0.0035781	0.02677778	0.0201378	Atrnl1/Kank1/Ntng1/Fndc3b/Tmeff2/Ush2a/Srgap2/Atrn	8
GO:0048738	cardiac muscle tissue development	14/652	270/28814	0.00361473	0.02700051	0.0203053	Chd7/Sorbs2/Fhod3/Nrg1/Nebl/Hnrnpu/Zfpm2/Wnt2/Sox6/Slc8a1/Ryr2/Ncam1/Fgfr2/Erbb4	14
GO:0035601	protein deacylation	9/652	135/28814	0.00369046	0.02746176	0.0206521	Chd3/Mta3/Baz2a/Chd4/Sfpq/Hdac8/Akap8/Akap8l/Mapt	9
GO:0098732	macromolecule deacylation	9/652	135/28814	0.00369046	0.02746176	0.0206521	Chd3/Mta3/Baz2a/Chd4/Sfpq/Hdac8/Akap8/Akap8l/Mapt	9
GO:0006383	transcription by RNA	5/652	46/28814	0.00372346	0.0276028	0.0207582	Bdp1/Dek/Chd8/Ddx21/Tenm1	5

GO:0008631	intrinsic apoptotic signaling	5/652	46/28814	0.00372346	0.0276028	0.0207582	Sfpq/Arl6ip5/Fbxw7/Mapt/Parp1	5
GO:0016482	cytosolic transport	10/652	161/28814	0.00377172	0.02787707	0.0209645	Ppfia2/Heatr5a/Erc1/Gbf1/Tanc2/Spag9/Washc2/Kif5a/Kif1a/Atp9a	10
GO:0098900	regulation of action	6/652	66/28814	0.00377465	0.02787707	0.0209645	Ctnna3/Akap9/Bin1/Ryr2/Fgf13/Ank3	6
GO:0006942	regulation of striated muscle	7/652	88/28814	0.00384038	0.02830932	0.0212895	Ctnna3/Akap9/Atp1a2/Bin1/Slc8a1/Ryr2/Fgf13	7
GO:0014065	phosphatidylinositol 3-kinase	9/652	136/28814	0.00387483	0.02850974	0.0214403	Nrg1/Rasgrp1/Ptpn13/Ppard/Pik3c2a/Ntrk3/Insr/Igf1r/Erbb4	9
GO:0003148	outflow tract septum	4/652	29/28814	0.00393561	0.02875178	0.0216223	Robo2/Zfpm2/Robo1/Fgfr2	4
GO:0030318	melanocyte	4/652	29/28814	0.00393561	0.02875178	0.0216223	Lrmda/Mitf/Kitl/Gnaq	4
GO:1904861	excitatory	4/652	29/28814	0.00393561	0.02875178	0.0216223	Nlgn1/Nptn/Ntrk3/Nrxn1	4
GO:0022618	ribonucleoprotein complex	10/652	162/28814	0.00394071	0.02875178	0.0216223	Setx/Sfswap/Luc7l2/Psip1/Luc7l/Sart3/Srpk2/Hsp90aa1/Hsp90ab1/Eif3a	10
GO:0045137	development of primary sexual characteristics	13/652	244/28814	0.00394431	0.02875178	0.0216223	Nefh/Safb2/Jmjd1c/Akap9/Arid4b/Csmd1/Dach2/Zfpm2/Atrx/Sema3a/Kitl/Insr/Dach1	13
GO:0006275	regulation of DNA replication	9/652	137/28814	0.00406633	0.02943541	0.0221364	Zbtb38/Ccdc88a/Nucks1/Ankrd17/Senp2/Atrx/Rbbp6/Dach1/Smc3	9
GO:0031623	receptor internalization	9/652	137/28814	0.00406633	0.02943541	0.0221364	Nrg1/Rab31/Dnm3/Pard3/Ap2b1/Ahi1/Myo6/Insr/Cacng2	9
GO:0050805	negative regulation of	7/652	89/28814	0.00408879	0.02943541	0.0221364	Dgki/Ppp1r9a/Grid2ip/Slc24a2/Gabbr1/Mapt/Grik2	7
GO:1901019	regulation of calcium ion transmembrane	7/652	89/28814	0.00408879	0.02943541	0.0221364	Atp1a2/Stim1/Stac/Ryr2/Fgf14/Cacnb4/Cacnb2	7
GO:1904427	positive regulation of calcium ion	7/652	89/28814	0.00408879	0.02943541	0.0221364	Stim1/Stac/Ryr2/Fgf14/Cacnb4/Cacnb2/Cacna1d	7
GO:0000266	mitochondrial	5/652	47/28814	0.00409054	0.02943541	0.0221364	Ppp2r2b/Tmem135/Spire1/Pparg/Mapt	5
GO:0021696	cerebellar cortex	5/652	47/28814	0.00409054	0.02943541	0.0221364	Herc1/Foxp2/Sptbn2/Nrxn1/Lhx1	5

GO:0010563	negative regulation of phosphorus metabolic	20/652	457/28814	0.00414419	0.02971262	0.0223449	Atxn7/Adarb1/Pard3/Ppp4r4/Stk39/Mfhas1/Hnrnpu/Mprip/Slc8a1/Atxn1/Rock1/Ptpn13/Pparg/Prkca/Kat2b/Ntrk3/Mapt/Insr/Gnaq/Parp1	20
GO:0045936	negative regulation of phosphate metabolic	20/652	457/28814	0.00414419	0.02971262	0.0223449	Atxn7/Adarb1/Pard3/Ppp4r4/Stk39/Mfhas1/Hnrnpu/Mprip/Slc8a1/Atxn1/Rock1/Ptpn13/Pparg/Prkca/Kat2b/Ntrk3/Mapt/Insr/Gnaq/Parp1	20
GO:0030516	regulation of axon extension	8/652	113/28814	0.00421501	0.03015747	0.0226794	Plxna4/Nrg1/Golga4/Sema3a/Ntrk3/Mapt/Dscam/Dpysl2	8
GO:0032868	response to insulin	13/652	246/28814	0.00422322	0.03015747	0.0226794	Tbc1d4/Kank1/Rab31/Nucks1/Sgk1/Ptpre/Pparg/Plcb1/Prkca/Kat2b/Insr/Igf1r/Parp1	13
GO:0021859	pyramidal neuron	3/652	15/28814	0.00428299	0.03015747	0.0226794	Unc5d/Slc4a10/Fgfr2	3
GO:0021932	hindbrain radial glia	3/652	15/28814	0.00428299	0.03015747	0.0226794	Rbfox2/Rere/Ctnna2	3
GO:0030007	cellular potassium ion	3/652	15/28814	0.00428299	0.03015747	0.0226794	Atp1a3/Atp1a2/Kcnma1	3
GO:0031000	response to	3/652	15/28814	0.00428299	0.03015747	0.0226794	Slc8a1/Ryr2/Ryr1	3
GO:0048680	positive regulation of	3/652	15/28814	0.00428299	0.03015747	0.0226794	Nrg1/Ntrk3/Igf1r	3
GO:0061577	calcium ion transmembrane transport via	3/652	15/28814	0.00428299	0.03015747	0.0226794	Bin1/Cacnb2/Cacna1d	3
GO:0071599	otic vesicle	3/652	15/28814	0.00428299	0.03015747	0.0226794	Ahi1/Fgfr2/Col2a1	3
GO:0098917	retrograde trans-synaptic	3/652	15/28814	0.00428299	0.03015747	0.0226794	Nlgn1/Tenm2/Plcb1	3
GO:0006835	dicarboxylic acid transport	7/652	90/28814	0.00434916	0.03056861	0.0229886	Slc26a8/Lrrc8c/Arl6ip5/Gabbr1/Slc1a6/Myo6/Dpysl2	7
GO:0022029	telencephalon	6/652	68/28814	0.00437846	0.03066481	0.0230609	Nrg1/Robo1/Nrg3/Lrp8/Srgap2/Fgf13	6
GO:0044458	motile cilium	6/652	68/28814	0.00437846	0.03066481	0.0230609	Ttll5/Armc2/Spag16/Dzip1/Ahi1/Kif3a	6
GO:0007214	gamma-aminobutyric	4/652	30/28814	0.00446096	0.0310214	0.0233291	Gabbr1/Gabrb2/Gabra6/Cacnb4	4
GO:0018200	peptidyl-glutamic acid	4/652	30/28814	0.00446096	0.0310214	0.0233291	Ttll5/Agbl1/Ttll7/Parp1	4

GO:0045932	negative regulation of	4/652	30/28814	0.00446096	0.0310214	0.0233291	Atp1a2/Bin1/Prkg1/Kcnma1	4
GO:1903523	negative regulation of	4/652	30/28814	0.00446096	0.0310214	0.0233291	Atp1a2/Sptbn4/Bin1/Prkca	4
GO:0001558	regulation of cell growth	20/652	461/28814	0.00455831	0.03164237	0.0237961	Fstl4/Plxna4/Clstn3/Nrg1/Rims2/Smarca2/Golga4/Syt2/Syt1/Itsn2/Sgk1/Sema3a/Pparg/Ppard/Ntrk3/Mapt/Afdn/Fgf13/Dscam/Dpysl2	20
GO:0046683	response to organophosphoru	7/652	91/28814	0.00462187	0.03202705	0.0240854	Akap7/Akap9/Hsp90b1/Slc8a1/Ryr1/Itpr1/Hcn2	7
GO:0006470	protein dephosphorylation	13/652	249/28814	0.00467122	0.03231198	0.0242997	Camta1/Ppp4r4/Ppp2r2b/Mfhas1/Dlc1/Mprip/Hsp90b1/Rock1/Ptpre/Ptpn4/Ptpn13/Ppp1r12a/Hsp90ab1	13
GO:0099601	regulation of neurotransmitter receptor	6/652	69/28814	0.00470573	0.03249351	0.0244362	Shank1/Nlgn1/Akap9/Nrxn1/Grin2a/Cacng2	6
GO:0045761	regulation of adenylate	5/652	49/28814	0.00490098	0.03364933	0.0253054	Akap9/Cap2/Gabbr1/Stim1/Cacna1d	5
GO:1990573	potassium ion import across	5/652	49/28814	0.00490098	0.03364933	0.0253054	Atp1a3/Atp1a2/Wnk2/Slc12a5/Hcn2	5
GO:0008584	male gonad development	9/652	141/28814	0.00490737	0.03364933	0.0253054	Safb2/Jmjd1c/Akap9/Arid4b/Csmd1/Zfpm2/Atrx/Sema3a/Insr	9
GO:1904375	regulation of protein localization to	9/652	141/28814	0.00490737	0.03364933	0.0253054	Kalrn/Dpp10/Mrap2/Gripap1/Gpc6/Stac/Nrxn1/Camk2b/Cacng2	9
GO:0098659	inorganic cation import	8/652	116/28814	0.00493582	0.03372668	0.0253636	Atp1a3/Atp1a2/Slc24a2/Wnk2/Slc12a5/Slc8a1/Hcn2/Cacna1d	8
GO:0099587	inorganic ion import across	8/652	116/28814	0.00493582	0.03372668	0.0253636	Atp1a3/Atp1a2/Slc24a2/Wnk2/Slc12a5/Slc8a1/Hcn2/Cacna1d	8
GO:0002026	regulation of the force of	4/652	31/28814	0.00503153	0.03396716	0.0255444	Atp1a2/Slc8a1/Ryr2/Prkca	4
GO:0007097	nuclear	4/652	31/28814	0.00503153	0.03396716	0.0255444	Tacc1/Cdc42bpa/Clmn/Kif1a	4
GO:0021533	cell differentiation	4/652	31/28814	0.00503153	0.03396716	0.0255444	Herc1/Foxp2/Nrxn1/Lhx1	4

GO:0051123	RNA polymerase II preinitiation	4/652	31/28814	0.00503153	0.03396716	0.0255444	Taf1/Taf3/Atf7ip/Hmgb1	4
GO:0060384	innervation	4/652	31/28814	0.00503153	0.03396716	0.0255444	Chd7/Adarb1/Sema3a/Gabrb2	4
GO:0099633	protein localization to postsynaptic	4/652	31/28814	0.00503153	0.03396716	0.0255444	Kalrn/Gphn/Gpc6/Cacng2	4
GO:0099645	neurotransmitter receptor localization to	4/652	31/28814	0.00503153	0.03396716	0.0255444	Kalrn/Gphn/Gpc6/Cacng2	4
GO:0140014	mitotic nuclear division	14/652	281/28814	0.00512478	0.03446042	0.0259154	Hecw2/Tom1l2/Dis3l2/Pds5b/Akap8/Akap81/Hnrnpu/Atrx/Stag1/Insr/Igf1r/Fgfr2/Smc3/Cdk11b	14
GO:0046546	development of primary male	9/652	142/28814	0.00513734	0.03446042	0.0259154	Safb2/Jmjd1c/Akap9/Arid4b/Csmd1/Zfpm2/Atrx/Sema3a/Insr	9
GO:0046879	hormone secretion	17/652	372/28814	0.00517353	0.03446042	0.0259154	Kalrn/Chd7/Nrg1/Rims2/Selenom/Gabbr1/Nell2/Pclo/Pparg/Ppard/Pde1c/Nrxn1/Myt1/Smad2/Epha5/Cacna1d/Arntl	17
GO:0055057	neuroblast	3/652	16/28814	0.00518354	0.03446042	0.0259154	Sox5/Fgfr2/Fgf13	3
GO:0070572	positive regulation of	3/652	16/28814	0.00518354	0.03446042	0.0259154	Nrg1/Ntrk3/Igf1r	3
GO:0070593	dendrite self-	3/652	16/28814	0.00518354	0.03446042	0.0259154	Nptn/Ext1/Dscam	3
GO:0090128	regulation of synapse	3/652	16/28814	0.00518354	0.03446042	0.0259154	Nrg2/Nrxn1/Camk2b	3
GO:0098885	modification of postsynaptic	3/652	16/28814	0.00518354	0.03446042	0.0259154	Kalrn/Cttnbp2/Ctnna2	3
GO:1903423	positive regulation of	3/652	16/28814	0.00518354	0.03446042	0.0259154	Nlgn1/Dnm3/Pclo	3
GO:0042490	mechanoreceptor differentiation	7/652	93/28814	0.00520574	0.03454956	0.0259824	Ush2a/Ntrk3/Myo6/Kif3a/Kcnma1/Gabrb2/Pcdh15	7
GO:0007265	Ras protein signal transduction	16/652	342/28814	0.0052729	0.0349363	0.0262732	Kalrn/Rasgef1b/Dgki/Dennd1a/Nrg1/Kank1/Dennd4a/Usp8/Dlc1/Map4k4/Rock1/Robo1/Rasgrp1/Kitl/Cdh2/Cdh13	16

GO:0006874	cellular calcium ion homeostasis	21/652	500/28814	0.00533959	0.03531862	0.0265608	Chd7/Thada/Atp1a2/Micu3/Slc24a2/Tmem178/Stim1/Slc8a1/Nptn/Ryr2/Ryr1/Prkca/Itpr1/Hmgb1/Grin2a/Grik2/Cnga1/Calb1/Cacnb4/Cacna1g/Cacna1d	21
GO:0017156	calcium-ion regulated	6/652	71/28814	0.00541373	0.03568885	0.0268392	Cadps2/Rims2/Trim9/Syt2/Syt1/Cacna1g	6
GO:0021885	forebrain cell	6/652	71/28814	0.00541373	0.03568885	0.0268392	Nrg1/Robo1/Nrg3/Lrp8/Srgap2/Fgf13	6
GO:0043523	regulation of neuron apoptotic	14/652	283/28814	0.00544733	0.03575445	0.0268885	Trim2/Ppp2r2b/Cpeb4/Fbxw7/Srpk2/Rock1/Pak3/Kcnma1/Hsp90ab1/Hipk2/Grik2/Gabrb2/Cntfr/Parp1	14
GO:0043010	camera-type eye development	17/652	374/28814	0.00545035	0.03575445	0.0268885	Sdk1/Chd7/Ttll5/Gpm6a/Kmt2c/Foxp2/Pds5b/Ahi1/Tenm3/Zeb1/Ntrk3/Mitf/Lhx1/Hipk2/Fgfr2/Dscam/Calb1	17
GO:0051651	maintenance of location in	12/652	225/28814	0.00545098	0.03575445	0.0268885	Chd7/Taf3/Ccdc88a/Akap9/Hnrnpu/Mprip/Slc8a1/Ryr2/Ryr1/Itpr1/Ank3/Alb	12
GO:0010631	epithelial cell migration	15/652	313/28814	0.00547058	0.03582317	0.0269402	Fat2/Bcas3/Kank1/Fbxw7/Map4k4/Map3k3/Sema3a/Rock1/Robo1/Pparg/Ppard/Prkca/Pik3c2a/Hmgb1/Cdh13	15
GO:0048644	muscle organ morphogenesis	7/652	94/28814	0.00551762	0.03601587	0.0270851	Chd7/Nrg1/Adarb1/Zfpm2/Wnt2/Ryr2/Fgfr2	7
GO:0071826	ribonucleoprotein complex	10/652	170/28814	0.00551834	0.03601587	0.0270851	Setx/Sfswap/Luc7l2/Psip1/Luc7l/Sart3/Srpk2/Hsp90aa1/Hsp90ab1/Eif3a	10
GO:0071560	cellular response to transforming	12/652	226/28814	0.00564205	0.03674668	0.0276347	Zeb1/Sox6/Sox5/Glg1/Pparg/Nlk/Smad2/Igf1r/Hsp90ab1/Hipk2/Crebbp/Parp1	12
GO:0070296	sarcoplasmic reticulum	4/652	32/28814	0.00564902	0.03674668	0.0276347	Chd7/Slc8a1/Ryr2/Ryr1	4
GO:0090132	epithelium migration	15/652	315/28814	0.00579132	0.03751357	0.0282114	Fat2/Bcas3/Kank1/Fbxw7/Map4k4/Map3k3/Sema3a/Rock1/Robo1/Pparg/Ppard/Prkca/Pik3c2a/Hmgb1/Cdh13	15
GO:0046579	positive regulation of	6/652	72/28814	0.00579556	0.03751357	0.0282114	Dgki/Nrg1/Map4k4/Robo1/Rasgrp1/Kitl	6
GO:0055008	cardiac muscle tissue	6/652	72/28814	0.00579556	0.03751357	0.0282114	Chd7/Nrg1/Zfpm2/Wnt2/Ryr2/Fgfr2	6
GO:0008089	anterograde	5/652	51/28814	0.00581913	0.03757485	0.0282575	Map1a/Kif5a/Kif3a/Kif1a/Ap3b2	5

GO:0048545	response to steroid hormone	12/652	227/28814	0.0058384	0.03757485	0.0282575	Safb2/Atp1a2/Rbfox2/Hdac8/Hnrnpu/Sgk1/Ncoa1/Kcnma1/Hmgb1/Daxx/Arntl/Parp1	12
GO:0070374	positive regulation of	12/652	227/28814	0.0058384	0.03757485	0.0282575	Nrg1/Mfhas1/Fbxw7/Tlr4/Nptn/Rasgrp1/Prkca/Nrxn1/Hmgb1/Fgfr2/Erbb4/Dcc	12
GO:0048864	stem cell development	7/652	95/28814	0.00584328	0.03757485	0.0282575	Msi2/Fam172a/Tcof1/Sema3a/Kitl/Erbb4/Cdh2	7
GO:0016197	endosomal transport	12/652	228/28814	0.00604013	0.03877726	0.0291618	Heatr5a/Dennd1a/Erc1/Gbf1/Lrba/Snx27/Grip1/Spag9/Gripap1/Washc2/Itsn2/Atp9a	12
GO:0090130	tissue migration	15/652	317/28814	0.00612707	0.03895462	0.0292951	Fat2/Bcas3/Kank1/Fbxw7/Map4k4/Map3k3/Sema3a/Rock1/Robo1/Pparg/Ppard/Prkca/Pik3c2a/Hmgb1/Cdh13	15
GO:0045165	cell fate commitment	14/652	287/28814	0.00614142	0.03895462	0.0292951	Nrg1/Rtf1/Wnt2/Sox6/Sox5/Pparg/Ntrk3/Myt1l/Mitf/Smad2/Fgfr2/Fgf13/Ext1/Erbb4	14
GO:0051924	regulation of calcium ion transport	14/652	287/28814	0.00614142	0.03895462	0.0292951	Chd7/Atp1a2/Bin1/Stim1/Stac/Slc8a1/Ryr2/Itpr1/Gnao1/Fgf14/Cacnb4/Cacnb2/Cacna1g/Cacna1d	14
GO:0030833	regulation of actin filament	9/652	146/28814	0.00614178	0.03895462	0.0292951	Ppp1r9a/Fhod3/Kank1/Cyfip2/Mphosph8/Bin1/Tenm1/Sptan1/Pak3	9
GO:2000045	regulation of G1/S transition	9/652	146/28814	0.00614178	0.03895462	0.0292951	Gigyf2/Ube2e2/Ankrd17/Senp2/Kmt2e/Anp32b/Plcb1/Crebbp/Cacnb4	9
GO:0033127	regulation of histone	3/652	17/28814	0.00618954	0.03895462	0.0292951	Akap8/Akap8l/Daxx	3
GO:0042711	maternal	3/652	17/28814	0.00618954	0.03895462	0.0292951	Kalrn/Brinp1/Gnaq	3
GO:0061000	negative regulation of	3/652	17/28814	0.00618954	0.03895462	0.0292951	Fstl4/Nlgn1/Dnm3	3
GO:0098814	spontaneous synaptic	3/652	17/28814	0.00618954	0.03895462	0.0292951	Ppp1r9a/Rims2/Syt1	3
GO:1903358	regulation of Golgi	3/652	17/28814	0.00618954	0.03895462	0.0292951	Akap9/Pde4dip/Camsap2	3
GO:2000291	regulation of myoblast	3/652	17/28814	0.00618954	0.03895462	0.0292951	Malat1/Paxbp1/Ppard	3
GO:2000651	positive regulation of sodium ion	3/652	17/28814	0.00618954	0.03895462	0.0292951	Wnk2/Plcb1/Ank3	3

GO:0043954	cellular component	6/652	73/28814	0.00619665	0.03895462	0.0292951	Zfp804a/Erc1/Tanc1/Afdn/Insr/Igf1r	6
GO:0021697	cerebellar	4/652	33/28814	0.00631504	0.03948991	0.0296977	Herc1/Foxp2/Nrxn1/Lhx1	4
GO:1904646	cellular response to	4/652	33/28814	0.00631504	0.03948991	0.0296977	Atp1a3/Tlr4/Igf1r/Parp1	4
GO:0060428	lung epithelium	5/652	52/28814	0.00632084	0.03948991	0.0296977	Foxp2/Foxp4/Fndc3b/Wnt2/Fgfr2	5
GO:0046578	regulation of Ras protein	11/652	201/28814	0.006322	0.03948991	0.0296977	Dgki/Dennd1a/Nrg1/Kank1/Dennd4a/Dlc1/Map4k4/Robo1/Rasgrp1/Kitl/Cdh2	11
GO:1901989	positive regulation of	8/652	121/28814	0.00634235	0.03955411	0.029746	Ube2e2/Mta3/Ankrd17/Kmt2e/Anp32b/Brd4/Plcb1/Crebbp	8
GO:0009914	hormone transport	17/652	380/28814	0.00635406	0.03956437	0.0297537	Kalrn/Chd7/Nrg1/Rims2/Selenom/Gabbr1/Nell2/Pclo/Pparg/Ppard/Pde1c/Nrxn1/Myt1/Smad2/Epha5/Cacna1d/Arntl	17
GO:0010469	regulation of signaling	9/652	147/28814	0.00641501	0.03988065	0.0299916	Shank1/Nlgn1/Akap9/Fbxw7/Pparg/Nrxn1/Grin2a/Cacng2/Bicd1	9
GO:0000280	nuclear division	19/652	445/28814	0.00660751	0.04094549	0.0307923	Hecw2/Eif4g3/Tom1l2/Dis3l2/Fancm/Pds5b/Spire1/Akap8/Akap8l/Hnrnpu/Atrx/Stag1/Plcb1/Insr/Igf1r/Fgfr2/Smc3/Cdk11b/Camk2b	19
GO:0034394	protein localization to	6/652	74/28814	0.00661756	0.04094549	0.0307923	Nrg1/Gbf1/Astn2/Map1a/Hsp90ab1/Erbb4	6
GO:0043966	histone H3	6/652	74/28814	0.00661756	0.04094549	0.0307923	Atxn7/Kmt2a/Hdac8/Brd4/Kat6b/Kat2b	6
GO:0042177	negative regulation of	8/652	122/28814	0.00665677	0.04112336	0.0309261	Nell1/Nrg1/Mycbp2/Map1a/Hsp90ab1/Hipk2/Grin2a/Fhit	8
GO:0030072	peptide hormone secretion	14/652	290/28814	0.00670709	0.04136916	0.031111	Kalrn/Chd7/Rims2/Gabbr1/Pclo/Pparg/Ppard/Pde1c/Nrxn1/Myt1/Smad2/Epha5/Cacna1d/Arntl	14
GO:0045598	regulation of fat cell	9/652	149/28814	0.00698941	0.04296344	0.0323099	Trio/Tfe3/Fndc3b/Gps2/Hnrnpu/Zfpm2/Pparg/Ppard/Arntl	9
GO:0035308	negative regulation of	4/652	34/28814	0.00703118	0.04296344	0.0323099	Ppp4r4/Mfhas1/Mprip/Rock1	4
GO:0051497	negative regulation of	4/652	34/28814	0.00703118	0.04296344	0.0323099	Ppp1r9a/Arap1/Tmeff2/Dlc1	4
GO:0055075	potassium ion	4/652	34/28814	0.00703118	0.04296344	0.0323099	Atp1a3/Atp1a2/Slc12a5/Kcnma1	4
GO:0099068	postsynapse	4/652	34/28814	0.00703118	0.04296344	0.0323099	Nlgn1/Ntrk3/Nrxn1/Cdh2	4

GO:0099563	modification of synaptic	4/652	34/28814	0.00703118	0.04296344	0.0323099	Kalrn/Gripap1/Cttnbp2/Ctnna2	4
GO:0007422	peripheral nervous system	6/652	75/28814	0.00705882	0.04299861	0.0323364	Nefh/Nfasc/Plxna4/Nrg1/Pard3/Ntrk3	6
GO:0014068	positive regulation of phosphatidylino	6/652	75/28814	0.00705882	0.04299861	0.0323364	Nrg1/Ppard/Ntrk3/Insr/Igf1r/Erbb4	6
GO:0051495	positive regulation of	11/652	205/28814	0.00728988	0.0438127	0.0329486	Nav3/Bcas3/Ccdc88a/Akap9/Pde4dip/Limch1/Clip1/Bin1/Tenm1/Ntrk3/Mapt	11
GO:0014033	neural crest cell	7/652	99/28814	0.00729106	0.0438127	0.0329486	Fam172a/Tcof1/Sema3a/Kitl/Ext1/Erbb4/Cdh2	7
GO:0003128	heart field	3/652	18/28814	0.00730398	0.0438127	0.0329486	Robo2/Robo1/Ext1	3
GO:0015701	bicarbonate	3/652	18/28814	0.00730398	0.0438127	0.0329486	Slc4a10/Slc4a4/Slc4a3	3
GO:0021702	cerebellar Purkinje cell	3/652	18/28814	0.00730398	0.0438127	0.0329486	Herc1/Foxp2/Lhx1	3
GO:0030575	nuclear body	3/652	18/28814	0.00730398	0.0438127	0.0329486	Srpk2/Aff2/Daxx	3
GO:0060134	prepulse	3/652	18/28814	0.00730398	0.0438127	0.0329486	Chd8/Nrxn1/Ctnna2	3
GO:0060746	parental	3/652	18/28814	0.00730398	0.0438127	0.0329486	Kalrn/Brinp1/Gnaq	3
GO:1905244	regulation of modification of	3/652	18/28814	0.00730398	0.0438127	0.0329486	Kalrn/Gripap1/Cttnbp2	3
GO:1905939	regulation of gonad	3/652	18/28814	0.00730398	0.0438127	0.0329486	Zfpm2/Sema3a/Insr	3
GO:0021545	cranial nerve	5/652	54/28814	0.00741385	0.044269	0.0332917	Chd7/Plxna4/Adarb1/Sema3a/Ext1	5
GO:0035272	exocrine system	5/652	54/28814	0.00741385	0.044269	0.0332917	Sema3a/Polb/Insr/Igf1r/Fgfr2	5
GO:0045773	positive regulation of	5/652	54/28814	0.00741385	0.044269	0.0332917	Nrg1/Golga4/Ntrk3/Mapt/Dscam	5
GO:0140056	organelle localization by	6/652	76/28814	0.00752097	0.04484053	0.0337215	Rims2/Exoc6/Syt1/Exoc4/Nrxn1/Ncam1	6
GO:0031334	positive regulation of protein-	11/652	206/28814	0.00754901	0.04493948	0.033796	Nav3/Nrg1/Akap9/Pde4dip/Clip1/Bin1/Abca3/Tenm1/Tlr4/Mapt/Hsp90aa1	11
GO:0002064	epithelial cell development	12/652	235/28814	0.00761143	0.0452425	0.0340238	Jmjd1c/Akap9/Arid4b/Pard3/Atrx/Rock1/Plcb1/Afdn/Fgfr2/Col18a1/Cdh2/Arntl	12
GO:0090140	regulation of mitochondrial	4/652	35/28814	0.00779893	0.04621717	0.0347568	Tmem135/Spire1/Pparg/Mapt	4

GO:1903203	regulation of oxidative	4/652	35/28814	0.00779893	0.04621717	0.0347568	Ncoa7/Fbxw7/Tlr4/Parp1	4
GO:0001654	eye development	18/652	421/28814	0.00795764	0.04708667	0.0354107	Sdk1/Chd7/Ttll5/Gpm6a/Kmt2c/Foxp2/Pds5b/Ahi1/Tenm3/Zeb1/Ntrk3/Mitf/Lhx1/Hmgb1/Hipk2/Fgfr2/Dscam/Calb1	18
GO:0001662	behavioral fear	5/652	55/28814	0.00800686	0.04716487	0.0354695	Kalrn/Atp1a2/Spire1/Brinp1/Grik2	5
GO:0034605	cellular	5/652	55/28814	0.00800686	0.04716487	0.0354695	Stac/Mapt/Hsp90aa1/Hsp90ab1/Daxx	5
GO:0055010	ventricular cardiac muscle	5/652	55/28814	0.00800686	0.04716487	0.0354695	Chd7/Nrg1/Zfpm2/Ryr2/Fgfr2	5
GO:0050795	regulation of	7/652	101/28814	0.00810714	0.04768408	0.03586	Nlgn1/Sgip1/Ahi1/Nrxn1/Insr/Zfhx3/Alb	7
GO:0030307	positive regulation of	11/652	209/28814	0.00836963	0.04912409	0.0369429	Nrg1/Rims2/Golga4/Syt2/Syt1/Itsn2/Sgk1/Ntrk3/Mapt/Afdn/Dscam	11
GO:0051101	regulation of DNA binding	8/652	127/28814	0.00840895	0.04912409	0.0369429	Brd4/Fbxw7/Tlr4/Pparg/Nsd1/Hmgb1/Hipk2/Parp1	8
GO:0002790	peptide secretion	14/652	298/28814	0.00842071	0.04912409	0.0369429	Kalrn/Chd7/Rims2/Gabbr1/Pclo/Pparg/Ppard/Pde1c/Nrxn1/Myt1/Smad2/Epha5/Cacna1d/Arntl	14
GO:0150063	visual system development	18/652	424/28814	0.0085249	0.04912409	0.0369429	Sdk1/Chd7/Ttll5/Gpm6a/Kmt2c/Foxp2/Pds5b/Ahi1/Tenm3/Zeb1/Ntrk3/Mitf/Lhx1/Hmgb1/Hipk2/Fgfr2/Dscam/Calb1	18
GO:0021783	preganglionic parasympathetic	3/652	19/28814	0.00852946	0.04912409	0.0369429	Plxna4/Adarb1/Sema3a	3
GO:0035641	locomotory exploration	3/652	19/28814	0.00852946	0.04912409	0.0369429	Lsamp/Atp1a2/Slc4a10	3
GO:0045821	positive regulation of	3/652	19/28814	0.00852946	0.04912409	0.0369429	Slc4a4/Esrrb/Insr	3
GO:0045836	positive regulation of	3/652	19/28814	0.00852946	0.04912409	0.0369429	Eif4g3/Plcb1/Camk2b	3
GO:0045945	positive regulation of transcription	3/652	19/28814	0.00852946	0.04912409	0.0369429	Dek/Chd8/Ddx21	3
GO:0060123	regulation of growth hormone	3/652	19/28814	0.00852946	0.04912409	0.0369429	Kalrn/Chd7/Gabbr1	3
GO:0070875	positive regulation of	3/652	19/28814	0.00852946	0.04912409	0.0369429	Esrrb/Insr/Hmgb1	3

GO:0099638	endosome to plasma membrane	3/652	19/28814	0.00852946	0.04912409	0.0369429	Snx27/Grip1/Gripap1	3
GO:1900452	regulation of long-term	3/652	19/28814	0.00852946	0.04912409	0.0369429	Dgki/Ppp1r9a/Mapt	3
GO:1905874	regulation of postsynaptic	3/652	19/28814	0.00852946	0.04912409	0.0369429	Ntrk3/Nrxn1/Cdh2	3
GO:0036473	cell death in response to	7/652	102/28814	0.00853951	0.04912409	0.0369429	Ncoa7/Sfpq/Arl6ip5/Fbxw7/Tlr4/Mapt/Parp1	7
GO:0008045	motor neuron	4/652	36/28814	0.00861975	0.04929527	0.0370717	Plxna4/Mycbp2/Sema3a/Lhx1	4
GO:0048169	regulation of long-term	4/652	36/28814	0.00861975	0.04929527	0.0370717	Nptn/Grin2a/Grik2/Camk2b	4
GO:0120316	sperm flagellum	4/652	36/28814	0.00861975	0.04929527	0.0370717	Ttll5/Armc2/Spag16/Dzip1	4
GO:0002209	behavioral	5/652	56/28814	0.00863201	0.04929527	0.0370717	Kalrn/Atp1a2/Spire1/Brinp1/Grik2	5
GO:0071709	membrane	5/652	56/28814	0.00863201	0.04929527	0.0370717	Il1rapl1/Nlgn1/Nrxn1/Cdh2/Ank3	5
GO:0060485	mesenchyme development	14/652	299/28814	0.00865724	0.04936765	0.0371261	Robo2/Fam172a/Wnt2/Tcof1/Sema3a/Exoc4/Rock1/Robo1/Kitl/Smad2/Fgfr2/Ext1/Erbb4/Cdh2	14
GO:0042176	regulation of protein catabolic	17/652	393/28814	0.00873052	0.04971332	0.037386	Nell1/Hecw2/Nrg1/Mycbp2/Hecw1/Usp8/Fbxw7/Desi1/Sh3d19/Oaz2/Map1a/Hsp90aa1/Hsp90ab1/Hipk2/Grin2a/Fhit/Arntl	17
