# Supplementary figures and images for: Toll-like receptor 4 deficiency in Purkinje neurons drives cerebellar ataxia by impairing the BK channel-mediated after-hyperpolarization and cytosolic calcium homeostasis
Source: Cell Death Dis. 2024 Aug 15;15(8):594. doi: 10.1038/s41419-024-06988-w (PMC11327311; doi:10.1038/s41419-024-06988-w)

MaxiKα


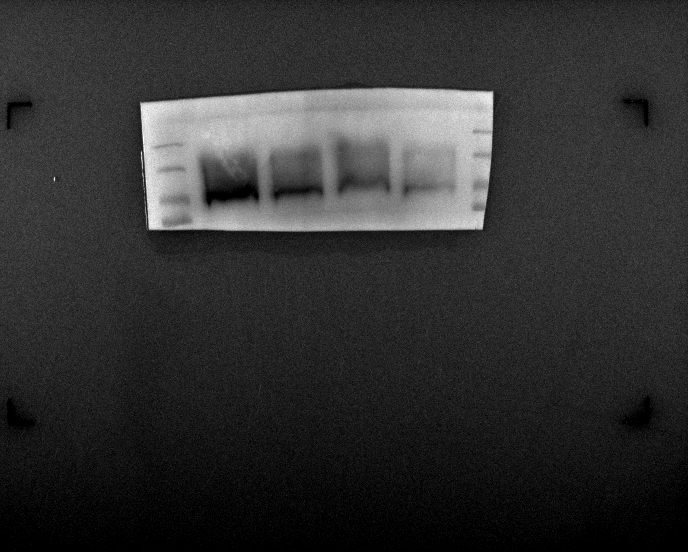


GAPDH


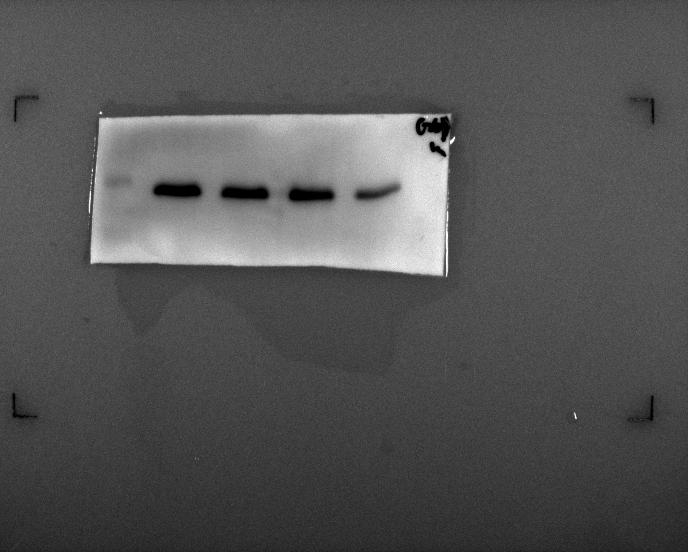

Supplement: Supplementary file 6 — original western blot [file 41419_2024_6988_MOESM6_ESM.docx]
